# Is there a duration-characteristic relationship for trypsin exposure on tendon? A study on anterior cruciate ligament reconstruction in a rabbit model

**DOI:** 10.3389/fmed.2024.1417930

**Published:** 2024-08-21

**Authors:** Rongxing Ma, Xiaokang Gao, Yangyang Jin, Xiaolong Wang, Ruifeng Li, Ruiqi Qiao, Xinliang Wang, Dayong Liu, Zhitao Xie, Limin Wang, Jingyu Zhang, Weiguo Xu, Yongcheng Hu

**Affiliations:** ^1^Clinical School/College of Orthopedics, Tianjin Medical University, Tianjin, China; ^2^Tianjin Hospital, Tianjin University, Tianjin, China; ^3^The People's Hospital of Chengyang Qingdao, Qingdao, Shandong, China; ^4^Department of Spine Surgery, Weifang People’s Hospital, Weifang, China; ^5^Department of Orthopedics, Affiliated Hospital of Hebei Engineering University, Handan, Hebei, China; ^6^Beijing Wonderful Medical Biomaterials Co., Ltd., Beijing, China

**Keywords:** allografts, tendon, trypsin, anterior cruciate ligament, healing

## Abstract

**Background:**

Decellularized allograft tendons are highly regarded for their accessibility and the reduced risk of immune rejection, making them a promising choice for grafting due to their favorable characteristics. However, effectively integrating reconstructed tendons with host bone remains a significant clinical challenge.

**Purpose:**

This study aims to investigate the relationship between the duration of tendon exposure to trypsin and its impact on tendon biomechanical properties and healing capacity.

**Methods:**

Morphological assessments and biochemical quantifications were conducted. Allograft tendons underwent heterotopic transplantation into the anterior cruciate ligament (ACL) in a rabbit model, with specimens harvested 6 weeks post-surgery for a comparative analysis of cell adhesion strength and mechanical performance. Duration-response curves were constructed using maximum stress and cell adhesion quantity as primary indicators.

**Results:**

The trypsin treatment enhanced cell adhesion on the tendon surface. Adhesion rates in the control group vs. the experimental groups were as follows: 3.10 ± 0.56% vs. 4.59 ± 1.51%, 5.36 ± 1.24%, 6.12 ± 1.98%, and 8.27 ± 2.34% (*F* = 6.755, *p* = 0.001). However, increasing treatment duration led to a decline in mechanical properties, with the ultimate load (N) in the control vs. experimental groups reported as 103.30 ± 10.51 vs. 99.59 ± 4.37, 93.15 ± 12.38, 90.42 ± 7.87, and 82.68 ± 6.89, *F* = 4.125 (*p* = 0.013).

**Conclusion:**

The findings reveal an increasing trend in adhesion effectiveness with prolonged exposure duration, while mechanical strength declines. The selection of the optimal processing duration should involve careful consideration of the benefits derived from both outcomes.

## Introduction

Tendon injuries are common in orthopedics, often leading to significant pain and disability (1). When direct repair is impractical, reconstruction becomes necessary, especially when the injury surpasses the optimal repair period or involves extensive soft tissue scarring (2, 3). Tendon allografts are preferred for reconstruction due to their easy availability, the absence of donor site complications, and shorter operative times (4). However, allografts contain viable cells that may provoke an immune response, necessitating decellularization to reduce immunogenicity (5, 6). Among various decellularization agents, trypsin is widely utilized for its enzymatic properties (7).

For example, Koo et al. successfully decellularized human tissue using trypsin-EDTA combined with ultrasound, achieving complete decellularization while preserving a microporous lipid bilayer (8). Similarly, Sánchez et al. employed a combination of trypsin, Triton X-100, and DNase to achieve complete decellularization and maintain remarkable scaffold tensile strength (9). The control over exposure duration and the modulation of extracellular matrix (ECM) response are more effectively managed with trypsin (10). Therefore, we selected trypsin as our decellularization agent to remove cellular and extracellular matrix components from intrasynovial tendon allografts.

The healing of tendon-bone interfaces relies significantly on the migration of cells from the surrounding tissue into the graft to facilitate tissue regeneration (11). The intrasynovial tendon surface has an oval cross-section and is lined with a thin layer of aponeurotic cells that secrete lubricants such as hyaluronic acid and lubricin (12). However, lubricin, a lubricating glycoprotein, is known to inhibit cell adhesion (13, 14). Interestingly, lubricin also acts as an anti-adhesion protein, inhibiting synoviocyte overgrowth *in vitro* and *in vivo* (15).

Rhee et al. conducted experiments with lubricin mutant mice, expressing lubricin in chondrocytes and synoviocytes, and found that it effectively inhibited synoviocyte growth *in vitro* (16). The presence of lubricin reduces the adhesive growth of tenocytes, thereby delaying healing. Fortunately, the trypsin used for decellularization can remove lubricin. Trypsin digestion exposes the meshwork of the tendon surface, enhancing cell adhesion and potentially facilitating tendon-to-bone healing (11). Additionally, Hashimoto et al. demonstrated that trypsinization increases surface roughness and friction on the tendon surface, thereby providing a larger area for cell attachment. Since lubricants possess anti-adhesive properties, their removal from the graft surface could promote better graft integration (11, 13). Therefore, the trypsin decellularization process not only eliminates cellular components but also enhances tendon healing following graft placement.

Tendon injuries remain a significant challenge in orthopedic medicine, often necessitating careful consideration of treatment protocols to avoid complications such as tissue damage and matrix remodeling. Recent studies underscore the critical role of lubricin in joint protection and highlight innovative approaches in tissue engineering. For instance, advancements in decellularization techniques using trypsin-EDTA have demonstrated effective preservation of the tendon structure and mechanical properties (16, 17). Similarly, research on acellular dermal matrices reveals promising outcomes in animal models, emphasizing safety and functionality (18).

Moreover, investigations into tendon-to-bone healing highlight the importance of optimized trypsin treatment durations to minimize lubricin loss and enhance tissue integration (19). In this context, selecting appropriate treatment parameters is crucial for maintaining tendon integrity and optimizing therapeutic outcomes.

In this context, understanding the clinical implications of our findings is crucial. Effective decellularization with trypsin holds the potential to reduce immunogenicity, promote cell adhesion, and improve the likelihood of successful tendon healing. By elucidating the relationship between trypsin exposure duration and the effects on tendons, our research aims to provide information on evidence-based practices that improve tendon allograft performance and patient recovery, which could inform and refine surgical practices and ultimately lead to better outcomes for patients receiving tendon allografts.

## Materials and methods

### Grouping principles and methods

Our duration and grouping were rigorously summarized and designed based on previous research results. Zhou et al. effectively decellularized bovine tendon using 0.05% trypsin/5 mM EDTA at 37°C for 5 h (17). Qu et al. treated rabbit tendons with 0.25% trypsin/0.53 mM EDTA and found that, after 30 or 60 min of trypsinization, no lubricin was evident on the surface and ECM (19). Based on the aforementioned analysis and the tendon type we selected, we opted for treatment with 0.25% trypsin for a maximum duration of 30 min.

The grouping principle of the present study was based on the duration of trypsin treatment for *in vitro* decellularization, with the maximum duration set at 30 min and segmentation values set at 18 min, 11 min, and 7 min, following the golden section grouping method. Tendons were divided into five groups: 0 min (control group), 7 min, 11 min, 18 min, and 30 min trypsin treatment groups. Six tendons per group were utilized for morphological and biochemical assessments. Eight tendons per group were employed for allograft transplantation of the anterior cruciate ligament (ACL) and for postoperative evaluations.

### Fresh tendon harvesting

**Figure 1:**
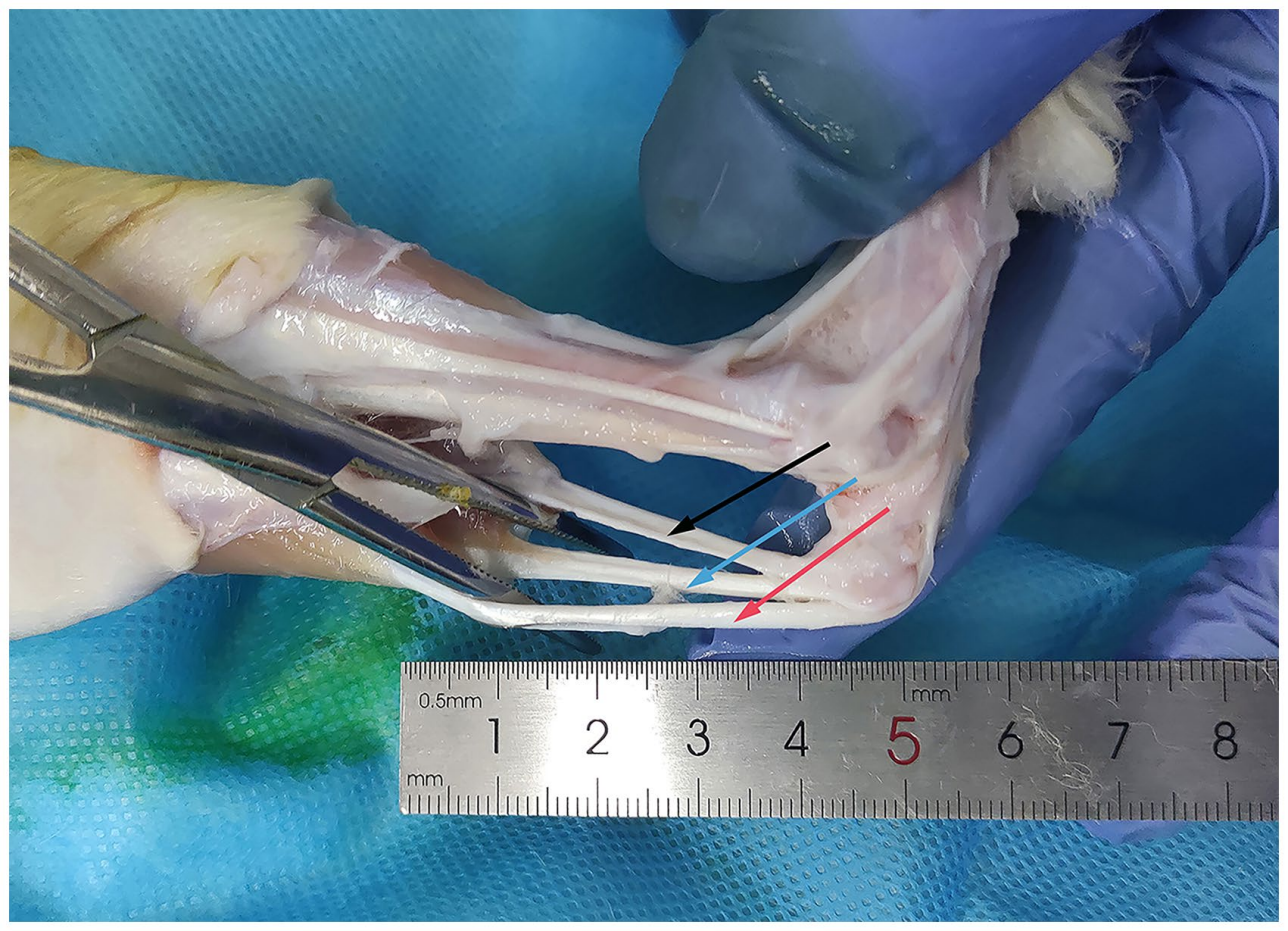
Acquisition of rabbit Achilles tendons. The Achilles tendon of the rabbit is exposed using hemostatic forceps. The black arrow indicates the calcaneal tendon: connecting the rabbit’s calf muscles to the calcaneus. The red arrow indicates the gastrocnemius tendon: situated on the lateral aspect of the calcaneal tendon, connecting to the gastrocnemius muscle. The blue arrow indicates the plantaris tendon: the third smaller tendon, typically located between the calcaneal tendon and the gastrocnemius tendon.

We used healthy New Zealand white rabbits aged 6–8 months old and weighing 2–2.5 kg in this study. The protocol was approved by the Tianjin Hospital Institutional Animal Care and Use Committee (Ethics Approval Number: 2022 MedEthics 106, Supplementary material 1). Rabbits were euthanized, and a total of 30 Achilles tendons were collected aseptically. Muscles and paratendon were removed, and the tendons were stored in sterile bags and frozen at −80°C (SC-41, Zhongke Meiling Low-Temperature Technology Co., Ltd., Hefei, China, Figure 1).

### Preparation of decellularized tendons

The tendons were thawed and washed thrice with saline (20 min each) to remove tissue residues. Pulse washing with water eliminated blood and fat. The tendons were then sectioned into lengths of 5–8 cm and widths of 0.5–1 cm, frozen at −80°C, and stored for >30 days. The tendons were placed in 75% ethanol (Shandong Lierkang Technology Co., Ltd., China) and shaken for 20 min, with the solution changed 2–3 times. Subsequently, they were immersed in PBS buffer (20 min, pH 7.2–7.4, 0.01 M, Beijing Solebao Technology Co., Ltd., China). Hydrogen peroxide 1% (Beijing Solebao Technology Co., Ltd., China) was applied, shaken, and rinsed for 1 h, with the solution changed every 15 min. The tendons were then immersed in 95% ethanol (Beijing Solebao Technology Co., Ltd., China) and shaken for 48 h, with the solution changed every 12 h.

A solution of A/B peracetic acid (1:1 ratio, 15–18%, w/v, Shandong Anjie High-tech Technology Co., Ltd., China) was prepared and mixed at room temperature for 24 h. The application solution consisted of A/B, ethanol, and distilled water at a ratio of 0.3:24:75.7. The tendons were immersed in this solution for 30 min before being washed with PBS (pH 5.5–7.5) and purified water.

Following the golden ratio, we selected five duration points between 0 min and 30 min for trypsin exposure: 0 min, 7 min, 11 min, 18 min, and 30 min. The tendons were immersed in a trypsin/EDTA solution (0.25% w/v trypsin; 0.02% w/v EDTA, Beijing Solabio) at 37°C for the aforementioned durations. Following digestion, the tendons were neutralized using high-glucose DMEM medium (500 mL, Beijing Solabio Co., Ltd.) containing 10% fetal bovine serum, 100 IU/mL penicillin, 100 μg/mL streptomycin, and 0.25 mg/mL amphotericin B. The tendons were then subjected to two rounds of oscillation washing for 30 min each to remove residual trypsin.

**Figure 2:**
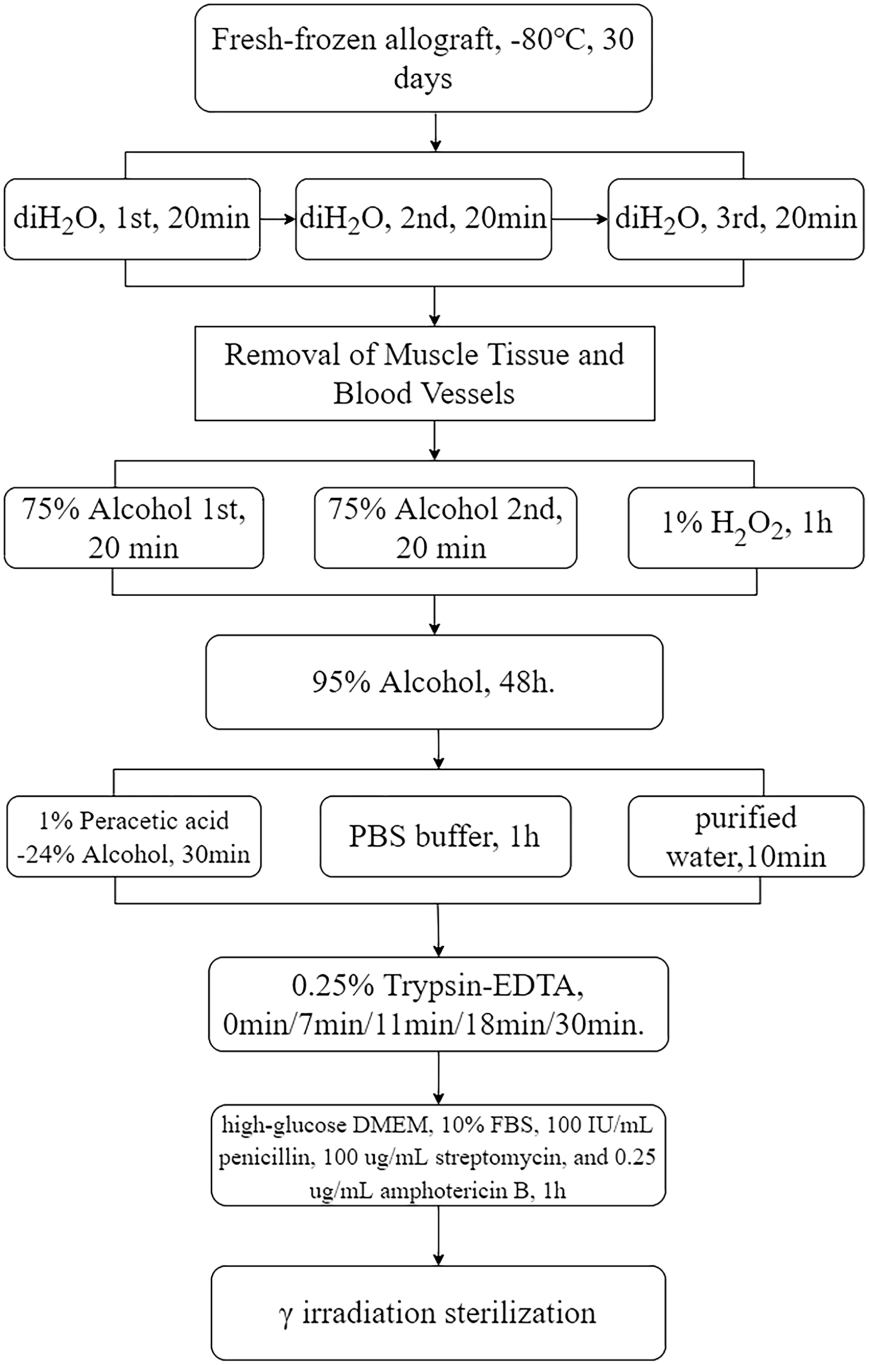
Tendon processing flowchart. Rounded rectangles denote the methods employed, whereas rectangular boxes represent the specific procedural steps, with arrows distinctly indicating the direction of the process flow, delineating the sequential progression from one step to another. diH_2_O, deionized water; DMEM, Dulbecco’s Modified Eagle’s medium.

After draining surface moisture, the tissue was cryogenically pre-frozen at −80°C. The tissue was then γ-irradiated at 25 kGy under dry ice with a ^60^Co source (Beijing Wonderful Medical Biomaterials Co., Ltd.). The tendons were aseptically packaged in sterile tubes and stored at −80 ± 3°C (Figure 2).

### Morphological observation

For each group, six samples were longitudinally sliced into several 1 mm^3^ sections. Staining was performed using an HE staining kit (150/3*10 mL, G1120, Beijing Solabio Technology Co., Ltd.). For Alcian blue staining, tendons were stained using an Alcian blue staining kit (370/3*50 mL, G1560, Beijing Solabio Technology Co., Ltd.). Scanning electron microscopy (Hitachi Regulus 8100) was utilized for observation. The main steps included sample preparation, sample placement under vacuum, specimen exposure to the electron beam, sample positioning, and the adjustment of imaging parameters such as magnification and scale.

### Biochemical analysis

The lubricin ELISA assay kit (LZ-E99758, Shanghai Lianzu Biological Technology Co., Ltd.) was employed for measurement. Blank and standard sample wells were prepared. In the standard well, 50 μL of the sample was added, and in the sample well, 10 μL of the sample was added. After incubation, the enzyme-labeled reagent was added, followed by chromogenic reagents. The optical density (OD) at 450 nm was measured for content calculation. The glycosaminoglycan (GAG) content was measured using the 1,9-dimethylmethylene blue (DMMB, Hepeng Biotechnology) assay. Samples were diluted in Phosphate Buffered (PBE), The DMMB dye was added, and the absorbance shift (*λ* = 525–595 nm) was measured for GAG content calculation. The BCA protein concentration determination assay kit (P0012, 500T, Beyoduration, China) was utilized for measurement.

### Anterior cruciate ligament (ACL) transplantation

A total of 20 male New Zealand white rabbits underwent bilateral surgery. Animal weights were measured using an electronic scale. Anesthesia was induced with a 0.2 mL/kg injection of ketamine hydrochloride, followed by maintenance with isoflurane. Preoperative prophylaxis included an intramuscular injection of 400,000 units of sodium penicillin and flunixin meglumine.

The animals were positioned prone on the surgical table, and the knee joint area was sterilized. A longitudinal incision was made on the lateral side of the patella to expose the subcutaneous tissue and tendon sheath, which were then dissected to reveal the anterior cruciate ligament (ACL). The ACL was transected at the center of the joint capsule. Holes were drilled from the joint capsule position to the proximal edge of the femur and then downward to the proximal end of the tibia. A pre-hydrated graft tendon was retrieved, trimmed, and sutured using the modified Bunnell technique.

**Figure 3:**
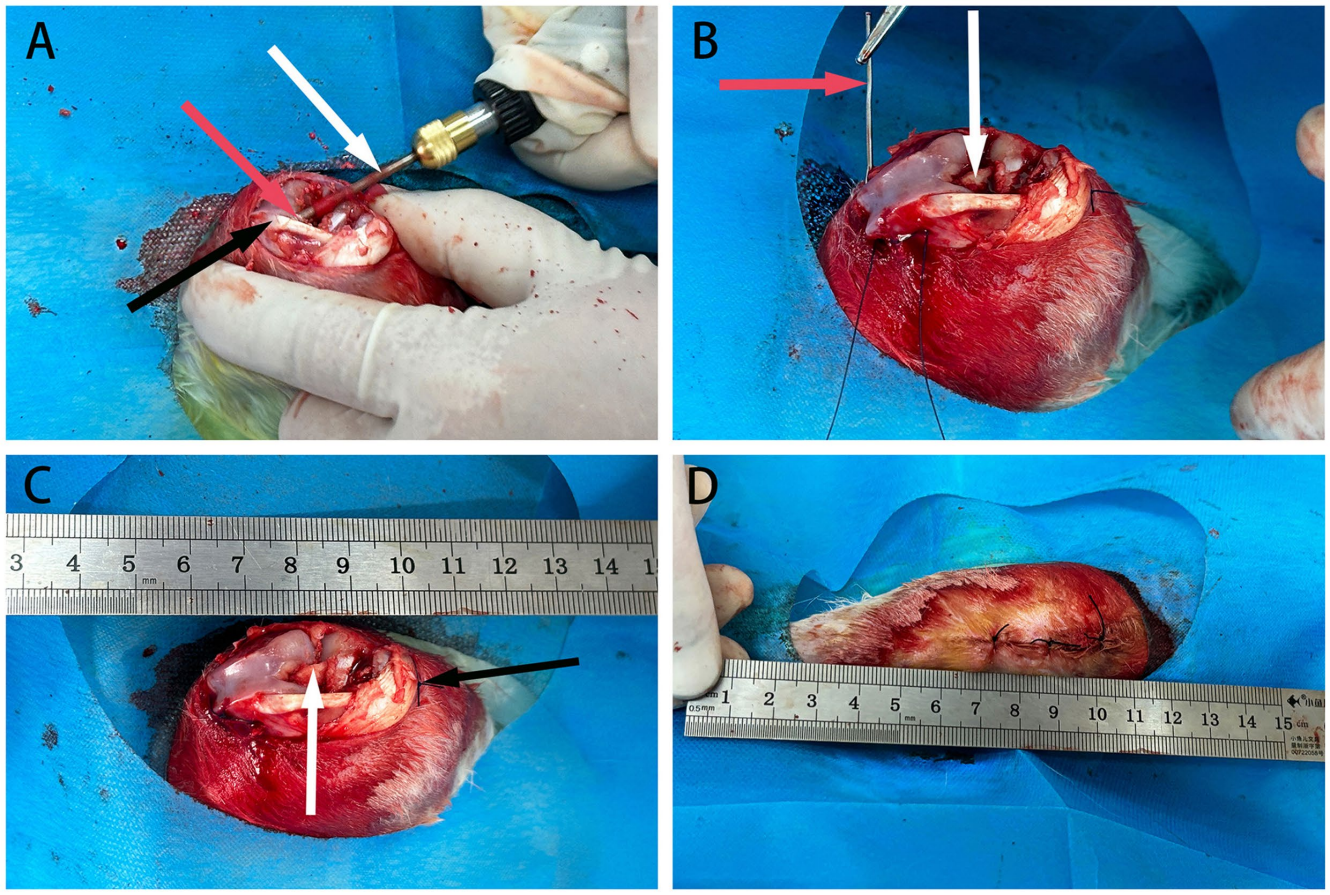
The schematic diagram of ACL reconstruction in rabbits. **(A)** Depicts bone marrow drilling using a perforator, indicated by white arrows, with red arrows indicating the femoral tunnel at the distal end and black arrows indicating the patellar ligament. **(B)** Shows the passage and fixation of allograft tendon using a guiding needle, represented by red arrows, with the allograft tendon already fixed at one end, indicated by white arrows. **(C)** Demonstrates the completed fixation of the allograft tendon at both ends, with white arrows indicating securely fixed tendons. **(D)** Displays the layers of knee joint tissues fully sutured.

The tendon ends were secured to the quadriceps femoris and gastrocnemius muscles, ensuring appropriate tension. The surgical site was irrigated with sterile saline, and the wounds were closed. Postoperatively, intramuscular injections of 400,000 units of sodium penicillin and flunixin meglumine were administered for anti-inflammatory and analgesic purposes (Figure 3).

After surgery, animals were returned to cages upon awakening. They received daily intramuscular injections of 400,000 units of sodium penicillin and flunixin meglumine, with wound dressings changed for three consecutive days.

The successful establishment of the rabbit ACL transplantation model was confirmed by the absence of unexpected deaths, suture failures, significant neurovascular damage, wound or joint contamination, and unrestricted passive knee dorsiflexion and plantarflexion. We only selected tendons from successful ACL transplantations for sampling.

**Figure 4:**
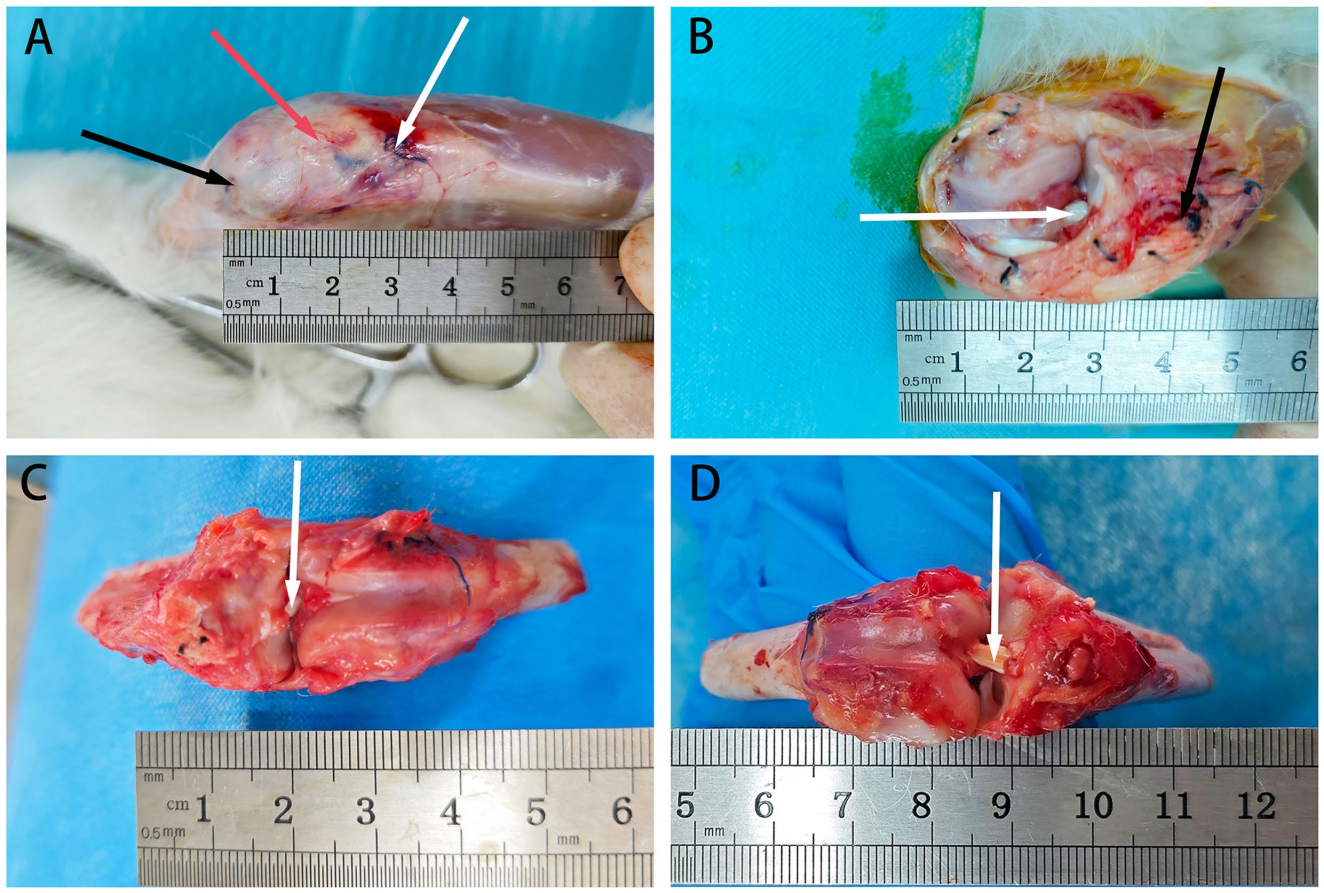
The retrieval of rabbit tendons at 6 weeks post-surgery. **(A)** Depicts the exposed field after skin removal, with black arrows indicating the proximal end of the bone tunnel’s suture, red arrows indicating the patellar tendon, and white arrows indicating the distal end of the bone tunnel’s suture. **(B)** Shows the field after excising the patellar tendon to expose the ACL graft, with white arrows indicating the allograft tendon and black arrows indicating the distal suture. **(C)** Displays the field after cutting the knee joint to expose the ACL, with white arrows indicating the allograft tendon. **(D)** Presents the field after exposing the ACL through flexion of the knee joint, with white arrows indicating the allograft tendon.

At 6 weeks post-surgery, ACL samples were obtained from each group after fasting from water. Euthanasia was performed, and a longitudinal incision was made on the lateral side of the knee joint. The tissues were dissected layer by layer to expose the allograft tendon. The tendon was excised approximately 1–2 cm above the junctions at both ends, rinsed with physiological saline, placed in sterile specimen bags, and stored at −80°C. Subsequent biomechanical and cell adhesion testing were conducted (Figure 4).

### Cell adhesion rate

We harvested the tendon from 1 to 2 cm proximal to the distal femoral and proximal tibial tunnels mentioned above. After removing the tendons, incomplete portions at both ends were excised. Subsequently, we obtained a tendon segment approximately 5 × 3 mm in size, centered at the midpoint of the tendon. To ensure direct transillumination observation, the tendon was then sliced into a single-layered sheet measuring approximately 3 × 2 mm.

Bone marrow mesenchymal stem cell (BMSC) Isolation and Reseeding: BMSCs were isolated from rabbit tibia bone marrow and cultured in a medium containing 10% FBS, 1% antibiotics, and Earle’s salt. The tendons were rehydrated, and BMSCs were reseeded at a concentration of 1 × 10^5^ cells/mL and cultured at 37°C with 5% CO_2_ for 1–2 weeks, with the medium changes every 3 days (11).

Cell Attachment Observation using Confocal Laser Microscope (KC-X1000, KathMatic, Nanjing Kaishimai Co., Ltd.): After 1–2 weeks, the medium was supplemented with calcein AM and ethidium homodimer-1. Following a 30-min incubation, the samples were washed and imaged using a confocal microscope (excitation: 488 nm, emission: 570/630 nm for dead, 500–550 nm for live cells). For each section, images were captured from five positions: the four corners and the center. Fiji (Version 1.54f 29 June 2023) was used to count cells. The adhesion rate was calculated (20).

### Biomechanical test

We harvested tendons at 6 weeks post-surgery, cutting them at the opening of the tunnels approximately 2 cm from the distal end of the femur and the proximal end of the tibia. We extracted the central portion of the tendon, removed any attached muscle tissue, trimmed it, and then conducted mechanical testing.

**Cross-sectional Area Analysis using Alginate Impression**: The tendon was bent and immersed in alginate. After solidification, alginate slices were obtained, and the average pore diameter was calculated using software.

**Cyclic Loading Test**: The tendon was secured with an initial distance set at 1 cm. To eliminate creep, a 2 N load was applied for 30 s. Subsequently, cyclic loading was performed at 0.2 Hz for 50 cycles, with loads ranging from 2 N to 10 N.

**Tensile Fracture Test**: The tendon was tensioned at 10 N for 3 min and then loaded at a rate of 10 mm/min. This procedure allowed for the plotting of load-elongation and stress–strain curves, from which mechanical properties such as stiffness and ultimate load were calculated.

### Statistical analysis

Continuous variables were presented as $\bar{X}\pm SD$ . Normal distribution and variance homogeneity were assessed. For normally distributed data, a one-way ANOVA was used to analyze the *F* effect, followed by the LSD post-hoc tests. The Kruskal–Wallis test was applied to analyze the *H* effect for non-normally distributed data. All analyses were conducted using SPSS 21.0, with a two-sided significance level set at *α* = 0.05.

## Results

### Morphological evaluation

**Figure 5:**
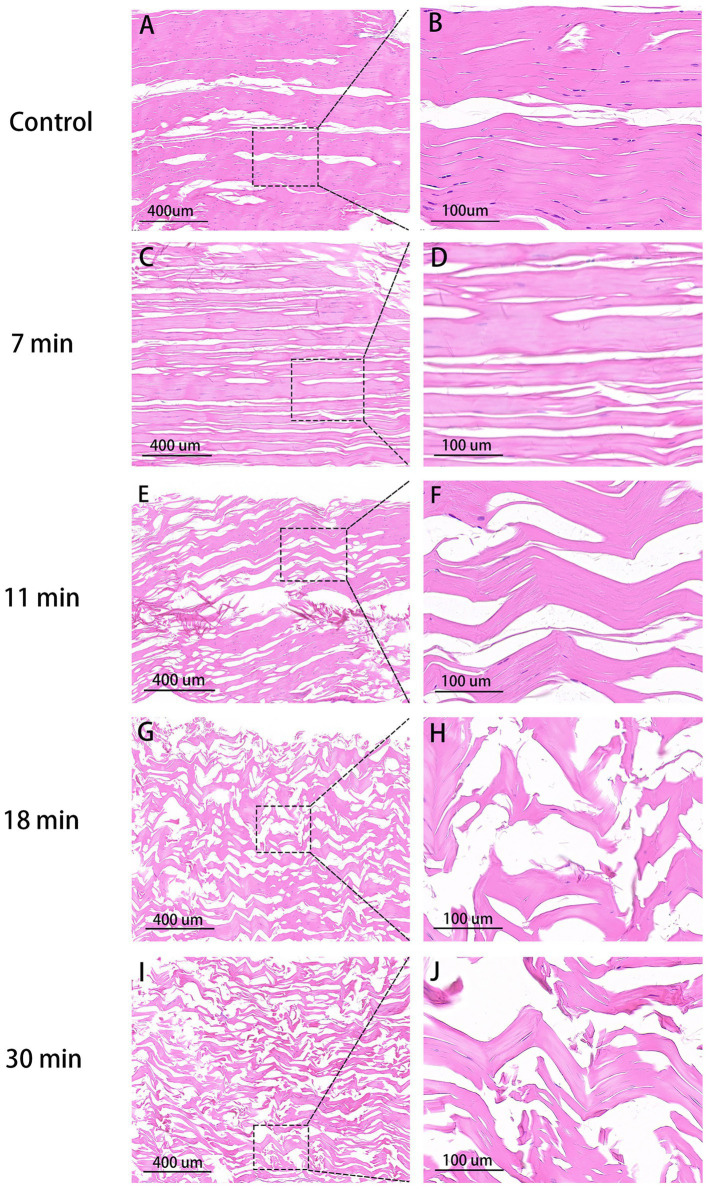
HE stain of tendons (×100, 400). Control group **(A,B)**: Organized collagen fibers, minimal interstitial space, intact nuclei stained purple. 7 min **(C,D)**: Increased interstitial space, aligned collagen, indistinct nuclei. 11 min **(E,F)**: Higher gap, slight fiber fracture. 18 min **(G,H)**: Disordered fibers, larger space, uneven bundle connections, reduced nuclei. 30 min **(I,J)**: Maximal disorder, widest gap, disrupted collagen.

Tendon Collagen Arrangement: In the blank control group, fibers are neatly aligned with narrow spaces between collagen tissues, the clear nuclei, and close connections between tendon bundles. In the 7-min group, collagen gaps enlarge and some fibers break, although a consistent pattern persists with faint nuclei, showing better collagen integrity compared to the latter three groups. In the 11-min and 18-min groups, collagen gaps further expanded, leading to significantly disordered fiber arrangement and inconsistent alignment. Most fibers exhibit signs of rupture, and connections between the tendon bundles loosen. In the 30-min group, collagen arrangement becomes highly disordered, with the largest interstitial spaces observed, and nearly all fibers experience rupture (Figure 5).

**Figure 6:**
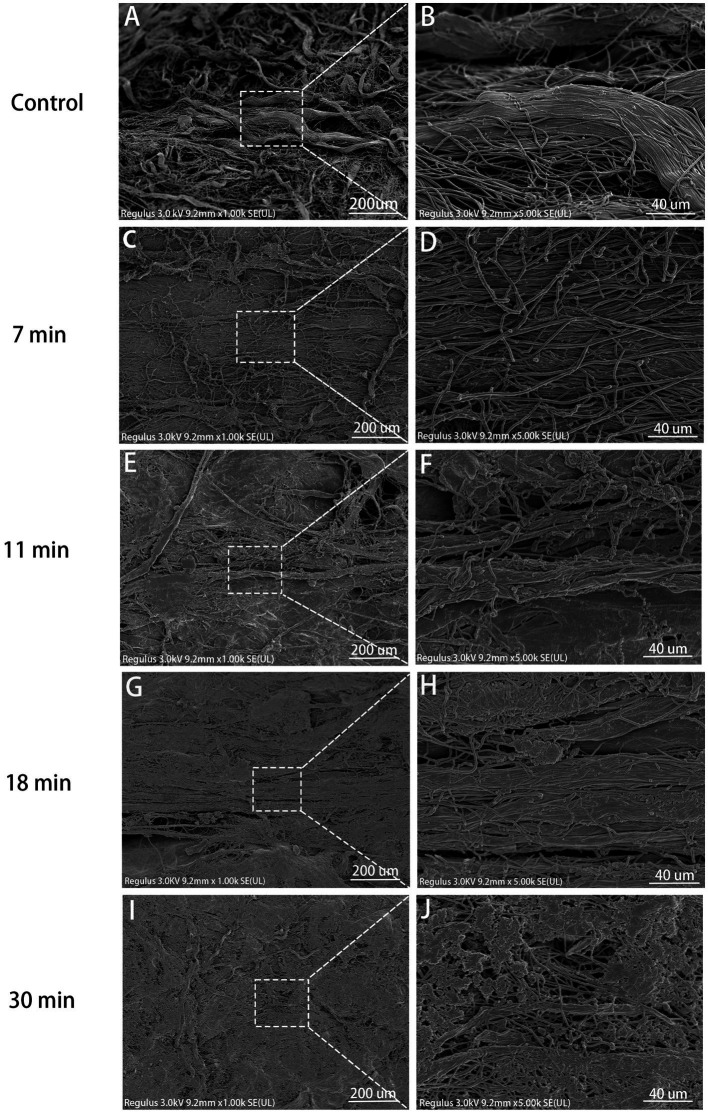
SEM view (×1,000, ×5,000). Control **(A,B)**: Steep collagen waves, visible fibrils, smooth surface. 7 min **(C,D)**: Collagen damage, crossing fibrils, distinct bundles. 11 min **(E,F)**: Severe destruction, less formed collagen, adherent fibrils. 18 min **(G,H)**: More pronounced destruction, rough fibril surface, no voids. 30 min **(I,J)**: Severe 3D damage, indistinct fibrils, voids on surface.

In the control group, collagen fibers were clearly visible, exhibiting distinct individuality with surface structures characterized by normal elevations and depressions. The tendons were composed of parallel alignment displayed a smooth surface of dense collagen fibers under high magnification. In the 7-min group, although normal interstitial spaces were disrupted and original fibers intertwined, they remained discernible under high magnification; however, in both the 11-min and 18-min groups, the three-dimensional structure of collagen suffered severe disruption. The original fibers interweaved, lost their form, and adhered to each other, rendering it impossible to distinguish individual fiber structures clearly under high magnification, resulting in an apparent mesh-like structure. Similarly, in the 30-min group, the destruction of collagen fiber spatial structure was most severe, rendering it impossible to discern the arrangement of original fibers clearly while also revealing the presence of hollow mesh-like structures on the collagen surface along with tearing-like gaps (Figure 6).

**Figure 7:**
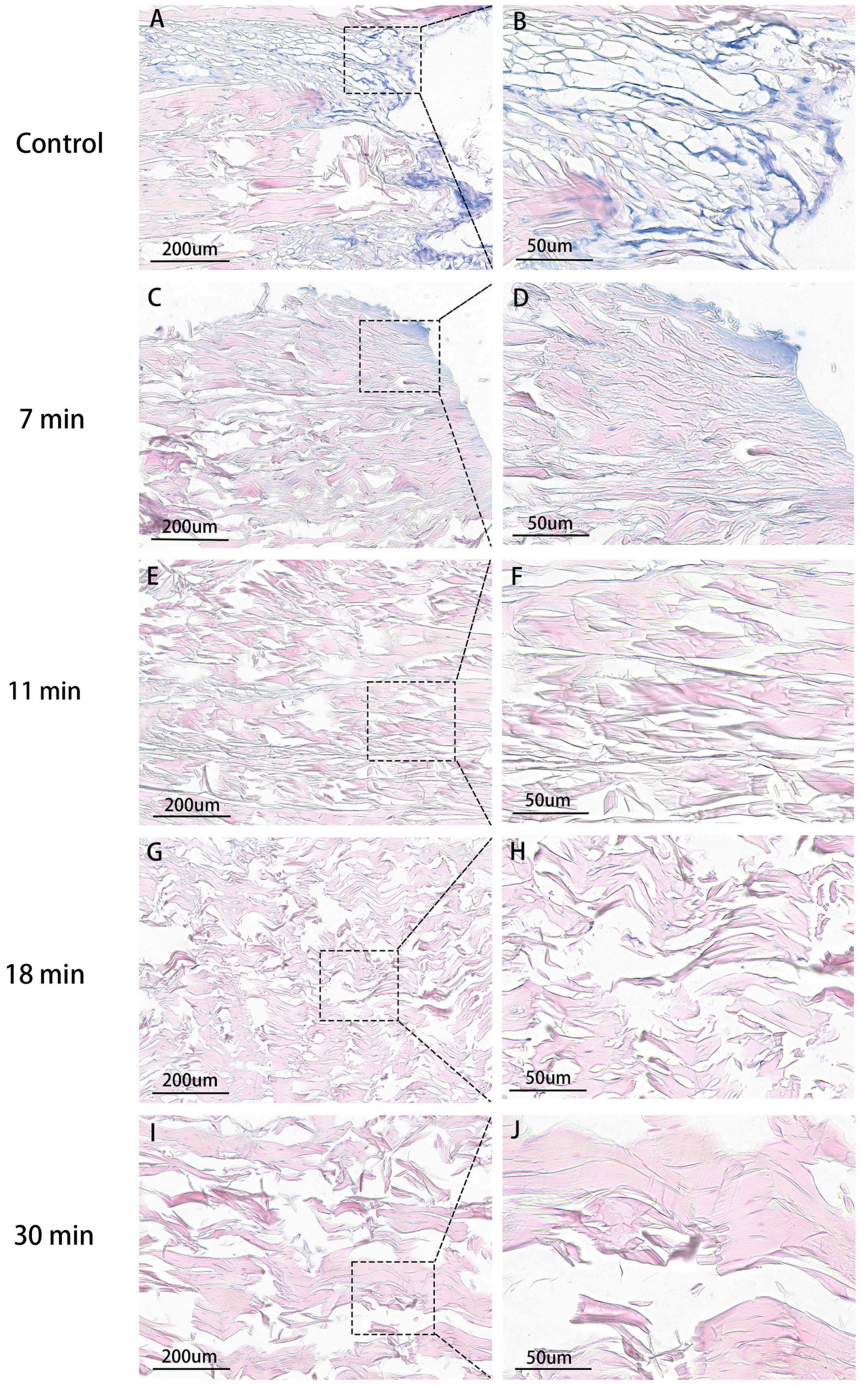
Tendon AB stain (×100, ×400). Control group **(A,B)**: Highest GAG content, extensive blue areas, intact fibrils. 7 min group **(C,D)**: Slight blue staining, lower than control. 11 min **(E,F)**, 18 min **(G,H)**, 30 min **(I,J)**: No visible blue staining. 30 min: Lowest average OD.

After staining with Alcian blue, the glycosaminoglycan (GAG) components of proteins in the tendon were stained light blue. The blue staining area was noticeably highest in the control group, decreased in the 7-min group compared to the control group but remained higher than the following four groups. The average optical densities of the control group, 7-min, 11-min, 18-min, and 30-min groups were 0.222 ± 0.013, 0.191 ± 0.021, 0.140 ± 0.019, 0.116 ± 0.011, and 0.071 ± 0.146, respectively. (*F* = 68.059, *p* < 0.001). Post-hoc tests revealed that the differences between the control group and the other four groups were all statistically significant (*p* < 0.001). This indicates that, with an increase in duration after treatment with trypsin, more GAG in tendon tissues is digested over time (Table 1 and Figure 7).

**Table 1:** Description and comparison of tenocyte number, adhesion rate, and optical density.

Group	Cell number	Cell adhesion rate	Optical density value
Control	724 ± 157	3.10 ± 0.56	0.222 ± 0.013
7 min	1,023 ± 139	4.59 ± 1.51	0.191 ± 0.021
11 min	1,257 ± 225	5.36 ± 1.24	0.130 ± 0.019
18 min	1,354 ± 244	6.12 ± 1.98	0.116 ± 0.011
30 min	1,505 ± 299	8.27 ± 2.34	0.071 ± 0.015
Results	*F* = 9.563	*F* = 6.755	*F* = 69.187
	*p < 0.001	*p = 0.001	*p < 0.001

Statistical analysis for each index was performed using a one-way ANOVA (effect size: *F*).*p < 0.05 indicates statistical significance.

### Biochemical measurements

**Figure 8:**
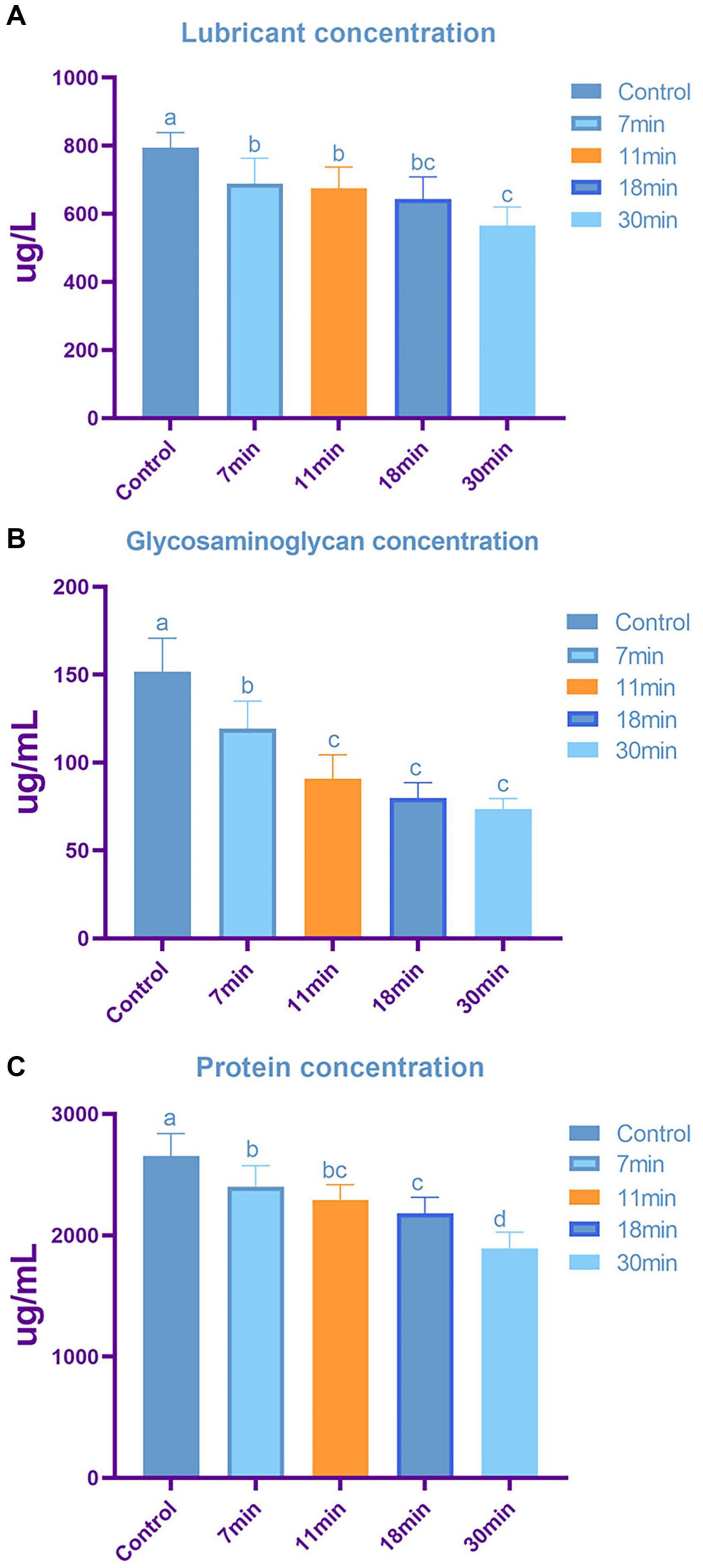
The content of tendon lubricin **(A)**, glycosaminoglycan **(B)**, and total protein **(C)** across different groups. Letters above each bar denote intergroup differences, where groups with the same letter indicate no significant difference, while groups with different letters indicate significant differences. The content of lubricin **(A)** is inversely proportional to the duration of trypsin treatment, with significant differences observed in tendon samples treated with trypsin after 7 min. Similarly, glycosaminoglycan **(B)** content shows a duration-dependent decrease, with minimal changes in GAG observed when the treatment duration exceeds 11 min. Total protein content **(C)** also exhibits a decreasing trend with increasing treatment duration.

Lubricin Concentration: The highest content was observed in the control group at 793.98 ± 44.87 ng/L, while the lowest content was in the 30-min group at 566.03 ± 54.01 ng/L. The lubricin content in the 7-min, 11-min, and 18-min groups were 689.68 ± 73.89 ng/L, 675.21 ± 62.28 ng/L, and 643.15 ± 65.66 ng/L, respectively (*F* = 9.165, *p* < 0.01). Post-hoc tests revealed statistically significant differences between the control group and the other four groups (Figure 8A).

The GAG content in the control group, 7-min, 11-min, 18-min, and 30-min groups was 151.70 ± 19.00 ug/mL, 119.41 ± 15.52 ug/mL, 90.92 ± 13.59 ug/mL, 79.81 ± 8.80 ug/mL, and 73.61 ± 5.84 ug/mL, respectively (*F* = 29.118, *p* < 0.01) (Figure 8B).

The total protein content in the control group, 7-min, 11-min, 18-min, and 30-min groups were 2,655.27 ± 182.93 ug/mL, 2,398.71 ± 174.73 ug/mL, 2,292.20 ± 125.37 ug/mL, 2,179 + 0.34 ± 133.91 ug/mL, and 1,892.24 ± 133.07 ug/mL, respectively (*F* = 17.093, *p* < 0.01). Post-hoc tests indicated statistically significant differences between the control group and the other four groups (Figure 8C).

Lubricin, GAG, and total protein contents were highest in the control group and lowest after 30 min of trypsin treatment, showing a negative correlation with treatment duration. Lubricin plays a crucial role in preventing cell adhesion and providing lubrication for tendons; excessive lubricin may hinder tendon repair. GAGs are critical for tendon integrity, with a compromised stromal immune response potentially affecting cell adhesion and ECM proteins.

### Cell adhesion rate

**Figure 9:**
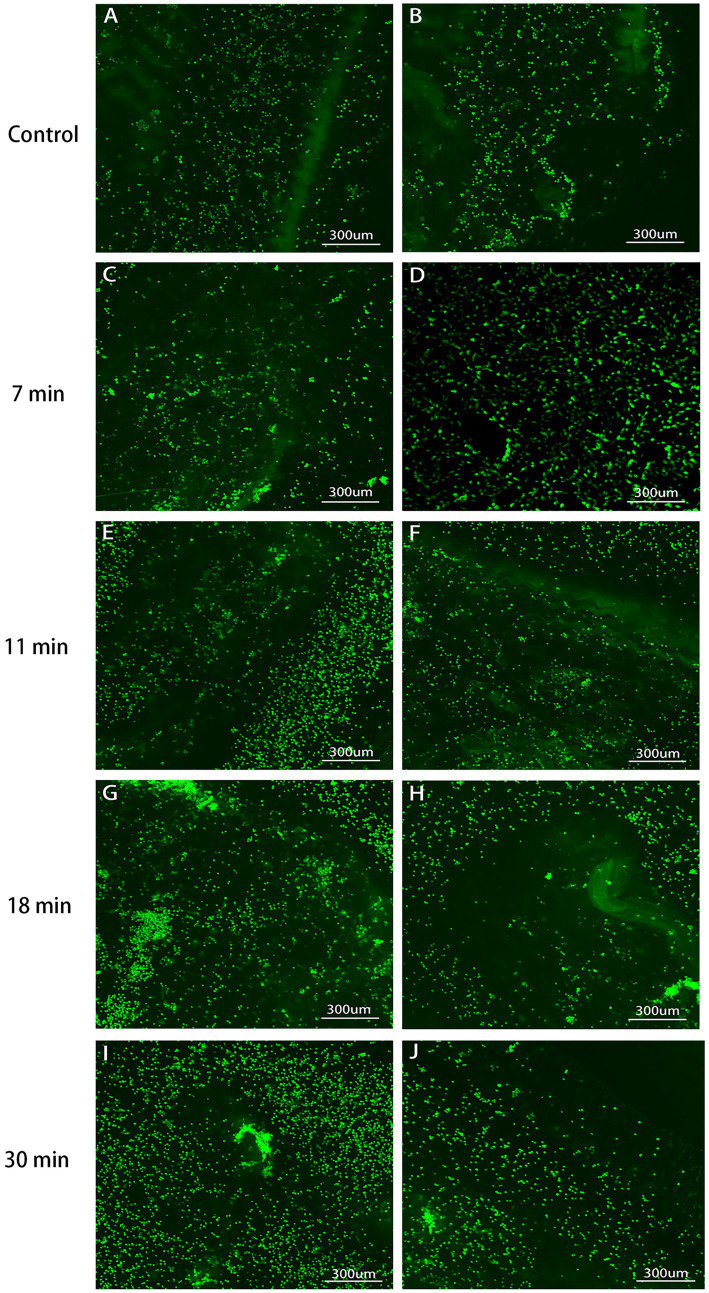
Tendon-BMSC adhesion after 1-week culture (×100, 400). Cultured tendons with LIVE/DEAD kit marked live cells green. Control **(A,B)**: Lowest attachment. The adhesion rates sequentially increased in the 7 min **(C,D)**, 11 min **(E,F),** 18 min **(G,H)**, and 30 min **(I,J)**: Maximum attachment, indicating the most favorable conditions for cell attachment and growth. This observation correlates with the mesh-like structure observed in tendon formation under scanning electron microscopy.

**Figure 10:**
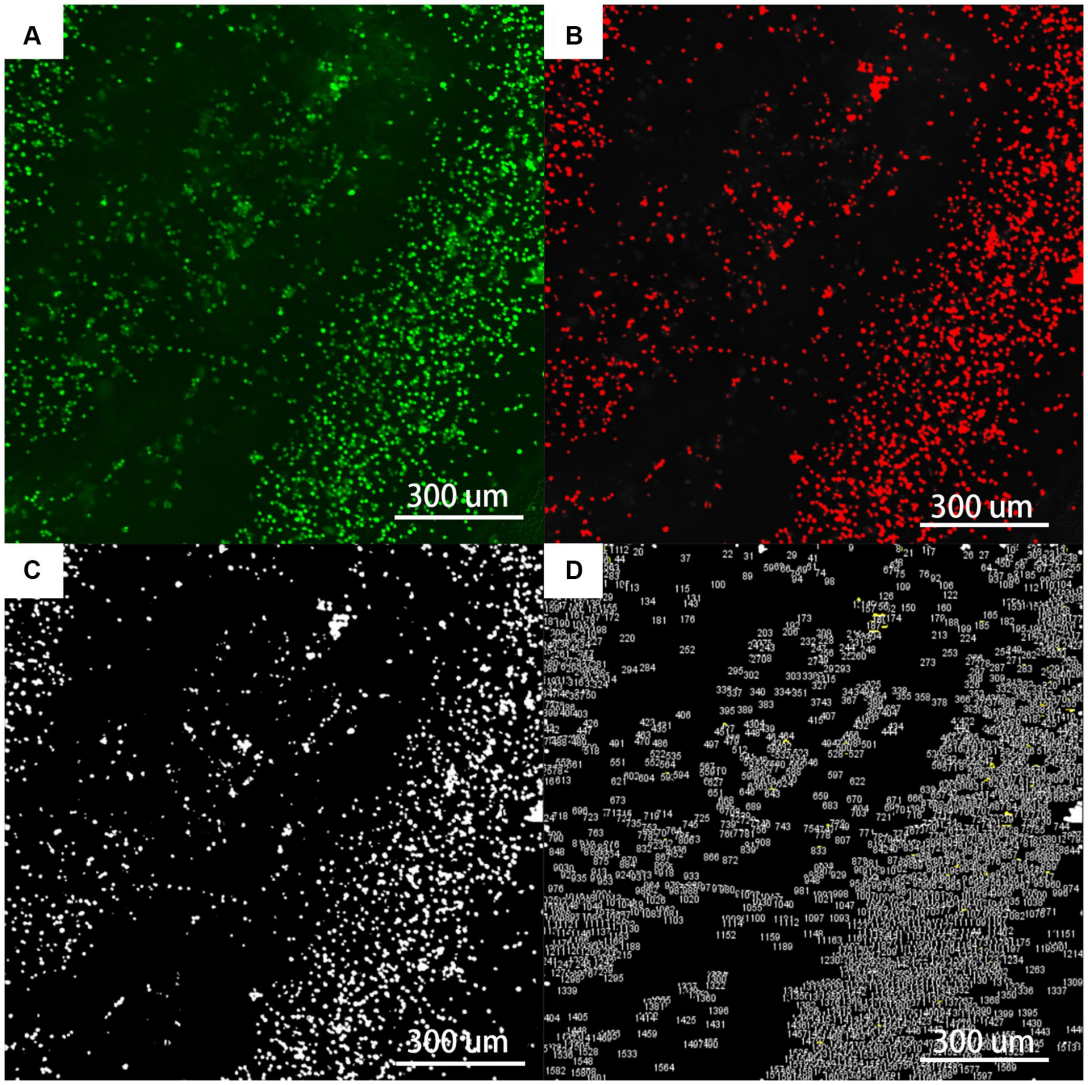
The flowchart of fluorescent staining analysis by Fiji (×400). **(A)** Is the original picture of LIVE/DEAD fluorescent staining; **(B)** is the Fiji software machine learning to segment the fibrils (red) from the background (black); **(C)** is the use of Threshold and Watershed Technology to completely separate the fibrils; **(D)** is the software to count the number and area of each ellipse.

After the tendon and BMSCs were cultured for 1–2 weeks, cell adhesion was observed under a confocal microscope. The cell adhesion rates for the control group, 7-min, 11-min, 18-min, and 30-min groups were 3.10 ± 0.56%, 4.59 ± 1.51%, 5.36 ± 1.24%, 6.12 ± 1.98%, and 8.27 ± 2.34%, respectively (*F* = 6.755, *p* = 0.001). Regarding cell growth, longer trypsin treatment duration was found to be more conducive to cell growth (Table 1 and Figures 9, 10).

### Mechanical characteristics

The average cross-sectional area of the tendon in the control group, 7-min, 11-min, 18-min, and 30-min groups were 4.94 ± 0.11 mm^2^, 5.11 ± 0.21 mm^2^, 5.13 ± 0.16 mm^2^, 4.67 ± 0.52 mm^2^, and 4.94 ± 0.22 mm^2^, respectively (H = 5.277, *p* = 0.260) (Table 2).

**Table 2:** Description and comparison of tendon biomechanical indicators for different treatments.

Group	CSA (mm^2^)	Stiffness (N/mm)	Ultimate load (N)	Max. elogation (mm)	Elastic modulus (MPa)	Max. stress (MPa)	Max. strain (%)	Energy absorption (MPa)	Cyclic creep (%)
Control	4.94 ± 0.11	61.09 ± 12.92	103.30 ± 10.51	3.95 ± 1.31	123.54 ± 25.28	21.06 ± 2.20	39.52 ± 13.14	7.22 ± 1.04	5.52 ± 1.21
7 min	5.11 ± 0.21	68.73 ± 13.13	99.59 ± 4.37	3.62 ± 0.85	134.86 ± 28.28	19.47 ± 0.94	36.26 ± 8.55	6.12 ± 1.05	4.43 ± 1.13
11 min	5.13 ± 0.16	59.91 ± 15.01	93.15 ± 12.38	4.11 ± 0.91	116.97 ± 31.24	18.12 ± 2.24	41.14 ± 9.06	5.45 ± 0.63	6.18 ± 0.89
18 min	4.67 ± 0.52	58.63 ± 6.27	90.42 ± 7.87	3.04 ± 0.87	125.87 ± 9.87	18.04 ± 2.48	30.35 ± 8.65	5.17 ± 0.91	5.16 ± 1.49
30 min	4.94 ± 0.22	53.39 ± 11.47	82.68 ± 6.89	3.44 ± 1.04	107.98 ± 23.08	16.78 ± 1.87	34.35 ± 10.41	4.04 ± 1.27	4.96 ± 1.31
Results	*H* = 5.277	*F* = 1.038	*F* = 4.125	*F* = 0.892	*F* = 0.830	*F* = 3.247	*F* = 0.892	*F* = 6.801	*F* = 1.403
	*p* = 0.260	*p* = 0.412	*p = 0.013	*p* = 0.487	*p* = 0.522	*p = 0.033	*p* = 0.487	*p = 0.001	*p* = 0.269

Statistical analysis for each index was performed using a one-way ANOVA (effect size: *F*). For mechanical indicators that did not conform to normal distribution and homogeneity of variance, the Kruskal–Wallis non-parametric test was used (effect size: *H*) as an alternative to the ANOVA in parametric testing. *p < 0.05 indicates statistical significance.

Ultimate Load, Elastic Modulus, Maximum Strain, Maximum Stress, Energy Absorption, and Stiffness: There was no significant difference in elastic modulus, stiffness, or maximum strain among the groups (*p* < 0.05). The difference in ultimate load between the groups was statistically significant (*F* = 4.125, *p* = 0.013). The post-hoc test showed that the difference between the control group (103.30 ± 10.51 N), the 18-min (90.42 ± 7.87 N), and the 30-min (82.68 ± 6.89 N) groups was statistically significant (*p* = 0.033, *p* = 0.001). However, there was no significant difference between the control group and the 7-min (99.59 ± 4.37 N) and 11-min (93.15 ± 12.38 N) groups (*p* = 0.515, *p* = 0.085).

Regarding maximum stress, the difference among the groups was statistically significant (*F* = 3.247, p = 0.033). The post-hoc test showed no significant difference between the control group (21.06 ± 2.20 MPa) and the 7-min group (19.47 ± 0.94 MPa) (*p* = 0.228). The difference in energy absorption density among the groups was statistically significant (*F* = 6.801, *p* = 0.001). The post-hoc test showed no significant difference between the control group (7.22 ± 1.04 kJ/m^2^) and the 7-min group (6.12 ± 1.05 kJ/m^2^) (*p* = 0.1). There were significant differences between the control group, the 11-min (5.45 ± 0.63 kJ/m^2^), the 18-min (5.17 ± 0.91 kJ/m^2^), and the 30-min groups (4.04 ± 1.27 kJ/m^2^) (*p* = 0.012, *p* = 0.004, *p* < 0.001).

Cyclic Creep: The cyclic creep in the control group, 7-min, 11-min, 18-min, and 30-min groups were 5.52 ± 1.21%, 4.43 ± 1.13%, 6.18 ± 0.89%, 5.16 ± 1.49%, and 4.96 ± 1.31%, respectively (*F* = 1.403, *p* = 0.269).

Ultimate load, which represents the maximum force a material can endure before breaking, is crucial in applications where structural integrity is paramount. Similarly, differences in maximum stress highlight varying levels of material resilience and the ability to withstand mechanical forces. Lower ultimate loads observed in the 18-min and 30 min groups may indicate that tendon mechanical properties are impaired. Materials with higher ultimate loads and maximum stress tolerance are more likely to withstand physiological forces and mechanical stresses over time, potentially leading to improved patient outcomes and longevity of implants.

### Trypsin exposure duration—characteristics relationship

**Figure 11:**
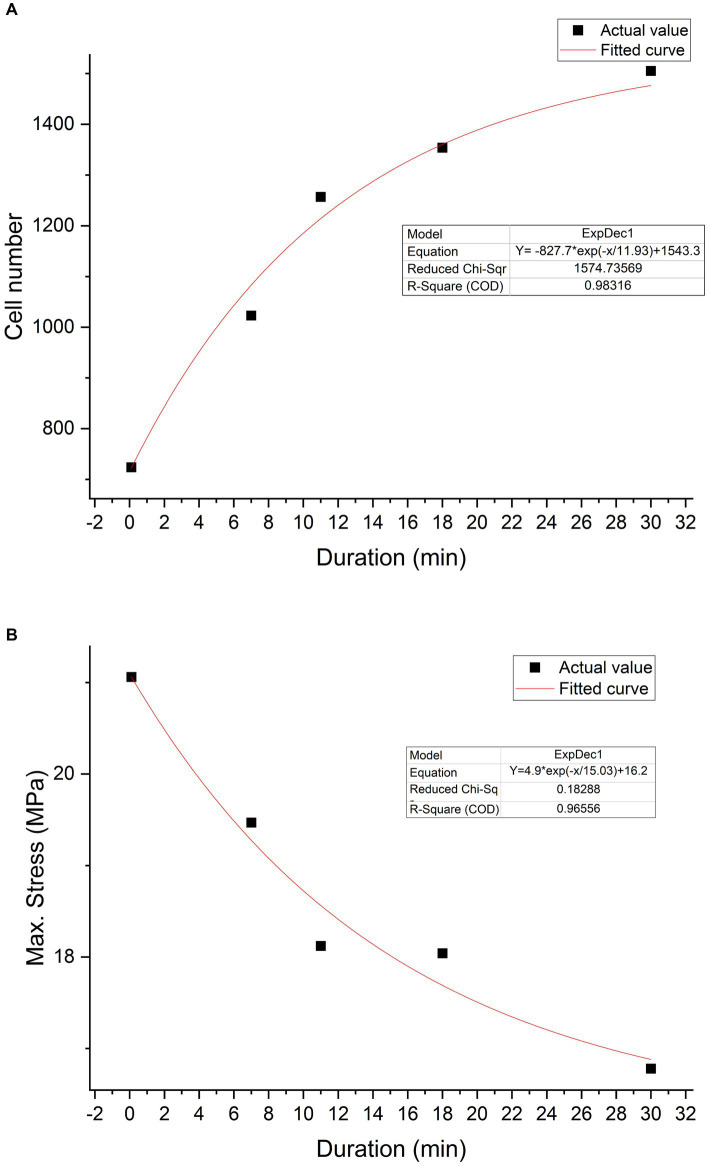
Trypsin exposure duration-characteristics relationship. Cell number—duration relationship graph **(A)**. The black squares represent actual values, while the red curve depicts the graph fitted based on a first-order exponential decay function (*Y* = −827.7*exp(−*x*/11.93) + 1,543.3). Max stress—duration relationship graph **(B)**. The black squares represent actual values, while the red curve represents the fitted curve (*Y* = 4.87e(−*x*/15.03) + 16.22).

According to the curve fitting based on cell quantity and exposure duration within the same field of view, the curve function is *Y* = −827.7*exp(−*x*/11.93) + 1,543.3, *R*^2^ = 0.98. According to the trend of the curve, it can be observed that, with increasing duration, trypsin can enhance cell adhesion. A curve fitting based on the relationship between max stress and duration was constructed using tendon samples collected at 6 weeks post-surgery. The function is *Y* = 4.87e(−*x*/15.03) + 16.22, with *R*^2^ = 0.97. It can be inferred from the curve trend that, with increasing trypsin treatment duration, the mechanical properties of the tendon decrease (Figure 11).

## Discussion

When tendons need to integrate into the bone for healing, the tendon-bone interface presents one of the most challenging clinical issues. Delayed healing may lead to serious complications such as re-tearing or decreased stability (21, 22). Notably, our findings highlight the critical role of decellularization with trypsin in optimizing tendon surfaces. This process not only removes cellular components but also enhances surface roughness, facilitating improved cell adhesion—an essential factor for effective integration into the bone. Importantly, the reduction of lubricin, GAG, and proteins during decellularization offers additional benefits for post-transplantation tendon healing. These insights are pivotal for clinical practice, as they suggest that trypsin-induced modifications may accelerate tendon-bone integration, potentially reducing the healing time and enhancing the overall outcomes in tendon repair surgeries.

In the histological examination using HE staining, our control group exhibited a compact and orderly tendon structure with visibly intact nuclei. The tendon’s condition showed a clear dependence on duration, with complete structural deterioration observed at 30 min. These histological findings align with Lohan et al.’s study, which reported cracks and loose bundles of collagen fibers following trypsin decellularization (23). In the 7-min group, the tendon structure remained largely unchanged, characterized by longitudinally arranged wavy collagen fiber bundles, albeit with the formation of some fissures. These fissures may facilitate cell infiltration and distribution during and after reseeding. Previous studies on tendon decellularization have similarly noted structural losses in decellularized ECM, which is likely influenced by biochemical changes induced by reagent components (24). However, it has been demonstrated that the main collagen structure is preserved in the tendon tissue decellularized using a similar protocol (25).

Scanning electron microscope (SEM) clearly revealed the arrangement of tendon collagen fibers, gaps, and fiber curl waves (26). We observed that the collagen fibers in the control group were neatly arranged with a smooth surface. With an increase in trypsin treatment duration, the tendon surface became rougher, and voids were clearly visible in the 30-min group. This reduction in collagen bundle integrity is attributed to the reduction of salt- and acid-soluble collagen caused by trypsin, which subsequently deforms the collagen bundles (27). Franchi et al. treated rat Achilles tendons with chondroitinase, and intermittent curly collagen fibers of different sizes were observed under SEM, similar to our study results (28). The damage to tendon ultrastructure is associated with the macroscopic mechanical properties, which are reduced after trypsin treatment (29).

The GAG-stained blue could be clearly observed in the AB staining in the control group. We quantified the GAG content using OD and found a negative correlation with the duration of trypsin action. It is well known that natural ECM loses some GAG during decellularization (30), while trypsin disrupts the ECM ultrastructure and removes collagen, elastin, and GAGs at a higher rate than detergents such as sodium dodecyl sulfate (SDS) (31).

In the biochemical analysis, our study revealed that the lubricin, GAG, and total protein contents were highest in the control group and lowest in the-30 min group, with these contents negatively correlated with trypsin treatment duration. Lubricin, the product of the proteoglycan 4 (PRG4) gene, is an O-linked glycosylated protein highly expressed in synoviocytes (16). It plays a crucial cytoprotective role by preventing cell adhesion to the tendon surface and providing lubrication during normal tendon function (32). However, excessive lubricin can inhibit tendon repair and prolong healing duration (11, 19). Trypsin treatment reduces lubricin, leading to partial digestion of matrix adhesion molecules, which can be beneficial for the healing process (27, 33). Previous studies have shown that trypsin reduces lubricin, GAG, and total protein content (31), which is consistent with our findings.

GAG possesses a high degree of polymerization and macromolecular structure. Apart from hyaluronic acid, other GAG members undergo sulfation or other modifications and attach themselves to core proteins (34). The decreased GAG and collagen contents also suggest a weakened stromal immune response, while lower GAG amounts may promote cell adhesion (35). Crapo et al. demonstrated that ECM proteins have limited resistance to trypsin cleavage. Hence, caution should be exercised when using tissues exposed to trypsin (7). Gilpin et al. also demonstrated that longer treatment durations lead to a decrease in GAG, collagen, and elastin content, resulting in reduced mechanical strength (33). Therefore, we should also consider the effect of excessively long processing duration on the mechanical properties of the tendon.

Using confocal microscopy, we evaluated cell adhesion rates among the groups by measuring the average percentage of cells covering the tendon surface. We observed that the adhesion rate was lowest in the control group and highest in the 30-min group. Our findings suggest that trypsin digestion enhances BMSCs’ coverage on the tendon surface, consistent with the findings reported by Hashimoto et al. (11). The complete removal of cells and residues through chemical decellularization creates additional space, potentially facilitating the natural homing of cells *in vivo* for further differentiation (36).

Decellularized scaffolds offer more surface area, aiding in rapid cell attachment to the ECM (8). SEM revealed that the tendons became rougher after trypsin treatment, providing a more available surface for cell attachment compared to smooth surfaces. Additionally, the roughness could be attributed to changes in the content of the tendon matrix or chemical stimulation resulting from alterations to tendon surface collagen, lubricin, and related matrix macromolecules (11). Surface chemistry appears to play a significant role in cellular behavior. Lubricin, GAG, and collagen on the tendon surface can influence cellular behavior. Our results suggest that trypsin treatment alters the molecular environment of the tendon surface, leading to an increased coverage of the tendon surface by BMSCs.

Regarding the mechanical results, the ultimate load, maximum stress, and energy absorption of the tendon were reduced compared to the control group, and they showed a downward trend with increased enzyme treatment duration. This finding indicates that tendons, after trypsin decellularization, exhibited poorer mechanical properties. However, the decellularized scaffold and its poor mechanical properties may limit clinical application (37, 38). Collagen fibrils in tendons are believed to be discontinuous, transmitting tensile loads through shear forces generated during fibril sliding (39).

Cells can acutely adapt to mechanical forces applied to them and transmit forces through the ECM without any loss of mechanical power (40). Various extrafibrillar tissue components are believed to transmit interfibrillar loads by bridging collagen fibers (41).

Trypsin digestion successfully removed a large number of extrafibrillar proteins, including GAG, tenascin C, and collagen XII, which weakened the transmission of tensile loads between the fibers, thereby reducing mechanical properties. Additionally, trypsin directly damages the fibers, causing them to break and fracture, further contributing to decreased mechanical strength.

From the results, it is evident that there is a negative correlation between treatment duration and mechanical strength. Preserving mechanical performance is crucial for the subsequent healing and recovery of allograft tendons. Considering the impact of other factors, we aim to minimize trypsin exposure duration as much as possible.

Upon analysis, it becomes apparent that, during decellularization with trypsin, not only does the tendon undergo the removal of cellular components but its surface also undergoes optimization, resulting in increased roughness that fosters enhanced cell adhesion. Moreover, the removal of lubricin, GAG, and proteins undoubtedly confers additional benefits to the post-transplantation healing process of the tendon.

Observations made under morphological examination in our study exhibit a discernible dependency on duration. As the duration of processing extends, there is a proportional decrease in cellular constituents within collagen fibers, coupled with a notable increase in collagen fiber damage, which is particularly evident in the 18-min and 30-min groups.

Even though cell adhesion rates peak at 30 min, due consideration must be given to mechanical performance. Notably, with longer processing durations, there is a conspicuous reduction in the tendon’s ultimate load and maximum stress.

In summary, we contend that trypsin exposure to tendons manifests as a dual-factor influence, simultaneously promoting cell adhesion and adversely affecting mechanical strength. Therefore, it is crucial to balance the benefits of enhanced cell adhesion with the potential drawbacks of reduced mechanical integrity when determining the optimal duration of trypsin treatment.

However, we acknowledge that our study has some limitations. First, there are inherent differences between the animal tendons used in our experiment and human tendons, with the latter potentially providing more valuable data for guiding allogeneic transplantation. Second, we lacked imaging assessments, such as MRI or X-ray, to evaluate tendon and bone healing. Third, our study has shortcomings in molecular mechanism experiments. Therefore, future studies should research molecular matrix aspects to evaluate changes in allogeneic tendon grafts during the reconstruction process.

To bridge the gap between animal models and human clinical applications, future studies should consider the following detailed approaches:

1. **Humanized Animal Models**: This approach involves developing methodologies to better replicate human tendon conditions in animal models and integrating translational research that directly guides clinical practice.
2. **Translational Biomaterials Research**: This approach involves exploring advanced biomaterials that can better simulate the extracellular matrix environment of human tendons and developing scaffolds that mimic the biochemical and mechanical properties of native human tendon tissue, thereby improving graft integration and functional outcomes in transplantation.
3. **Clinical Validation Studies**: This approach involves initiating clinical validation studies to assess graft integration, biomechanical performance, and patient outcomes in order to validate the efficacy and safety of these approaches in clinical practice.

By addressing these aspects, future research can overcome current limitations and accelerate the translation of experimental findings.

## Conclusion

In conclusion, trypsin treatment of tendons not only facilitates decellularization but also influences cell adhesion through alterations in collagen fiber morphology and ECM substance content, thereby providing a theoretical basis for enhancing *in vivo* healing. Our findings establish a duration-dependent relationship where longer trypsin exposure correlates positively with adhesion strength but negatively affects mechanical strength. Therefore, when determining the duration of trypsin treatment, careful consideration of these dual effects is crucial for balancing the benefits and potential drawbacks.

## Data Availability

The original contributions presented in the study are included in the article/Supplementary material, further inquiries can be directed to the corresponding authors.
