# Supplementary material for: Is there a duration-characteristic relationship for trypsin exposure on tendon? A study on anterior cruciate ligament reconstruction in a rabbit model
Source: Front Med (Lausanne). 2024 Aug 21;11:1417930. doi: 10.3389/fmed.2024.1417930 (PMC11371708; doi:10.3389/fmed.2024.1417930)
Supplement: Supplementary file 1 [file Data_Sheet_1.zip › Biochemical Analysis/Detailed content values.docx]

| Total protein （Group） | Value (ug/mL) |  | Lubricin Concentration  （Group） | Value  （ng/L） |  | GAG content（Group） | Value  (ug/ml) |
| --- | --- | --- | --- | --- | --- | --- | --- |
| A1 | 2445.43 |  | A1 | 838.125 |  | A1 | 129.95 |
| A2 | 2732.51 |  | A2 | 807.5 |  | A2 | 160.65 |
| A3 | 2782.61 |  | A3 | 718.125 |  | A3 | 178.64 |
| A4 | 2841.79 |  | A4 | 802.13 |  | A4 | 138.86 |
| A5 | 2474.01 |  | A5 | 804.06 |  | A5 | 150.41 |
| B1 | 2548.29 |  | B1 | 670.625 |  | B1 | 115.81 |
| B2 | 2594.04 |  | B2 | 801.875 |  | B2 | 98.53 |
| B3 | 2401.64 |  | B3 | 602.5 |  | B3 | 141.86 |
| B4 | 2248.27 |  | B4 | 711.93 |  | B4 | 122.62 |
| B5 | 2201.34 |  | B5 | 661.49 |  | B5 | 118.26 |
| C1 | 2451.67 |  | C1 | 762.5 |  | C1 | 89.52 |
| C2 | 2391.13 |  | C2 | 591.25 |  | C2 | 108.05 |
| C3 | 2263.07 |  | C3 | 672.5 |  | C3 | 77.4 |
| C4 | 2193.84 |  | C4 | 695.46 |  | C4 | 101.2 |
| C5 | 2161.30 |  | C5 | 654.38 |  | C5 | 78.47 |
| D1 | 2305.91 |  | D1 | 709.375 |  | D1 | 76.81 |
| D2 | 2006.52 |  | D2 | 680 |  | D2 | 90.12 |
| D3 | 2163.49 |  | D3 | 537.5 |  | D3 | 68.11 |
| D4 | 2319.18 |  | D4 | 657.43 |  | D4 | 86.92 |
| D5 | 2101.64 |  | D5 | 631.49 |  | D5 | 77.13 |
| E1 | 1781.43 |  | E1 | 611.25 |  | E1 | 66.86 |
| E2 | 2053.27 |  | E2 | 479.375 |  | E2 | 75.52 |
| E3 | 1894.76 |  | E3 | 610 |  | E3 | 70.22 |
| E4 | 1740.92 |  | E4 | 574.31 |  | E4 | 82.32 |
| E5 | 1990.84 |  | E5 | 555.24 |  | E5 | 73.16 |

1mL=1000μL

0.31μg/μL=0.31ug/0.001ml=0.31/0.001*ug/ml=310 ug/ml

| Total protein | |  | Lubricin Concentration | |  | GAG content |  |
| --- | --- | --- | --- | --- | --- | --- | --- |
| control | a |  | control | a |  | control | a |
| 7min | b |  | 7min | b |  | 7min | b |
| 11min | b |  | 11min | b |  | 11min | c |
| 18min | b |  | 18min | bc |  | 18min | c |
| 30min | c |  | 30min | c |  | 30min | c |

| 30min | 5 | 1892.2440 |  |  |
| --- | --- | --- | --- | --- |
| 18min | 5 |  | 2160.9480 |  |
| 11min | 5 |  | 2292.2020 |  |
| 7min | 5 |  | 2398.7160 |  |
| control | 5 |  |  | 2655.2700 |
|  |  | c | b | a |
