# Supplementary material for: Is there a duration-characteristic relationship for trypsin exposure on tendon? A study on anterior cruciate ligament reconstruction in a rabbit model
Source: Front Med (Lausanne). 2024 Aug 21;11:1417930. doi: 10.3389/fmed.2024.1417930 (PMC11371708; doi:10.3389/fmed.2024.1417930)

| Group |  | Cell number | Area ratio（%）  Adhesion rate |  |
| --- | --- | --- | --- | --- |
| **Control** | A1 | 875 | 3.48 |  |
| **Control** | A2 | 586 | 2.83 |  |
| **Control** | A3 | 720 | 3.06 |  |
| **Control** | A4 | 550 | 2.34 |  |
| **Control** | A5 | 890 | 3.79 |  |
| **7 min** | B1 | 987 | 2.85 |  |
| **7 min** | B2 | 1120 | 6.77 |  |
| **7 min** | B3 | 1005 | 4.47 |  |
| **7 min** | B4 | 820 | 3.64 |  |
| **7 min** | B5 | 1184 | 5.26 |  |
| **11 min** | C1 | 1196 | 7.17 |  |
| **11 min** | C2 | 911 | 3.76 |  |
| **11 min** | C3 | 1485 | 5.65 |  |
| **11 min** | C4 | 1421 | 5.40 |  |
| **11 min** | C5 | 1274 | 4.84 |  |
| **18 min** | D1 | 1605 | 7.50 |  |
| **18 min** | D2 | 1144 | 3.02 |  |
| **18 min** | D3 | 1374 | 6.87 |  |
| **18 min** | D4 | 1580 | 7.90 |  |
| **18 min** | D5 | 1070 | 5.35 |  |
| **30 min** | E1 | 1801 | 10.74 |  |
| **30 min** | E2 | 1008 | 4.42 |  |
| **30 min** | E3 | 1563 | 8.68 |  |
| **30 min** | E4 | 1653 | 9.18 |  |
| **30 min** | E5 | 1504 | 8.36 |  |

Original scale length: 40px-100 200px-300um

Scale numbers: 48 Capital

letters: 80

**Control**

=
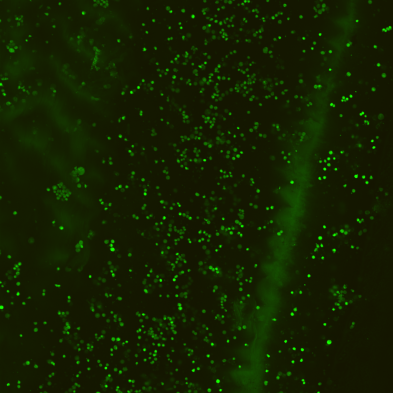


**7 min**


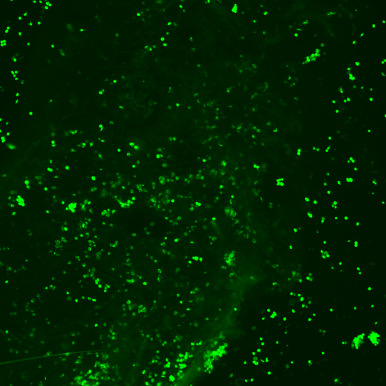


**11 min**


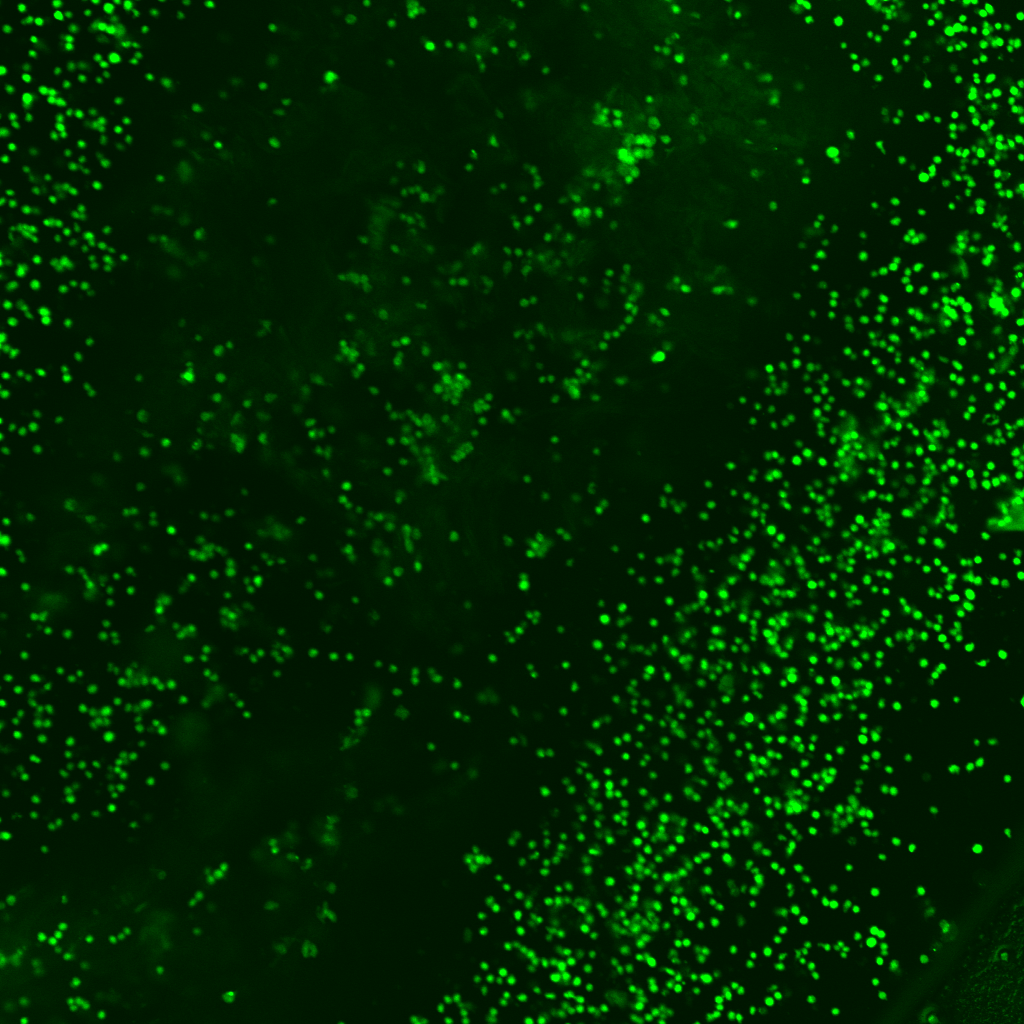


**18 min**


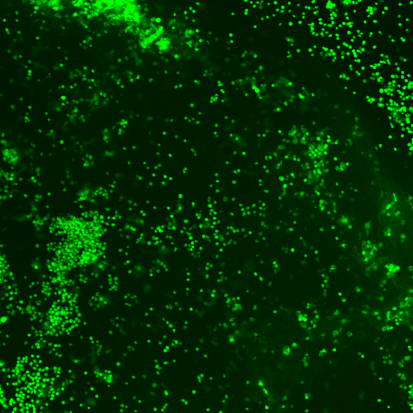


**30 min**


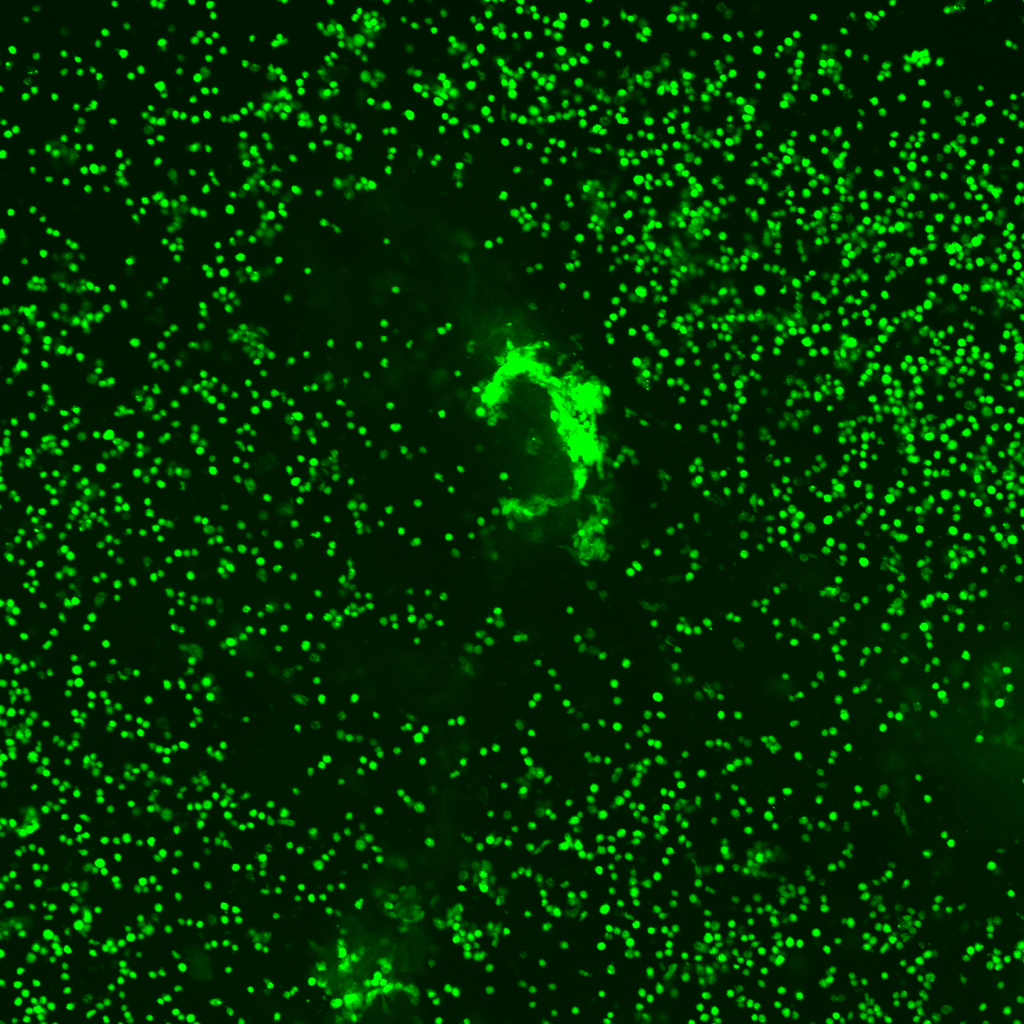


Procedure：


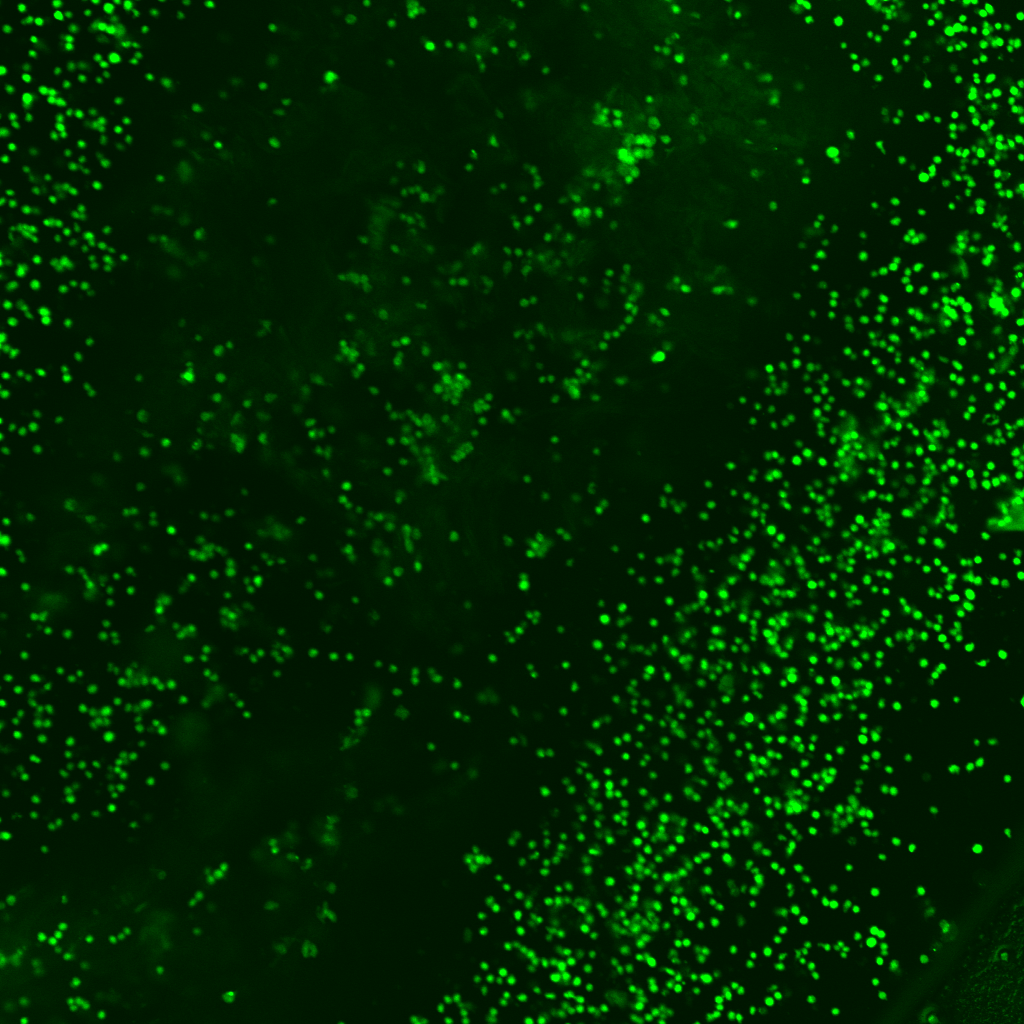

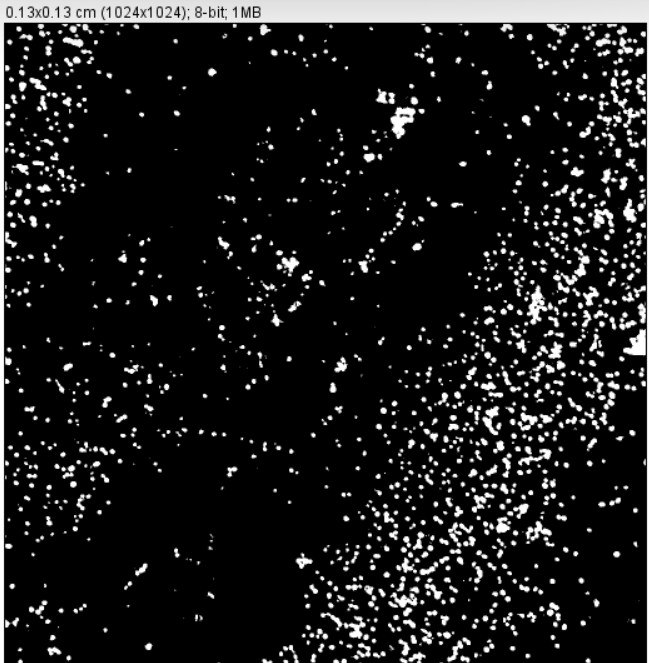

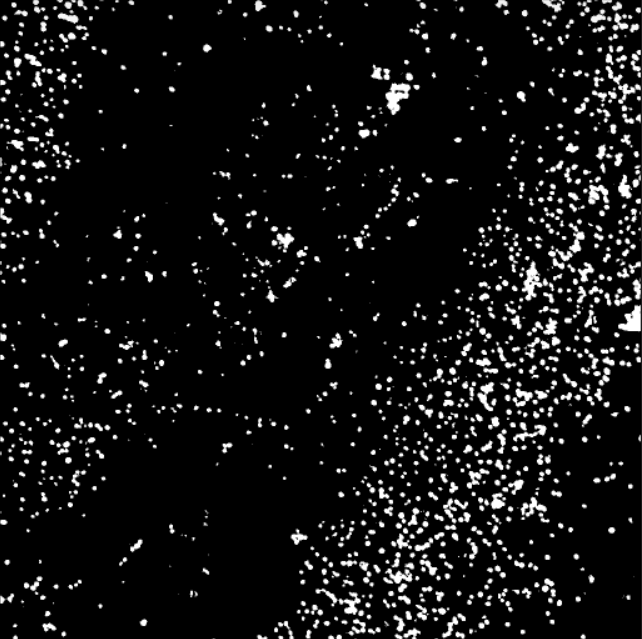

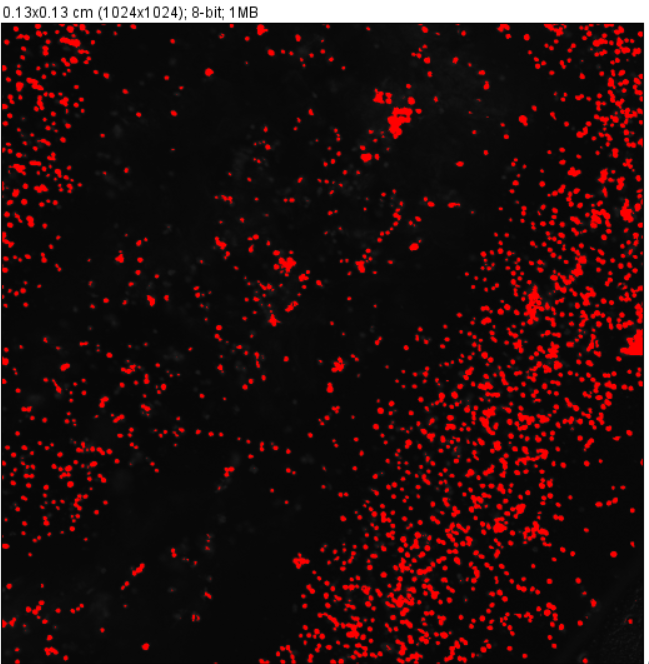

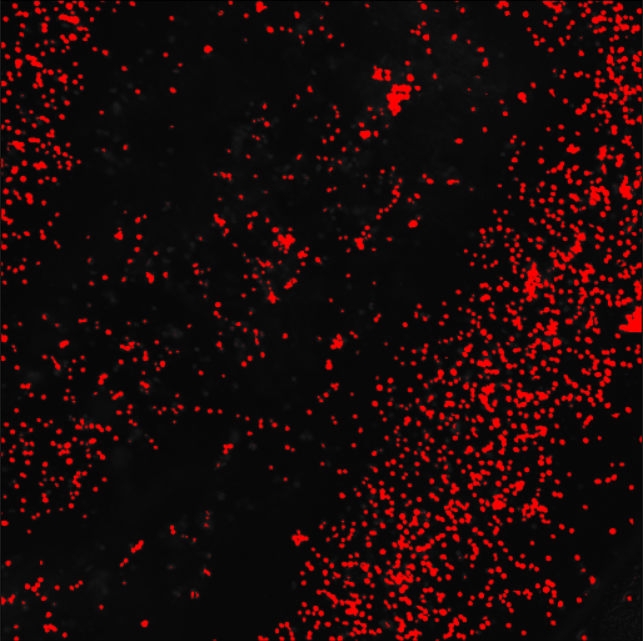

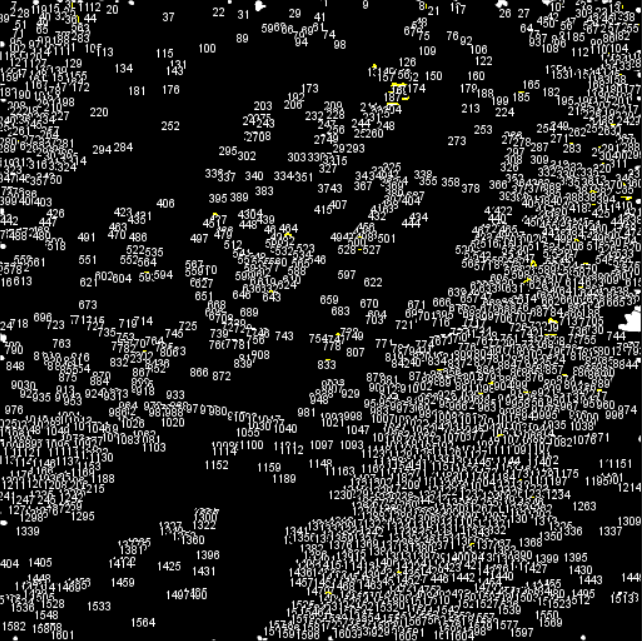

Supplement: Supplementary file 1 [file Data_Sheet_1.zip › Tenocyte number, Adhesion rate (Table 1)/Staining results and counting process.docx]
