# Supplementary figures and images for: Is there a duration-characteristic relationship for trypsin exposure on tendon? A study on anterior cruciate ligament reconstruction in a rabbit model
Source: Front Med (Lausanne). 2024 Aug 21;11:1417930. doi: 10.3389/fmed.2024.1417930 (PMC11371708; doi:10.3389/fmed.2024.1417930)

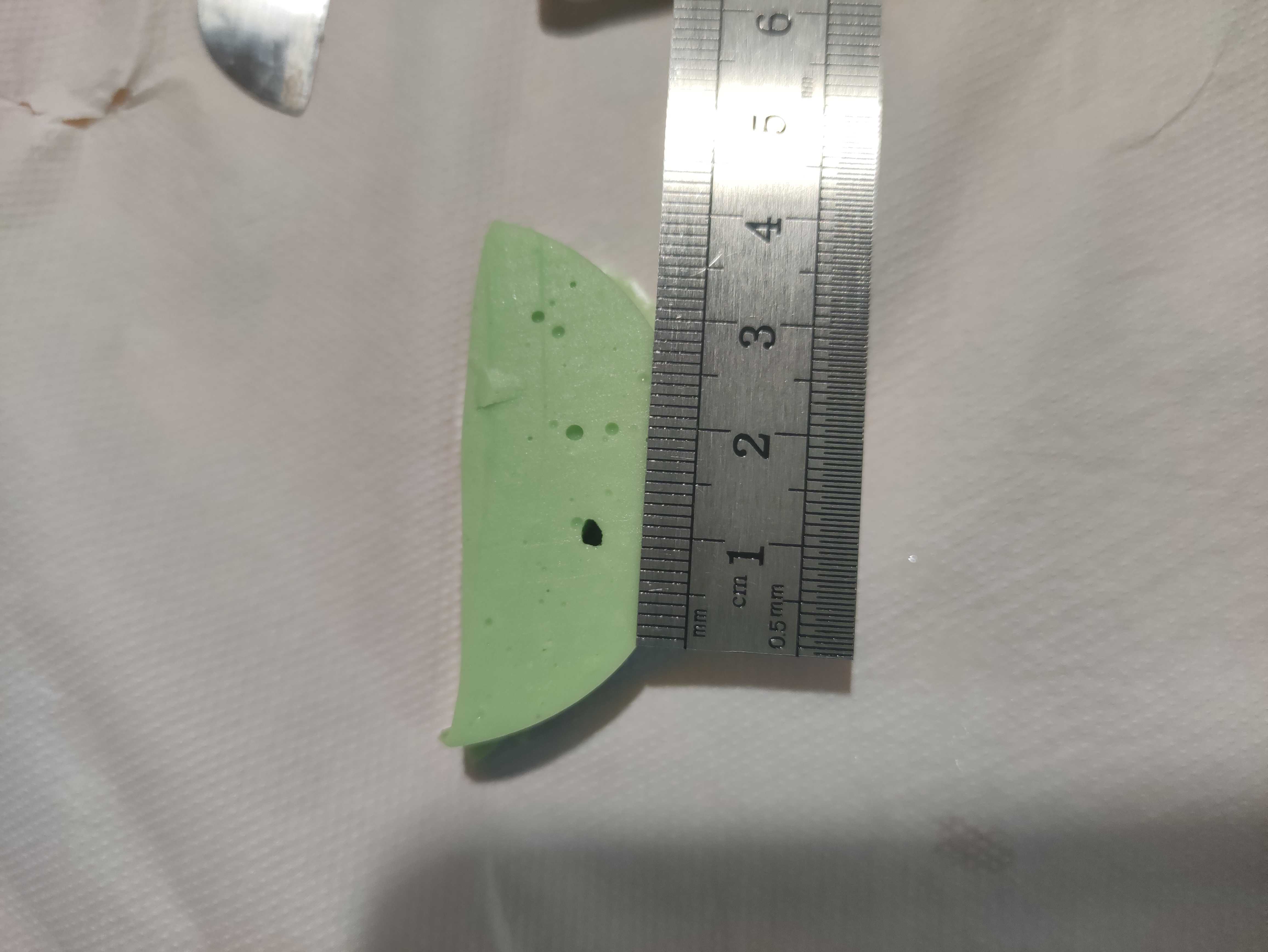

Supplement: Supplementary file 1 [file Data_Sheet_1.zip › Mechanical Characteristics (Table 2)/Cross-sectional Area (Alginate Impression)/A1-1(1).jpg]

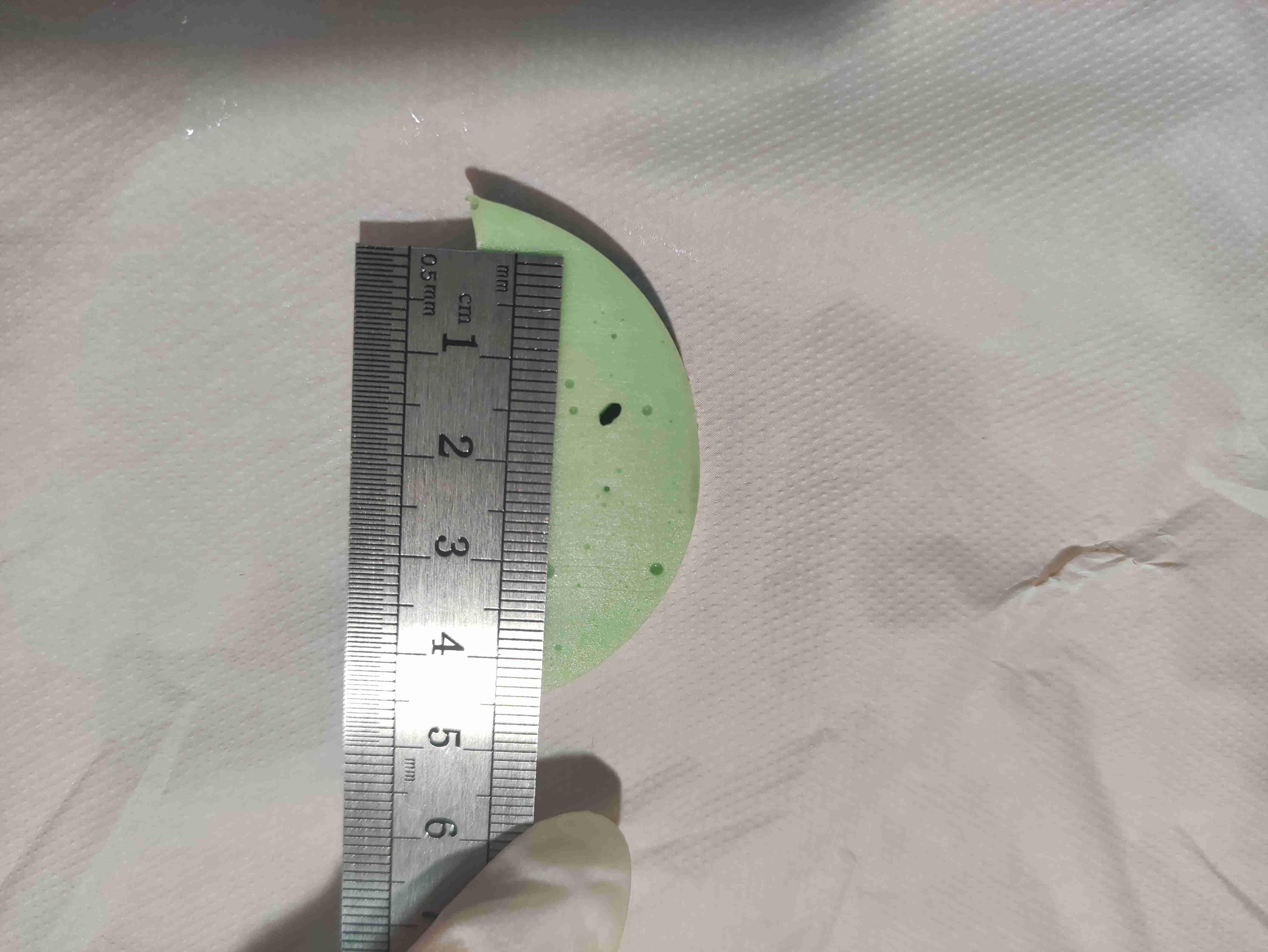

Supplement: Supplementary file 1 [file Data_Sheet_1.zip › Mechanical Characteristics (Table 2)/Cross-sectional Area (Alginate Impression)/A1-3(1).jpg]

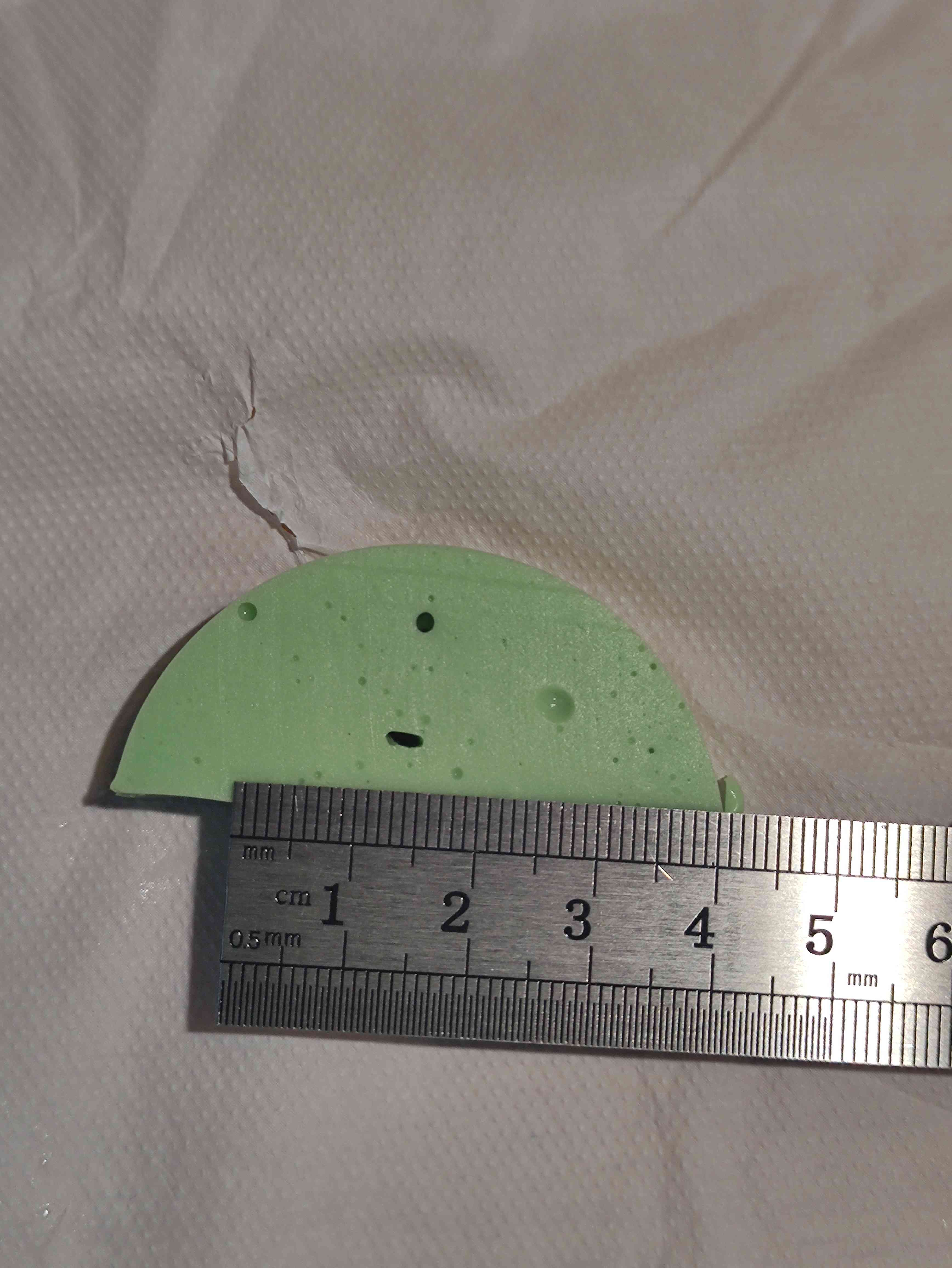

Supplement: Supplementary file 1 [file Data_Sheet_1.zip › Mechanical Characteristics (Table 2)/Cross-sectional Area (Alginate Impression)/A2-1(1).jpg]

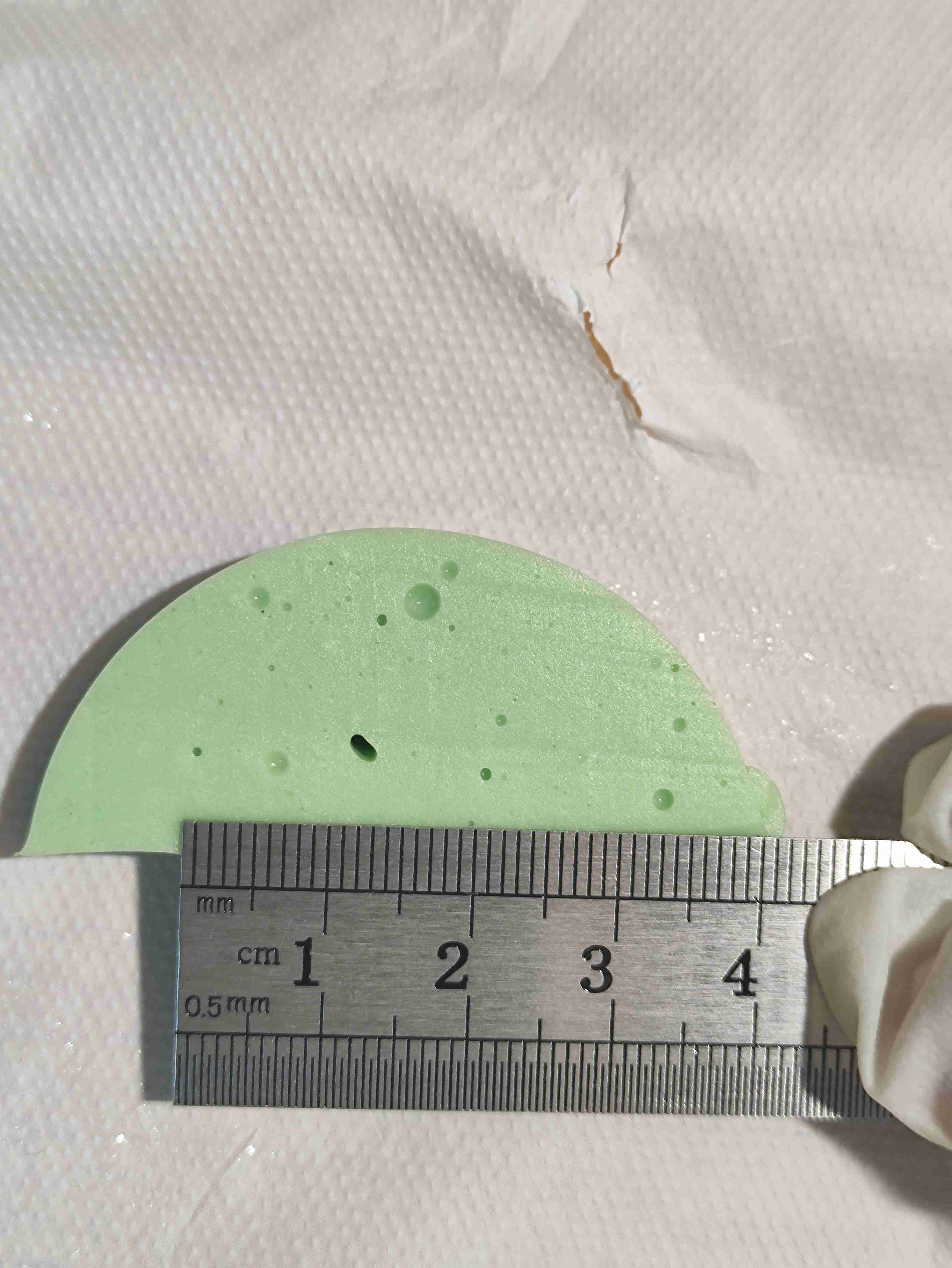

Supplement: Supplementary file 1 [file Data_Sheet_1.zip › Mechanical Characteristics (Table 2)/Cross-sectional Area (Alginate Impression)/A2-2(1).jpg]

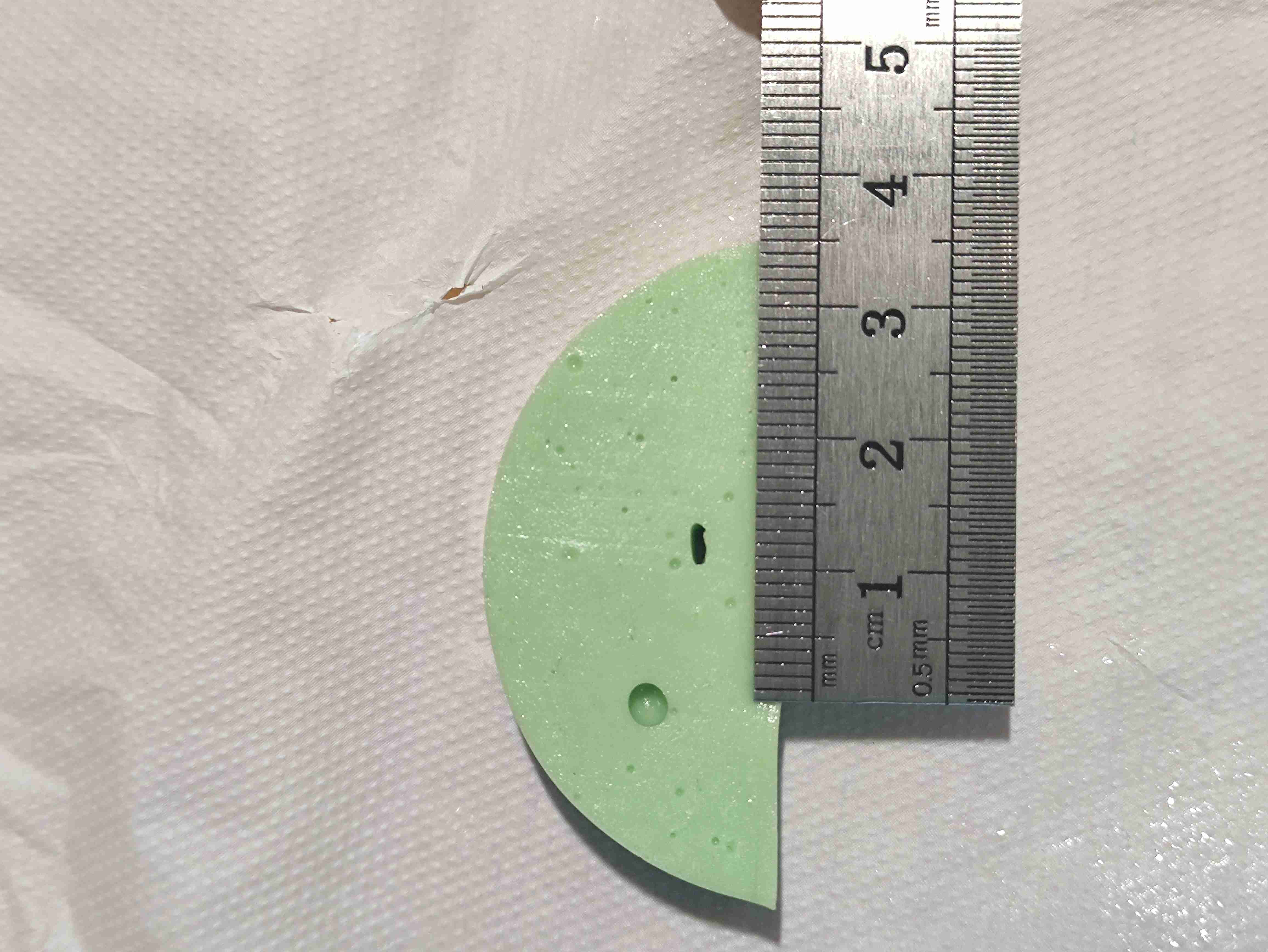

Supplement: Supplementary file 1 [file Data_Sheet_1.zip › Mechanical Characteristics (Table 2)/Cross-sectional Area (Alginate Impression)/A2-3(1).jpg]

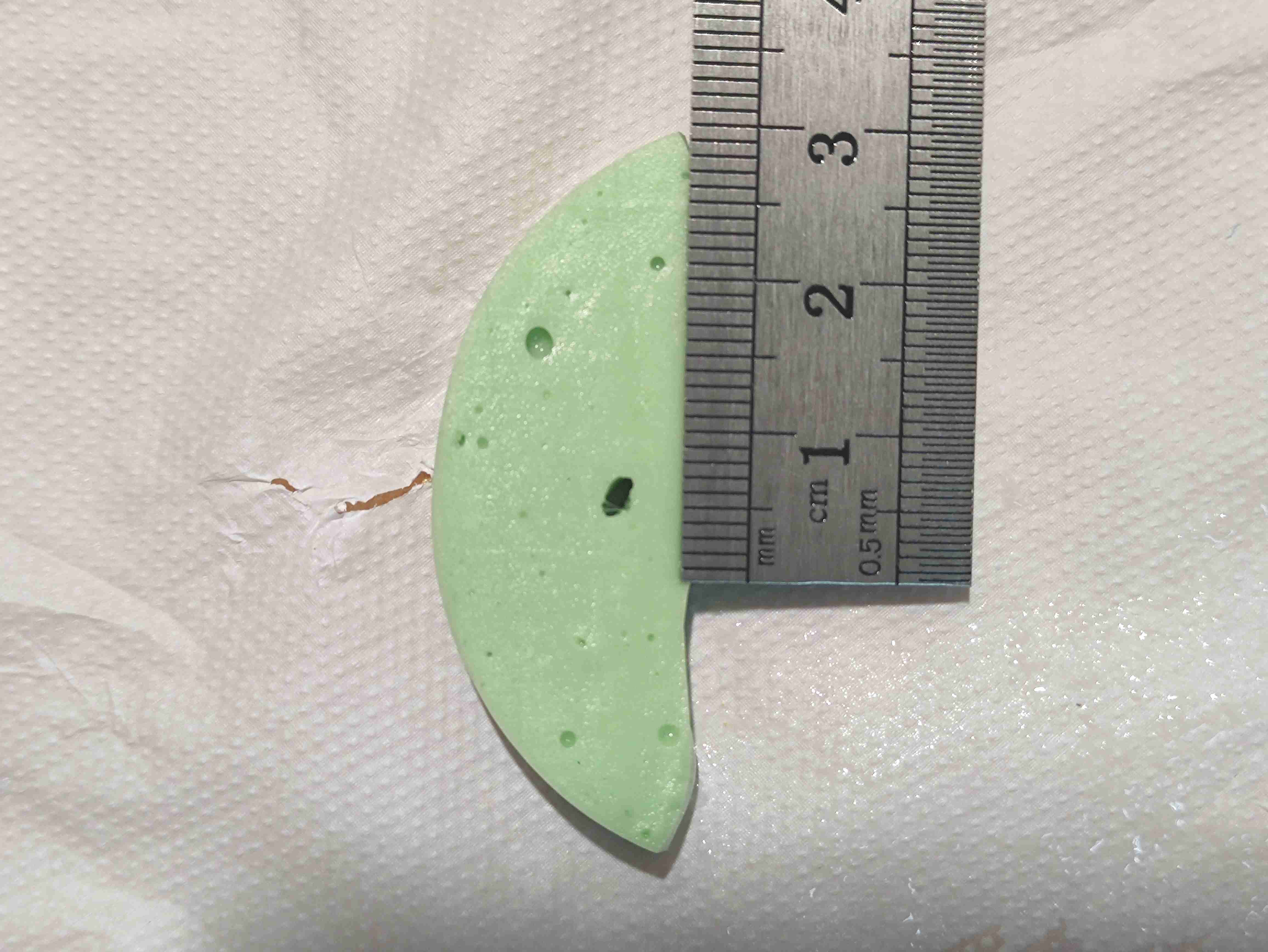

Supplement: Supplementary file 1 [file Data_Sheet_1.zip › Mechanical Characteristics (Table 2)/Cross-sectional Area (Alginate Impression)/B1-1(1).jpg]

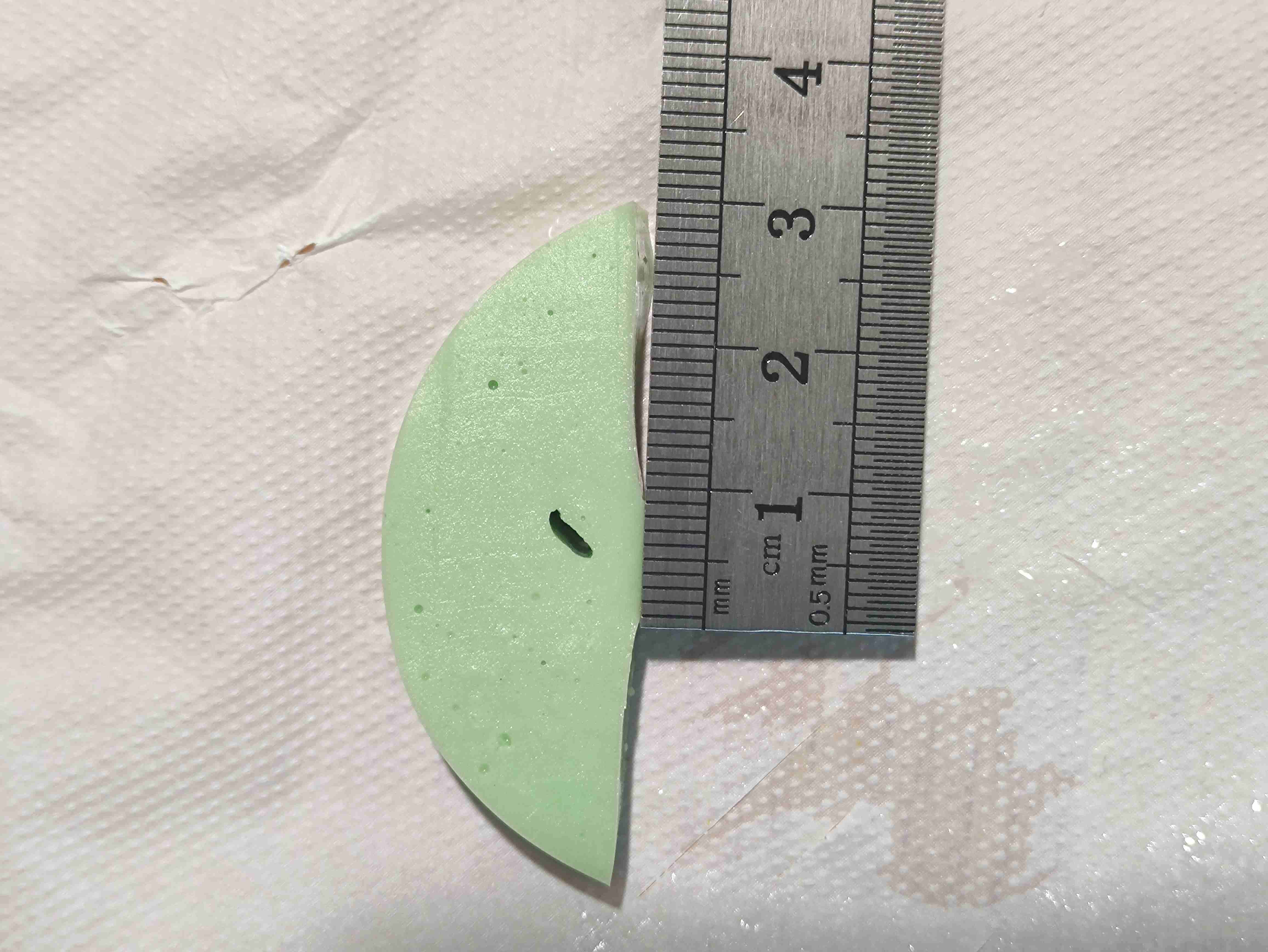

Supplement: Supplementary file 1 [file Data_Sheet_1.zip › Mechanical Characteristics (Table 2)/Cross-sectional Area (Alginate Impression)/B1-2(1).jpg]

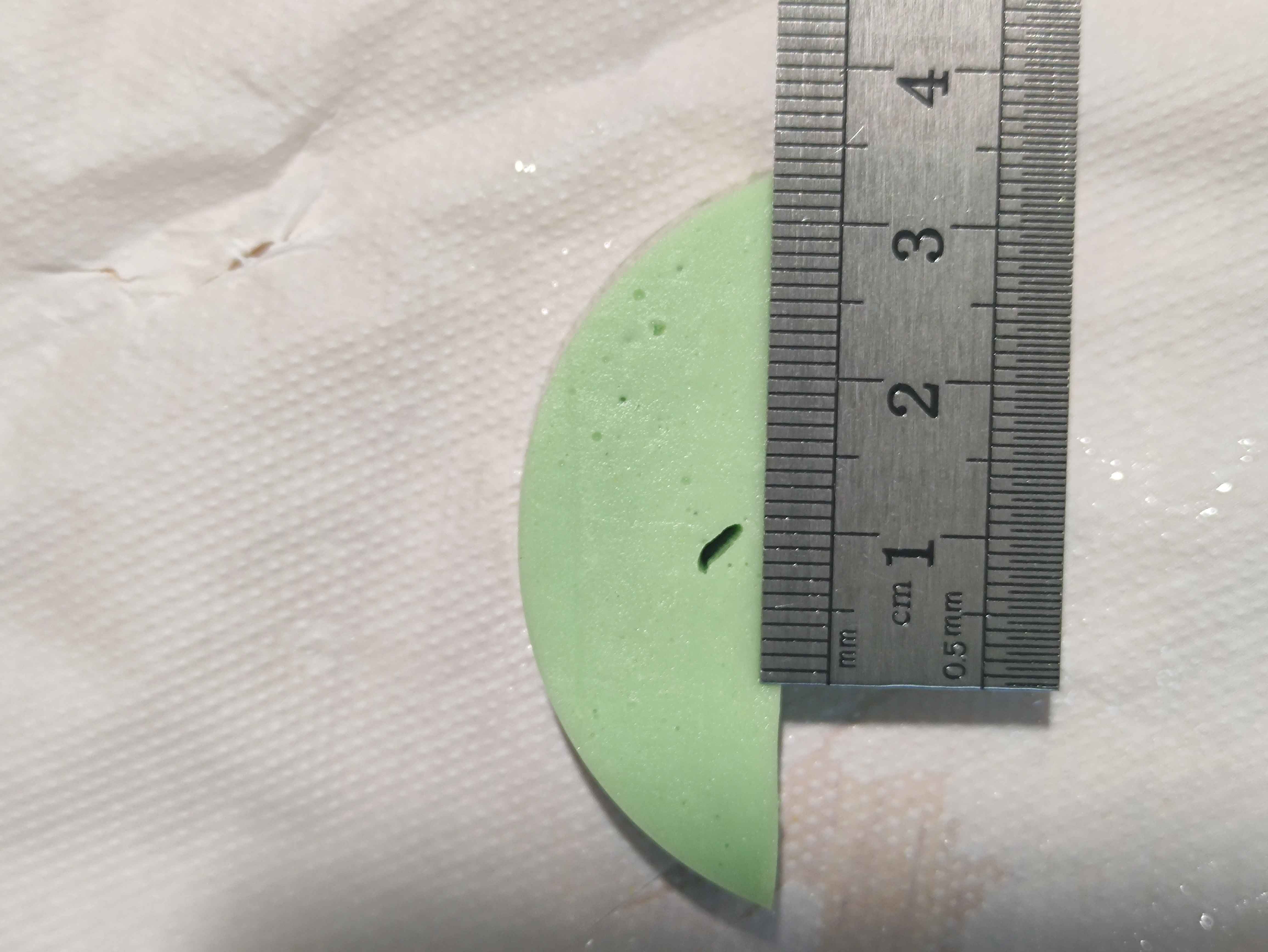

Supplement: Supplementary file 1 [file Data_Sheet_1.zip › Mechanical Characteristics (Table 2)/Cross-sectional Area (Alginate Impression)/B1-3(1).jpg]

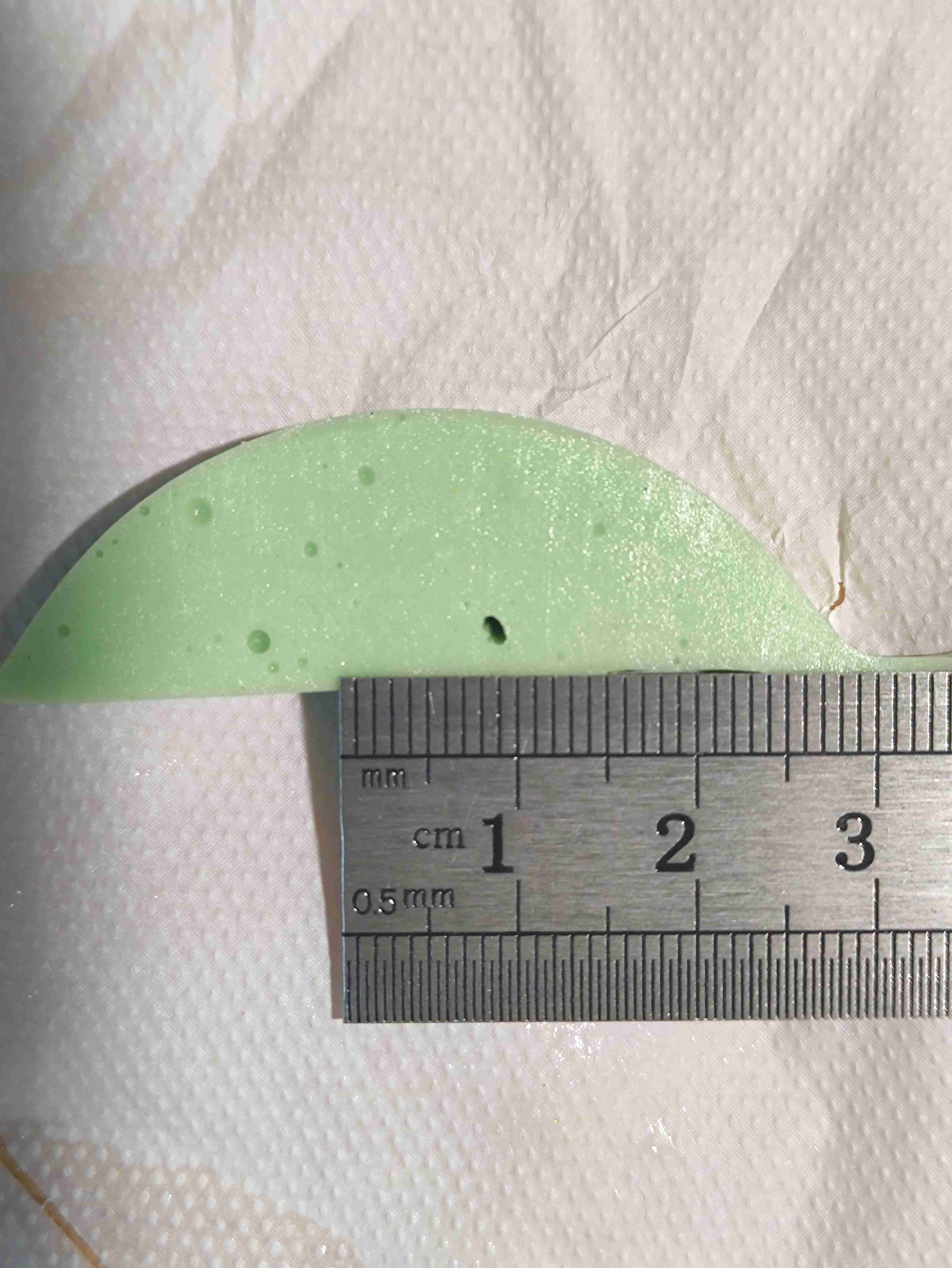

Supplement: Supplementary file 1 [file Data_Sheet_1.zip › Mechanical Characteristics (Table 2)/Cross-sectional Area (Alginate Impression)/C1-1(1).jpg]

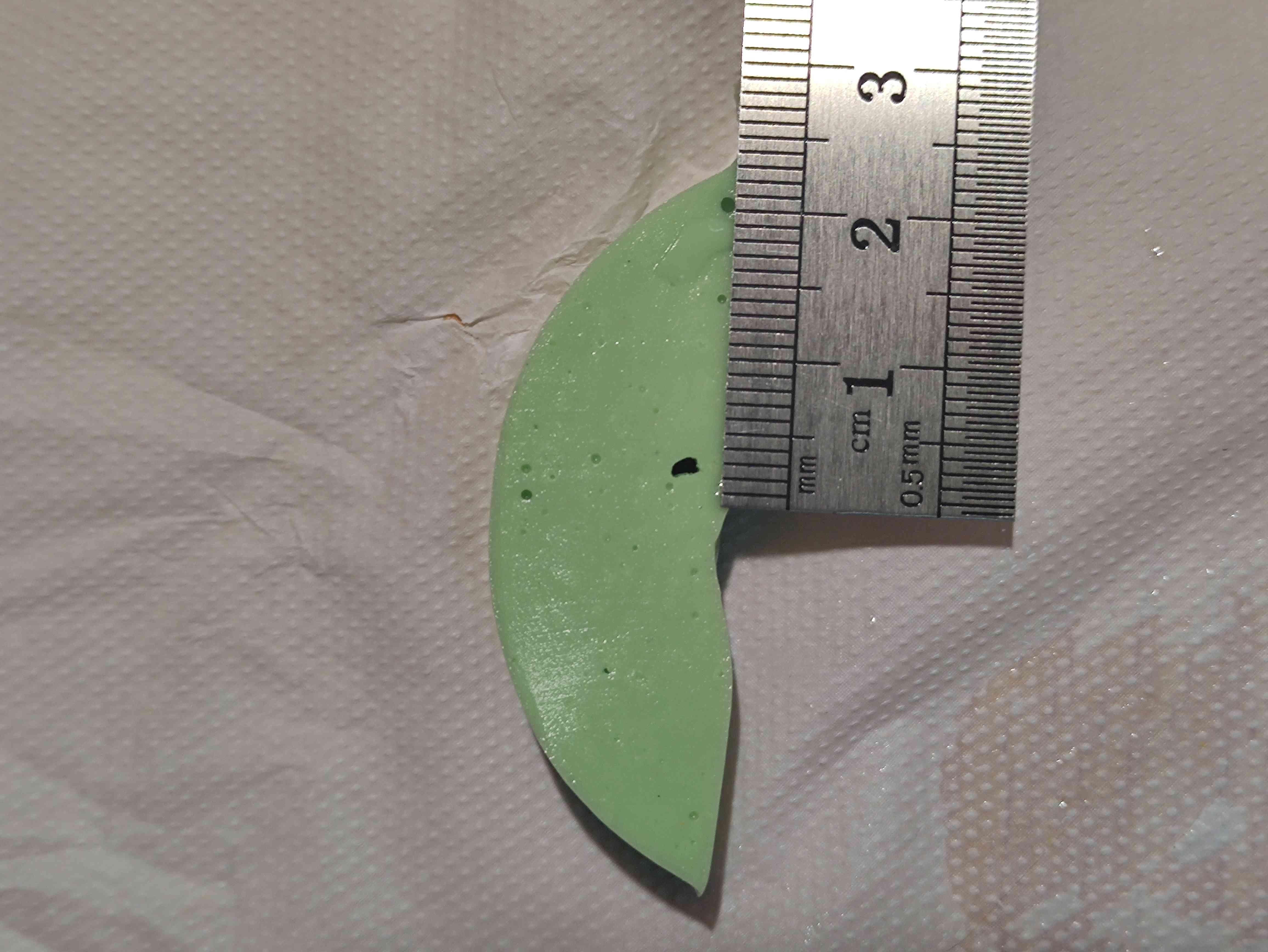

Supplement: Supplementary file 1 [file Data_Sheet_1.zip › Mechanical Characteristics (Table 2)/Cross-sectional Area (Alginate Impression)/C1-2(1).jpg]

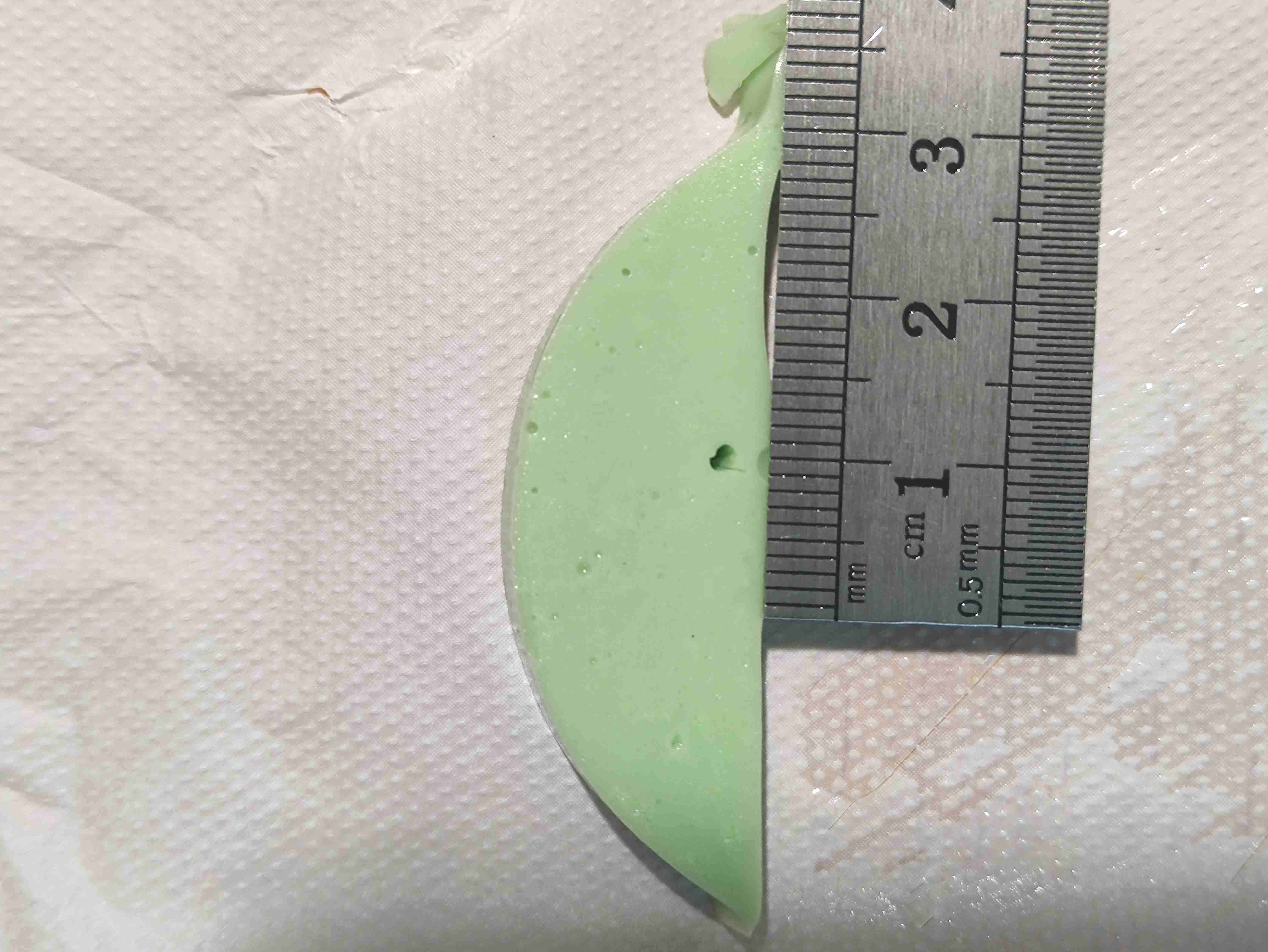

Supplement: Supplementary file 1 [file Data_Sheet_1.zip › Mechanical Characteristics (Table 2)/Cross-sectional Area (Alginate Impression)/C1-3.jpg]

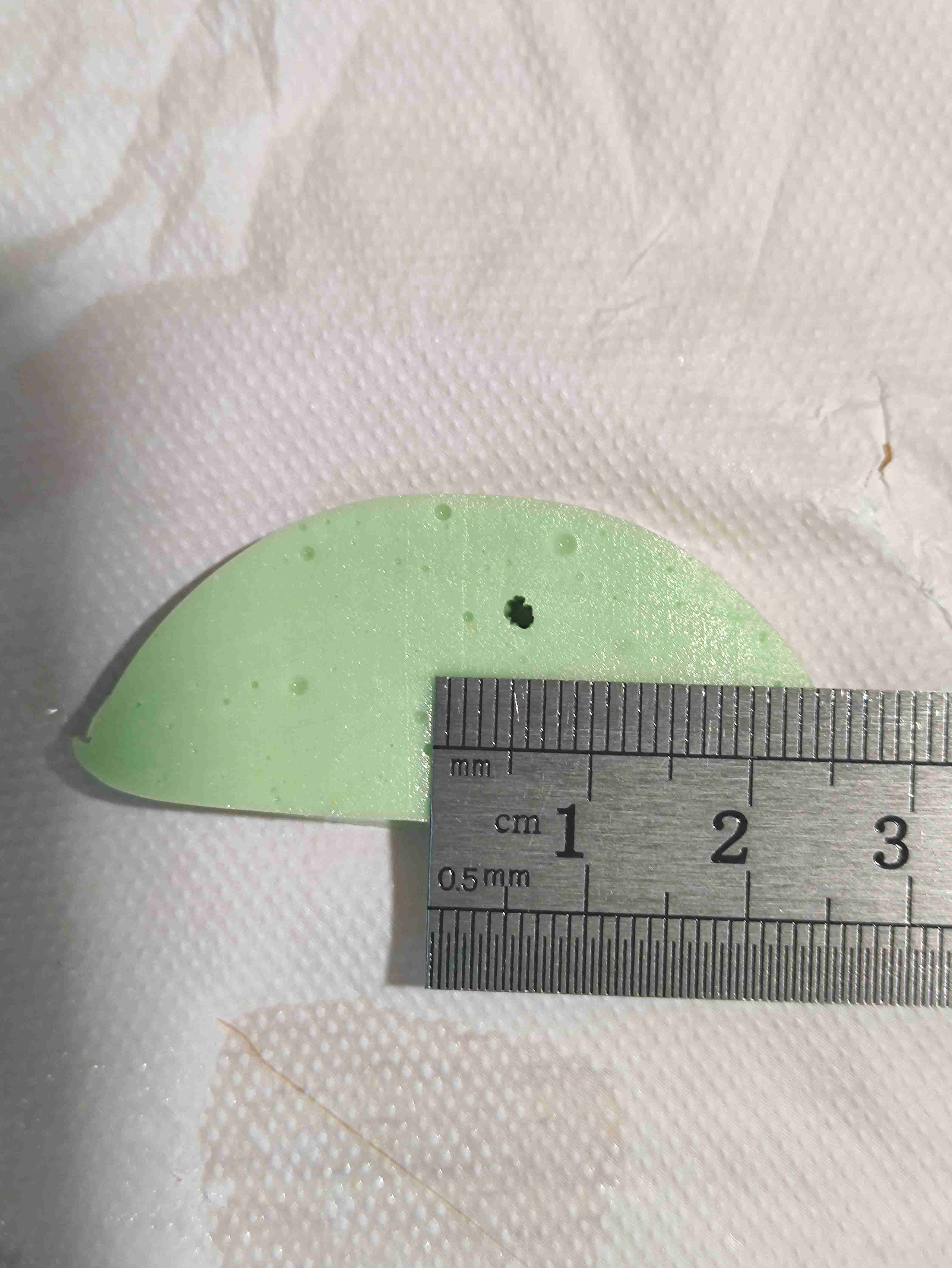

Supplement: Supplementary file 1 [file Data_Sheet_1.zip › Mechanical Characteristics (Table 2)/Cross-sectional Area (Alginate Impression)/C2-1.jpg]

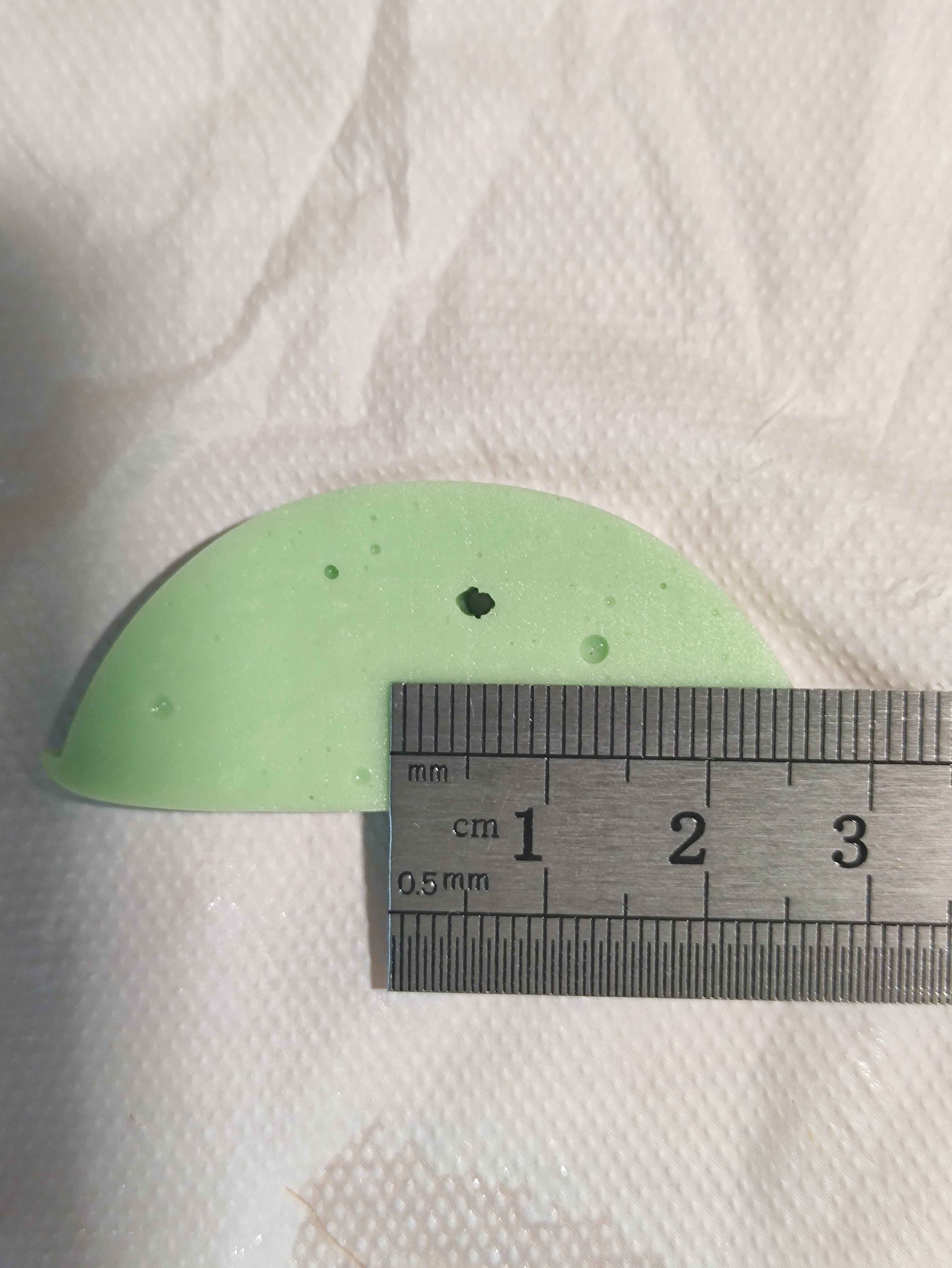

Supplement: Supplementary file 1 [file Data_Sheet_1.zip › Mechanical Characteristics (Table 2)/Cross-sectional Area (Alginate Impression)/C2-2.jpg]

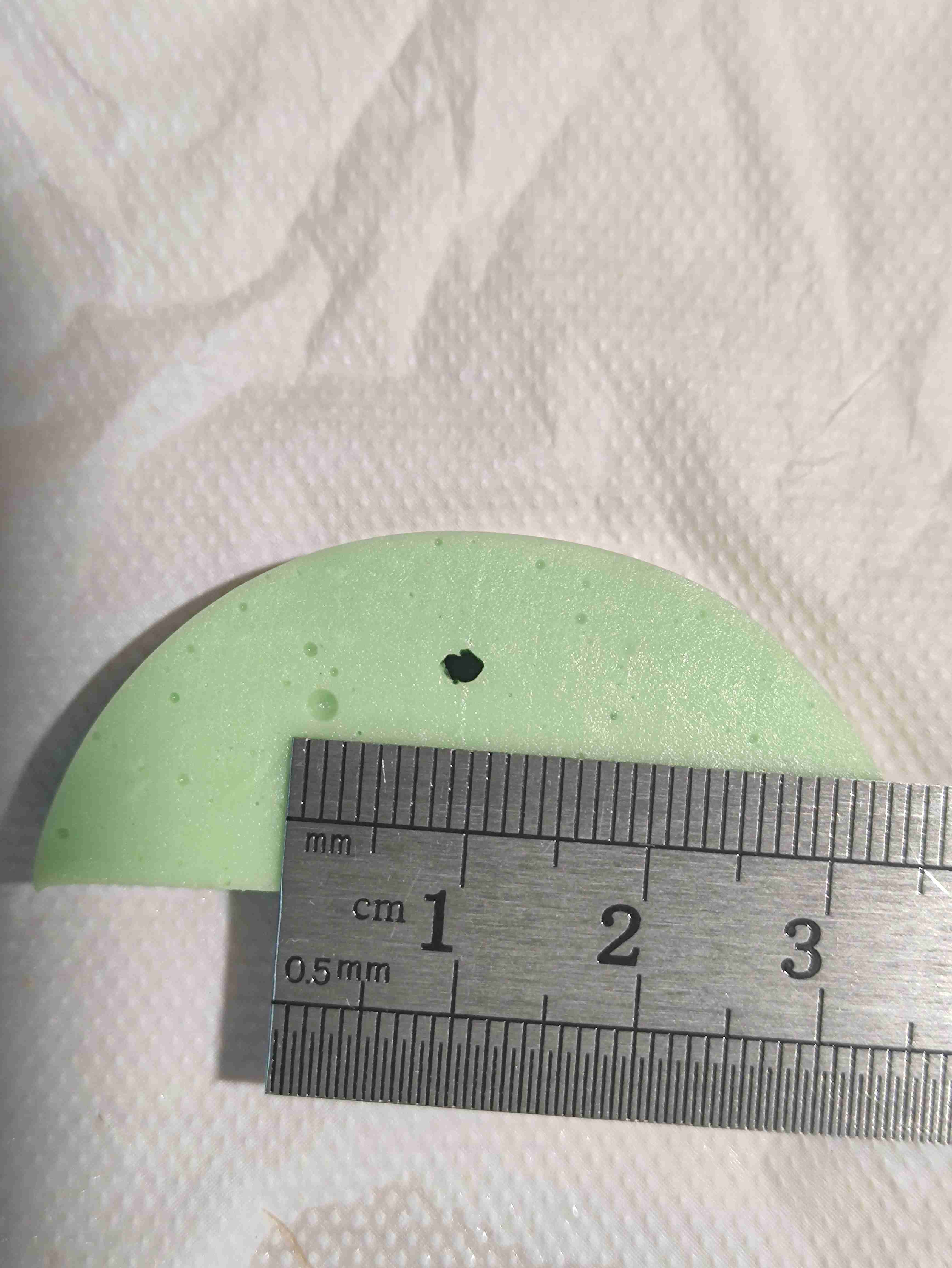

Supplement: Supplementary file 1 [file Data_Sheet_1.zip › Mechanical Characteristics (Table 2)/Cross-sectional Area (Alginate Impression)/C2-3.jpg]

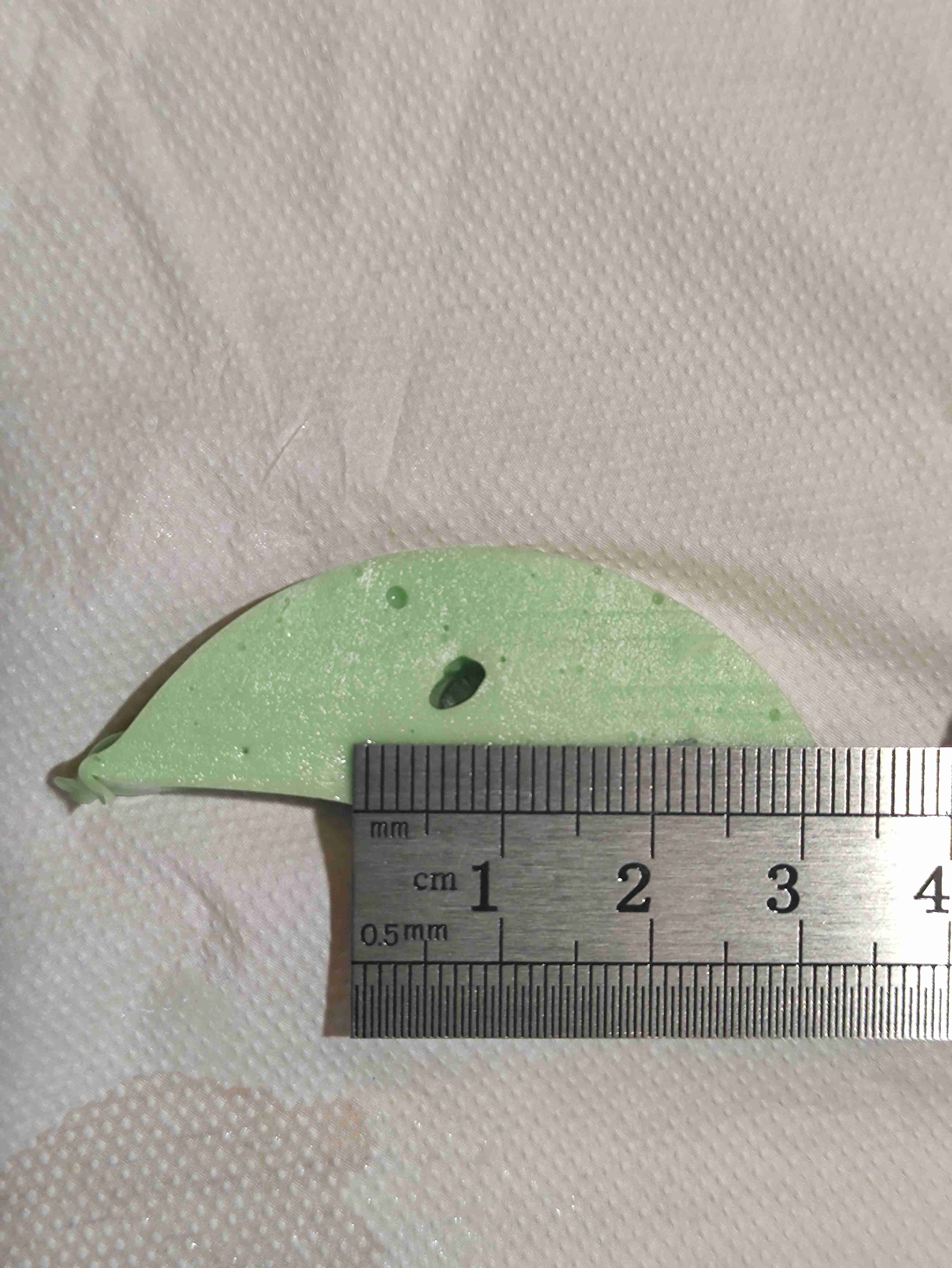

Supplement: Supplementary file 1 [file Data_Sheet_1.zip › Mechanical Characteristics (Table 2)/Cross-sectional Area (Alginate Impression)/D1-1.jpg]

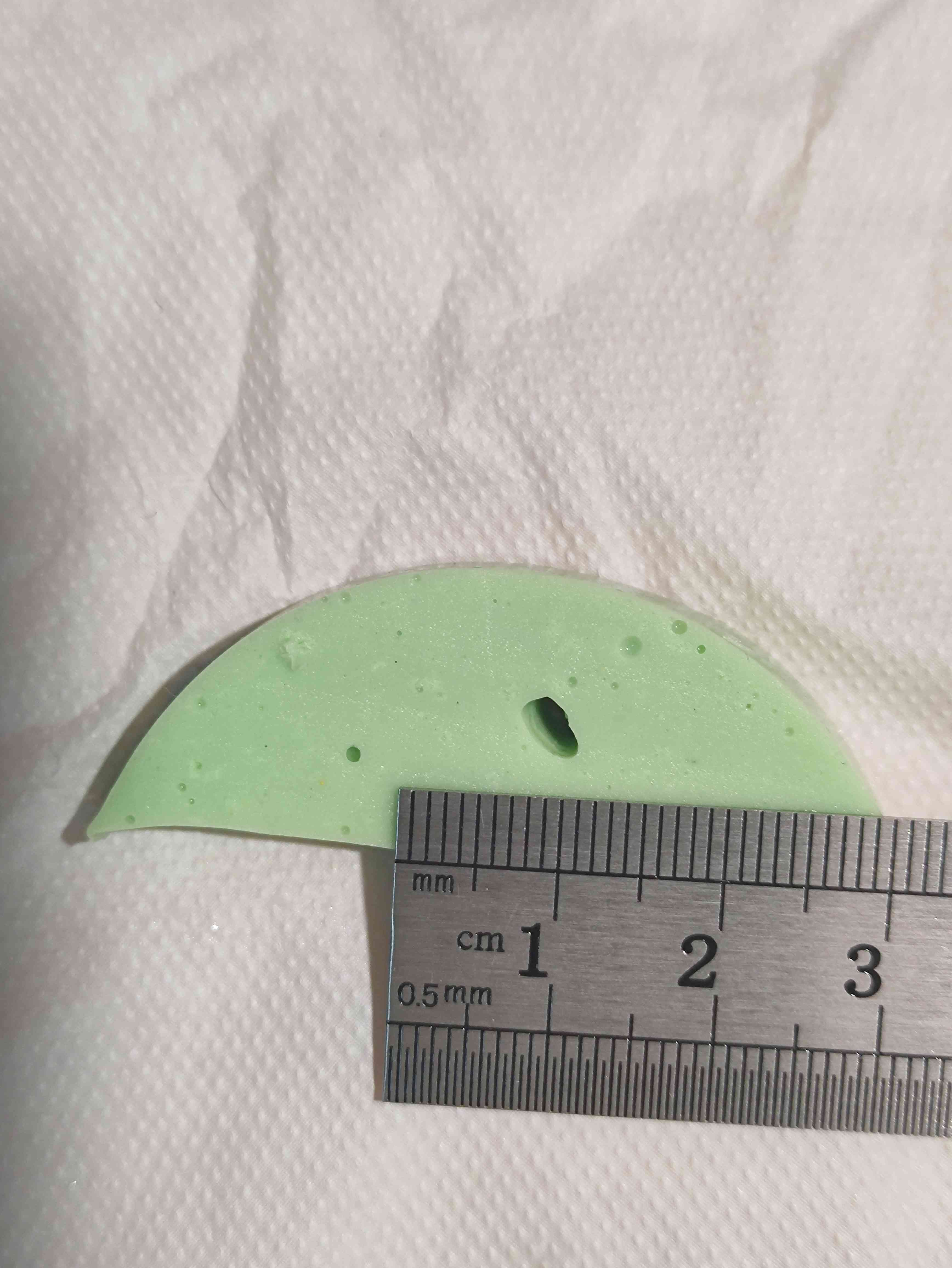

Supplement: Supplementary file 1 [file Data_Sheet_1.zip › Mechanical Characteristics (Table 2)/Cross-sectional Area (Alginate Impression)/D1-2.jpg]

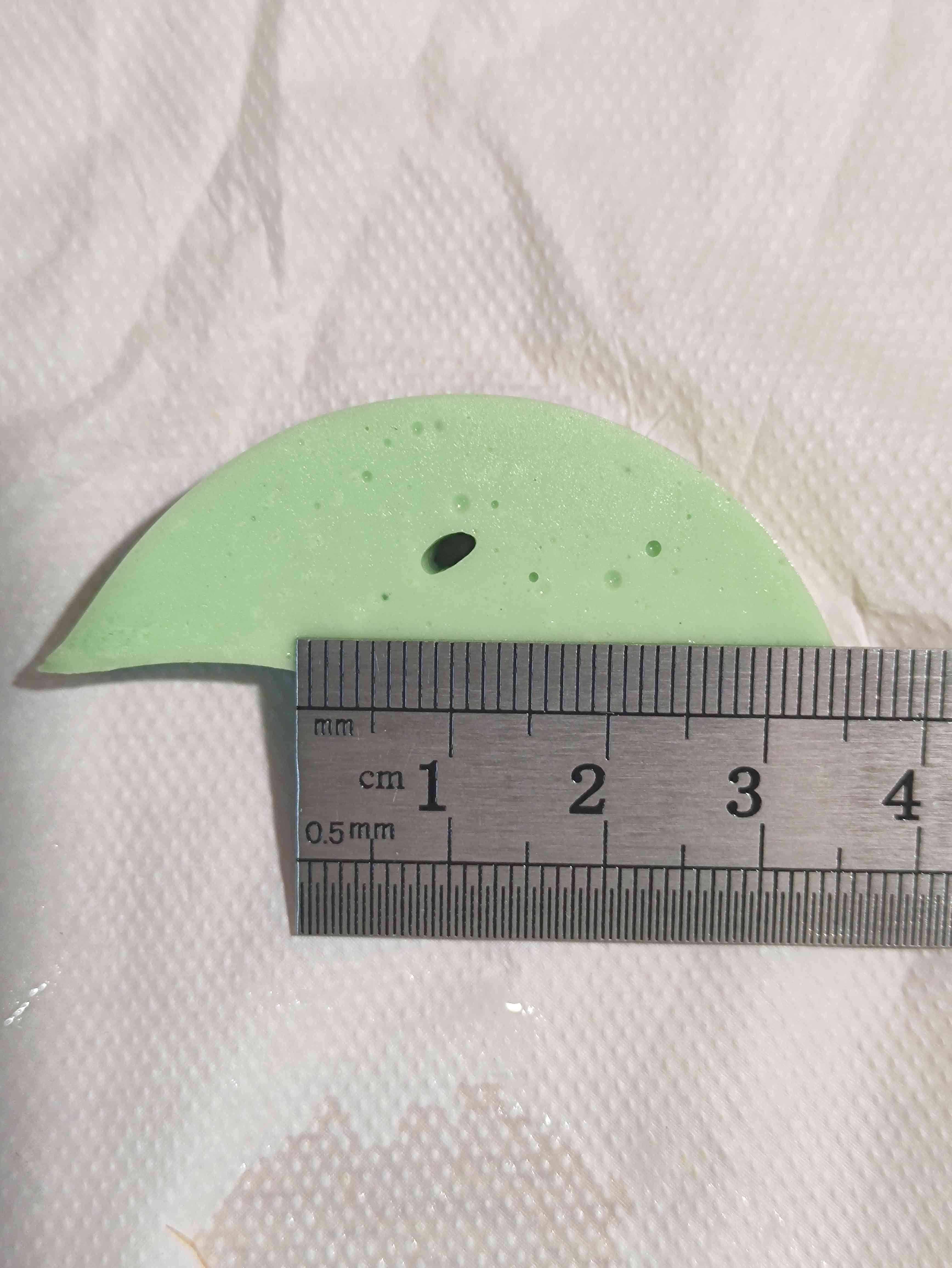

Supplement: Supplementary file 1 [file Data_Sheet_1.zip › Mechanical Characteristics (Table 2)/Cross-sectional Area (Alginate Impression)/D1-3.jpg]

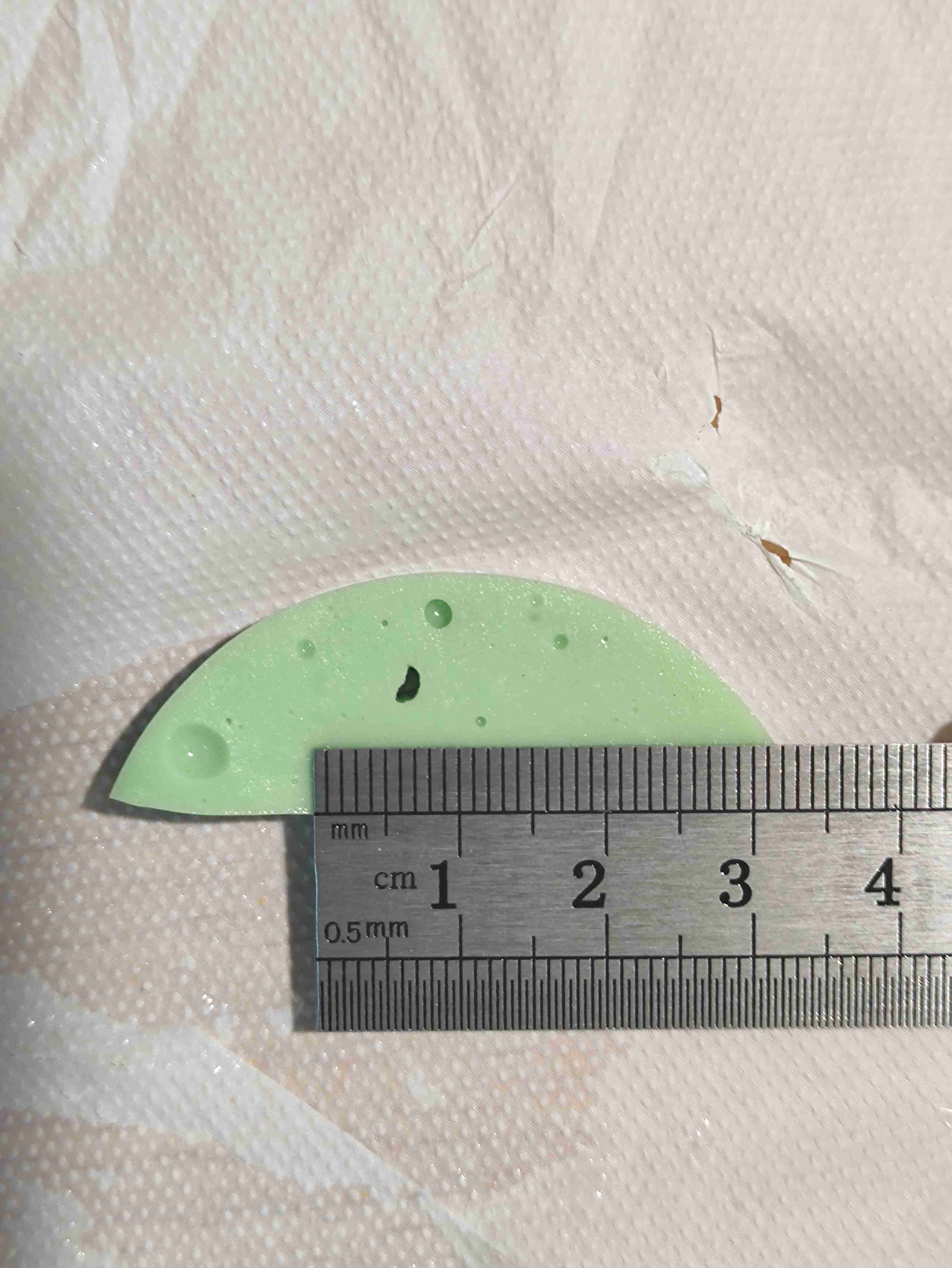

Supplement: Supplementary file 1 [file Data_Sheet_1.zip › Mechanical Characteristics (Table 2)/Cross-sectional Area (Alginate Impression)/D2-1.jpg]

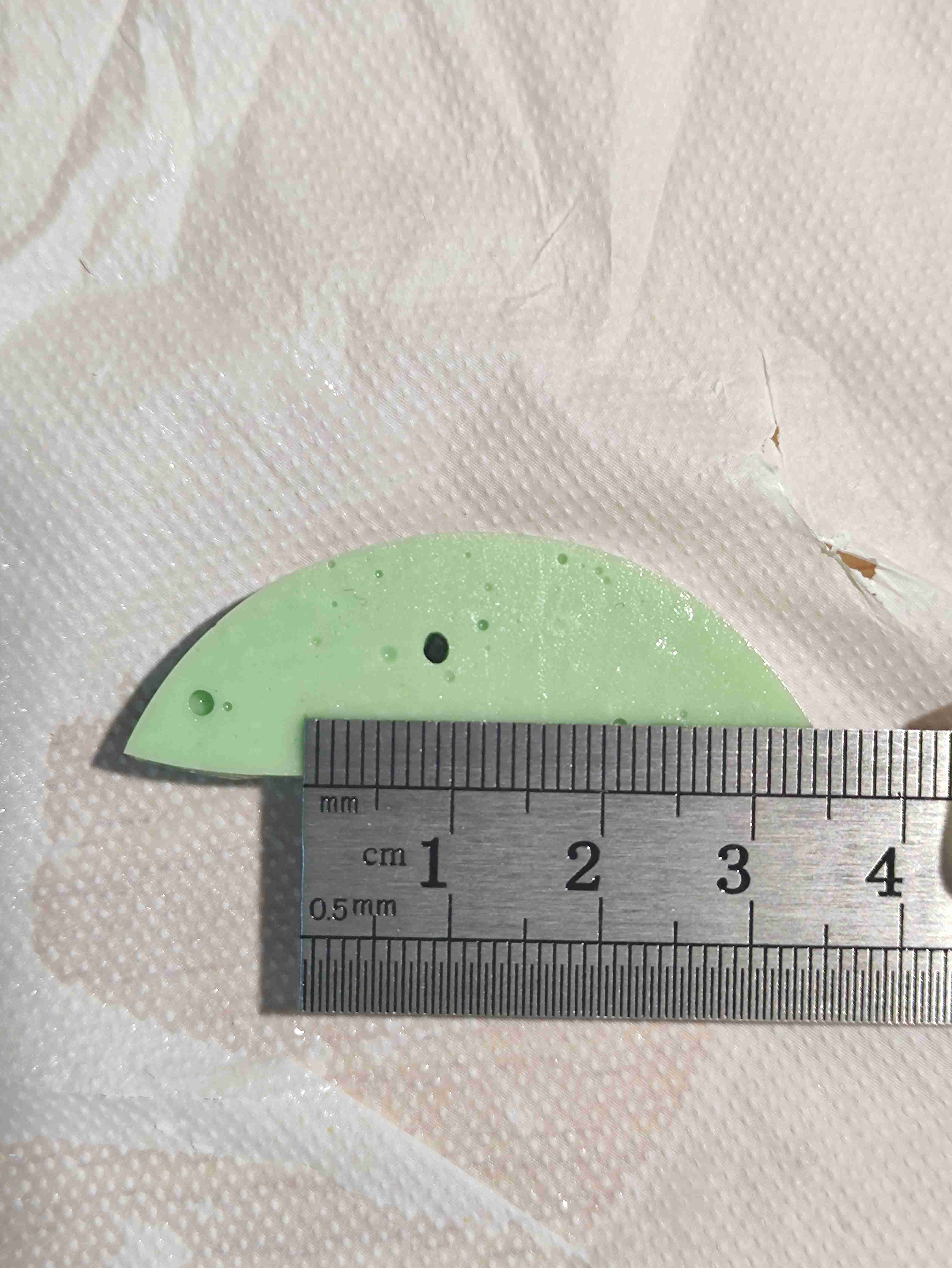

Supplement: Supplementary file 1 [file Data_Sheet_1.zip › Mechanical Characteristics (Table 2)/Cross-sectional Area (Alginate Impression)/D2-2.jpg]

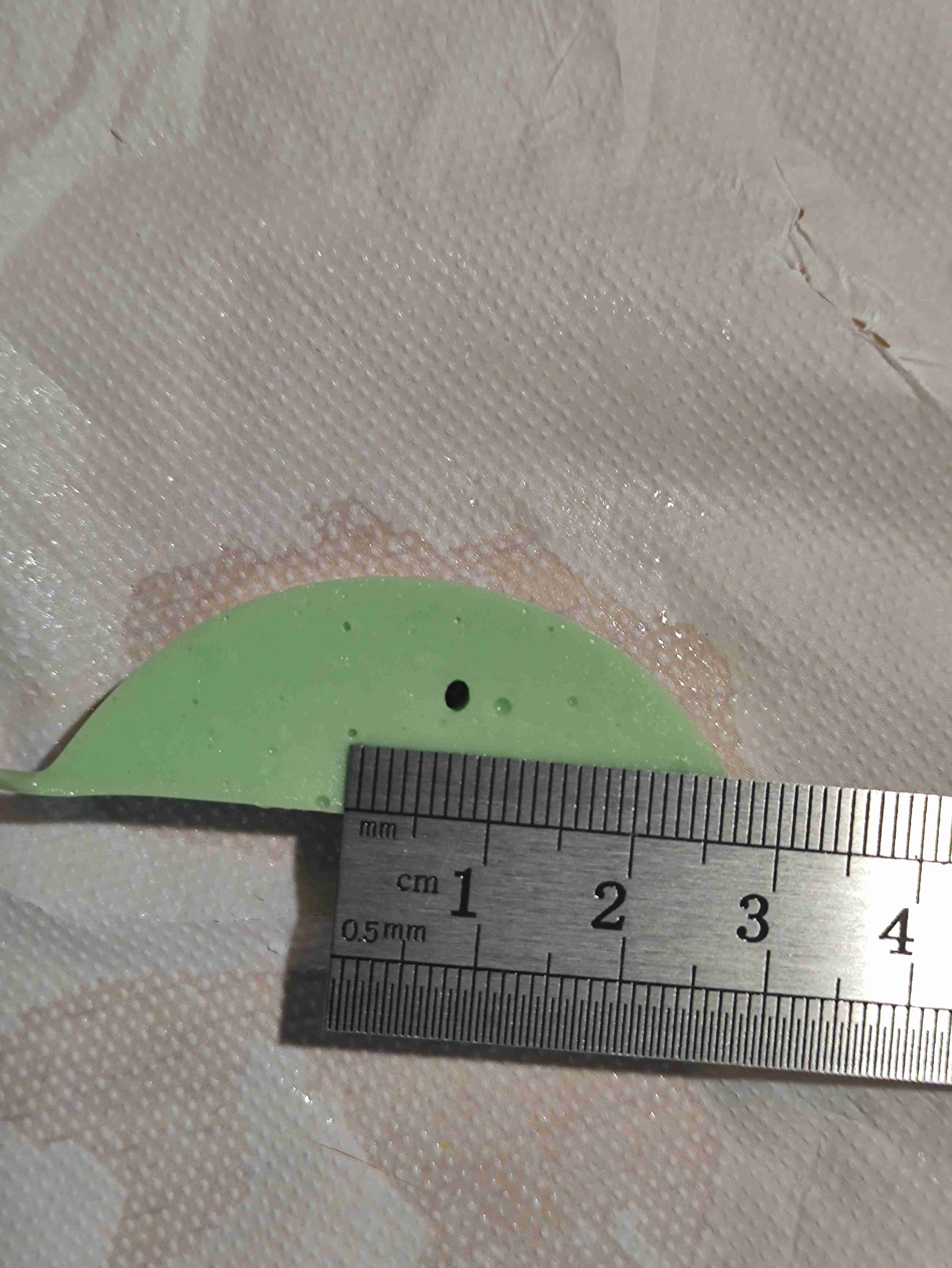

Supplement: Supplementary file 1 [file Data_Sheet_1.zip › Mechanical Characteristics (Table 2)/Cross-sectional Area (Alginate Impression)/D2-3.jpg]

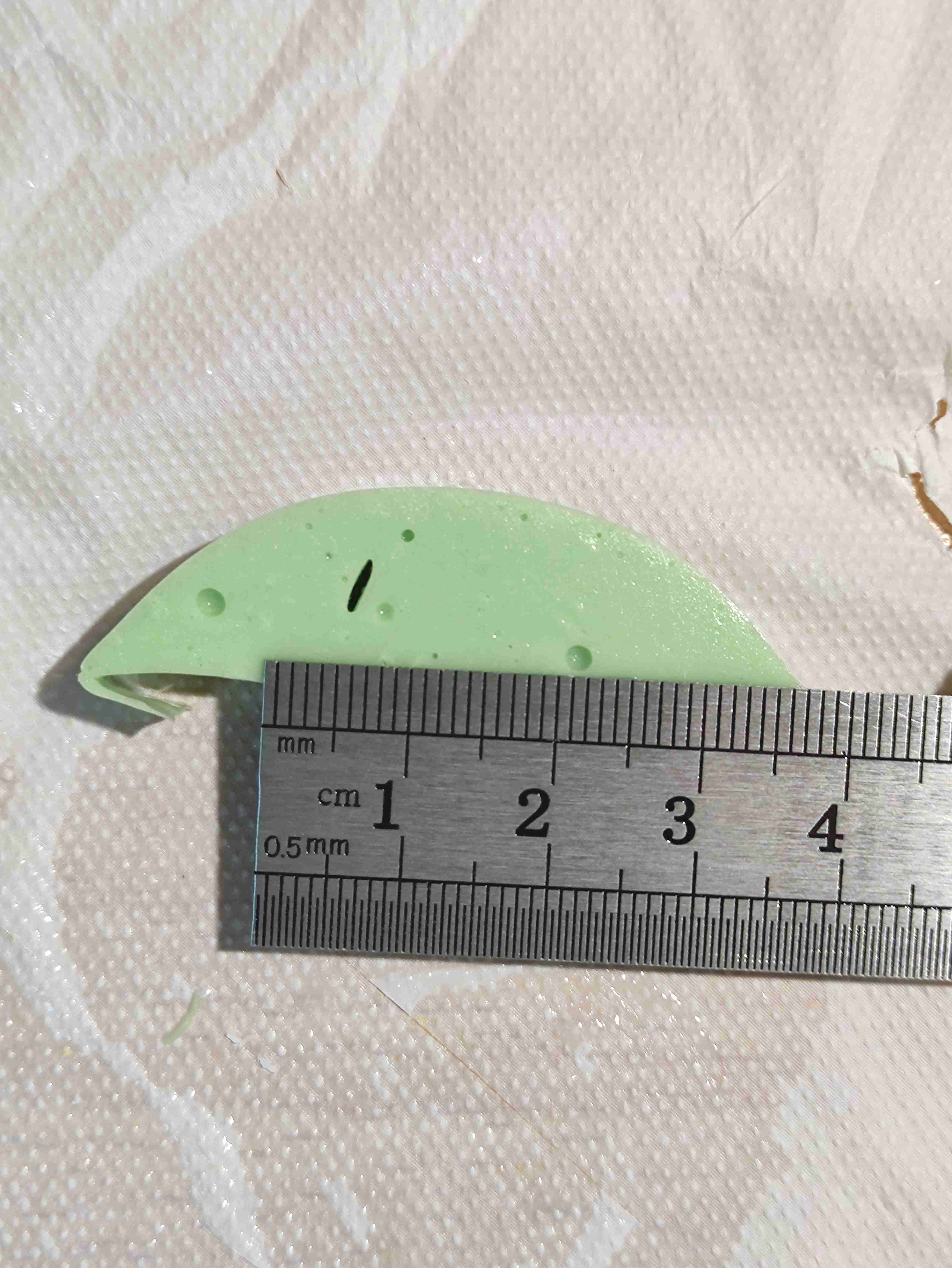

Supplement: Supplementary file 1 [file Data_Sheet_1.zip › Mechanical Characteristics (Table 2)/Cross-sectional Area (Alginate Impression)/E1-1.jpg]

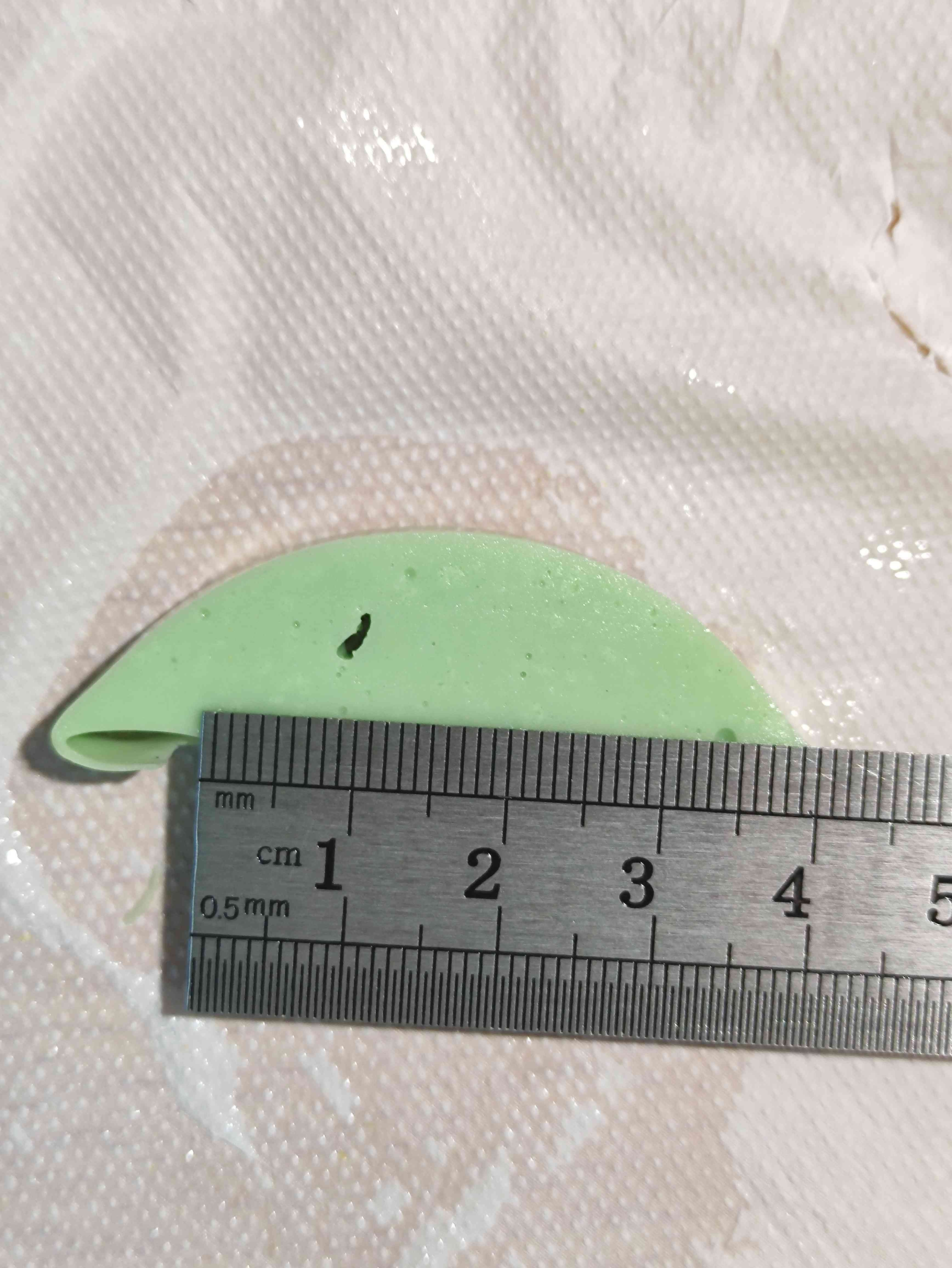

Supplement: Supplementary file 1 [file Data_Sheet_1.zip › Mechanical Characteristics (Table 2)/Cross-sectional Area (Alginate Impression)/E1-2.jpg]

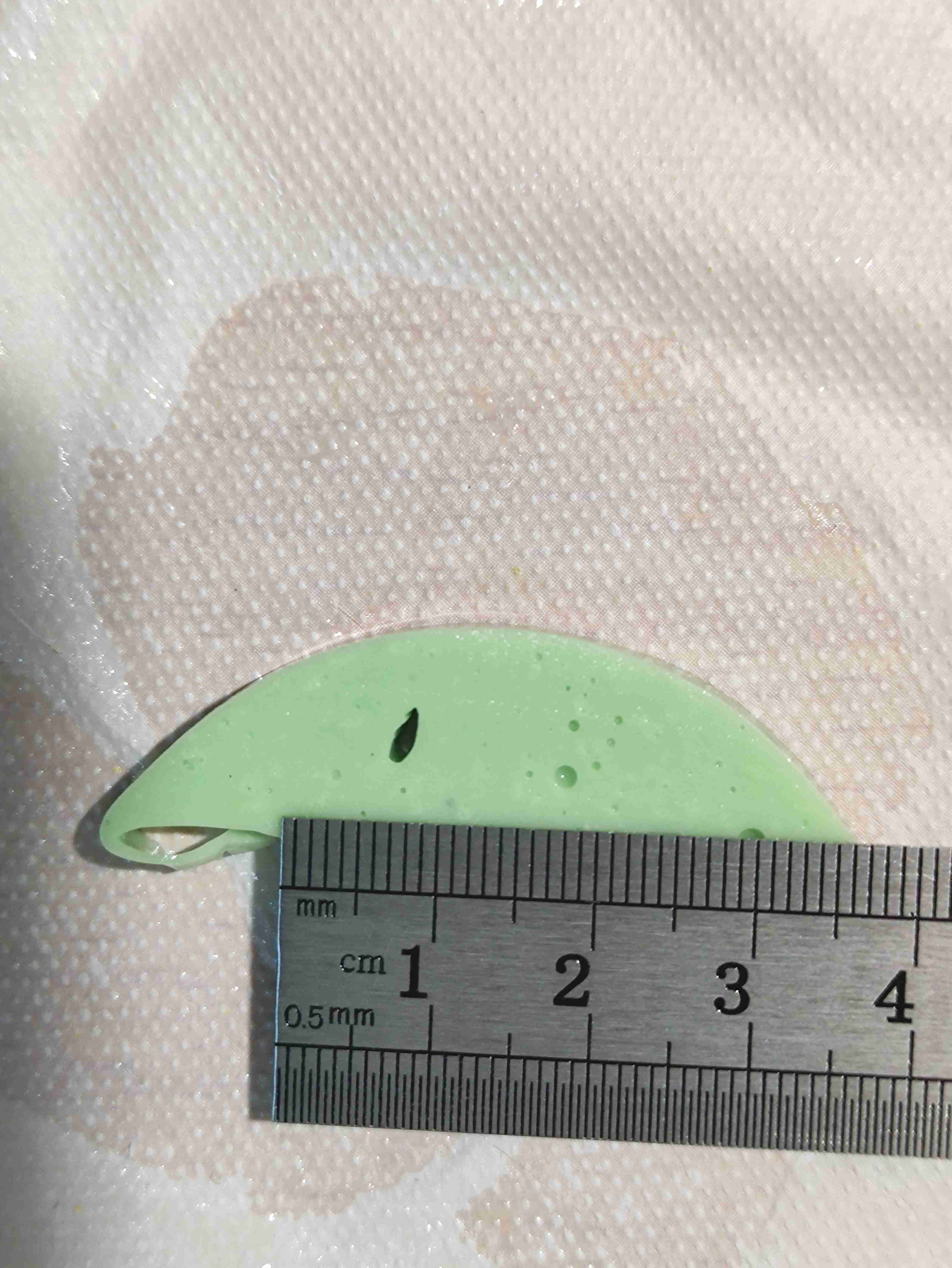

Supplement: Supplementary file 1 [file Data_Sheet_1.zip › Mechanical Characteristics (Table 2)/Cross-sectional Area (Alginate Impression)/E1-3.jpg]

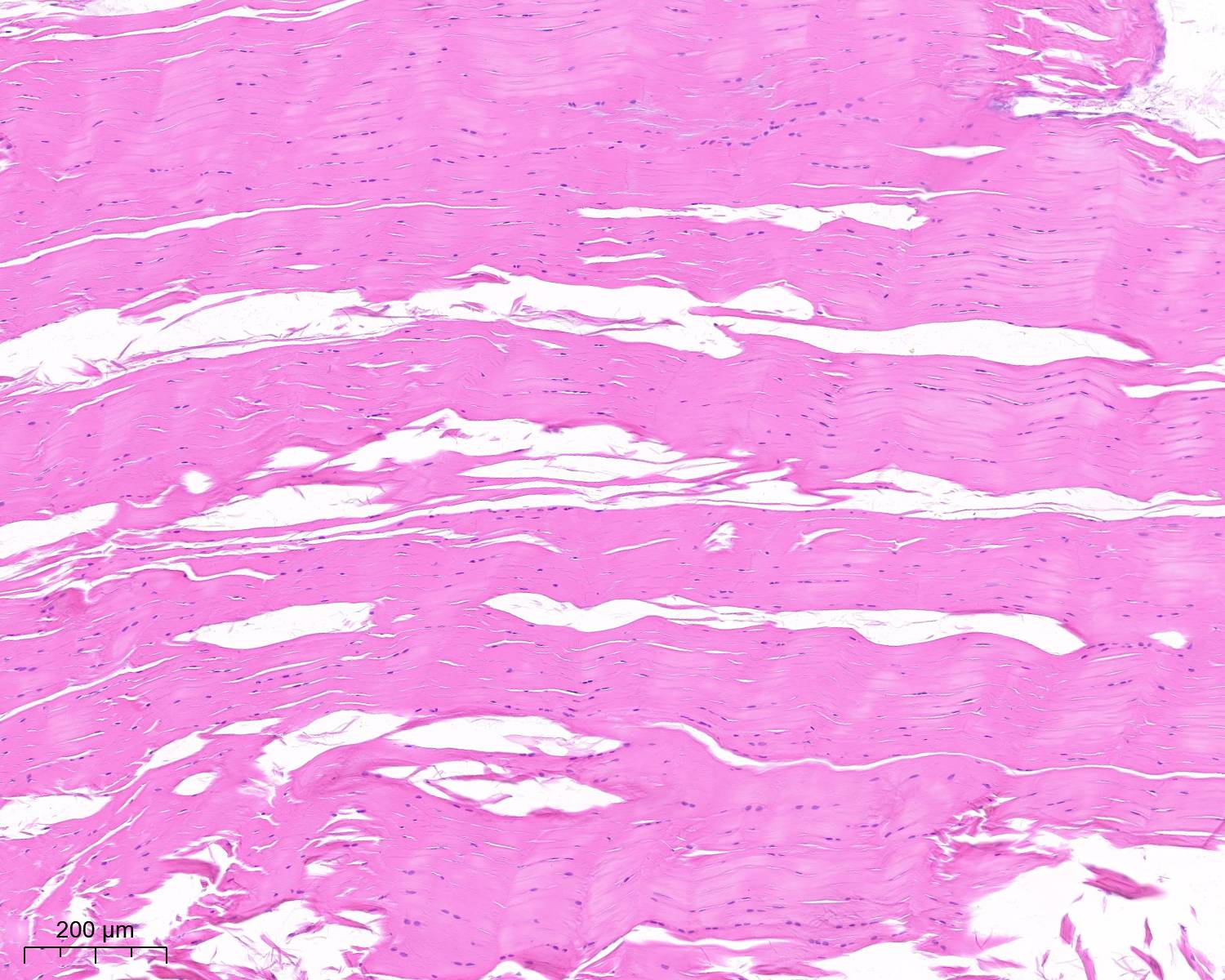

Supplement: Supplementary file 1 [file Data_Sheet_1.zip › Morphological figure/0min 01(1).jpg]

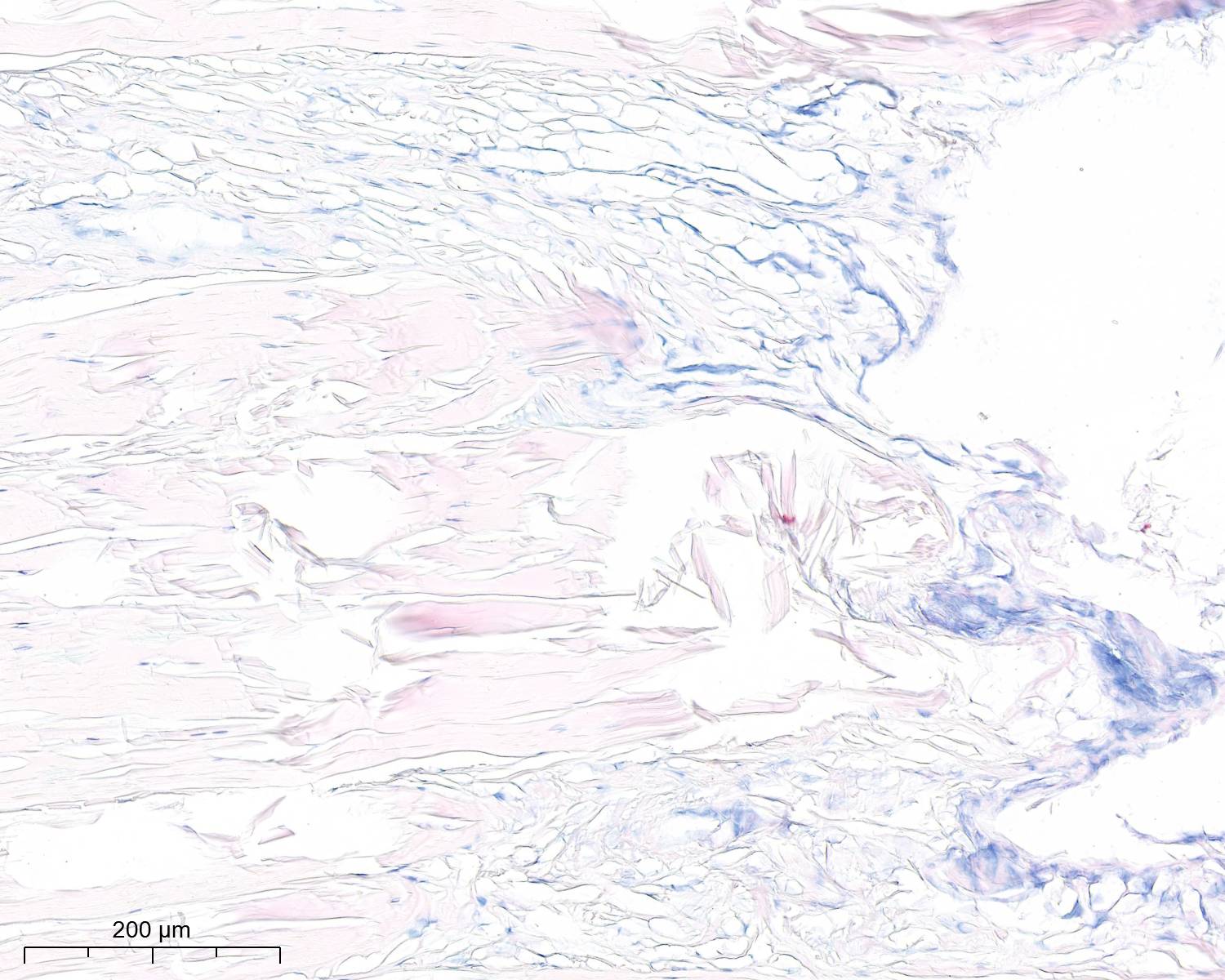

Supplement: Supplementary file 1 [file Data_Sheet_1.zip › Morphological figure/0min 01(1)2.jpg]

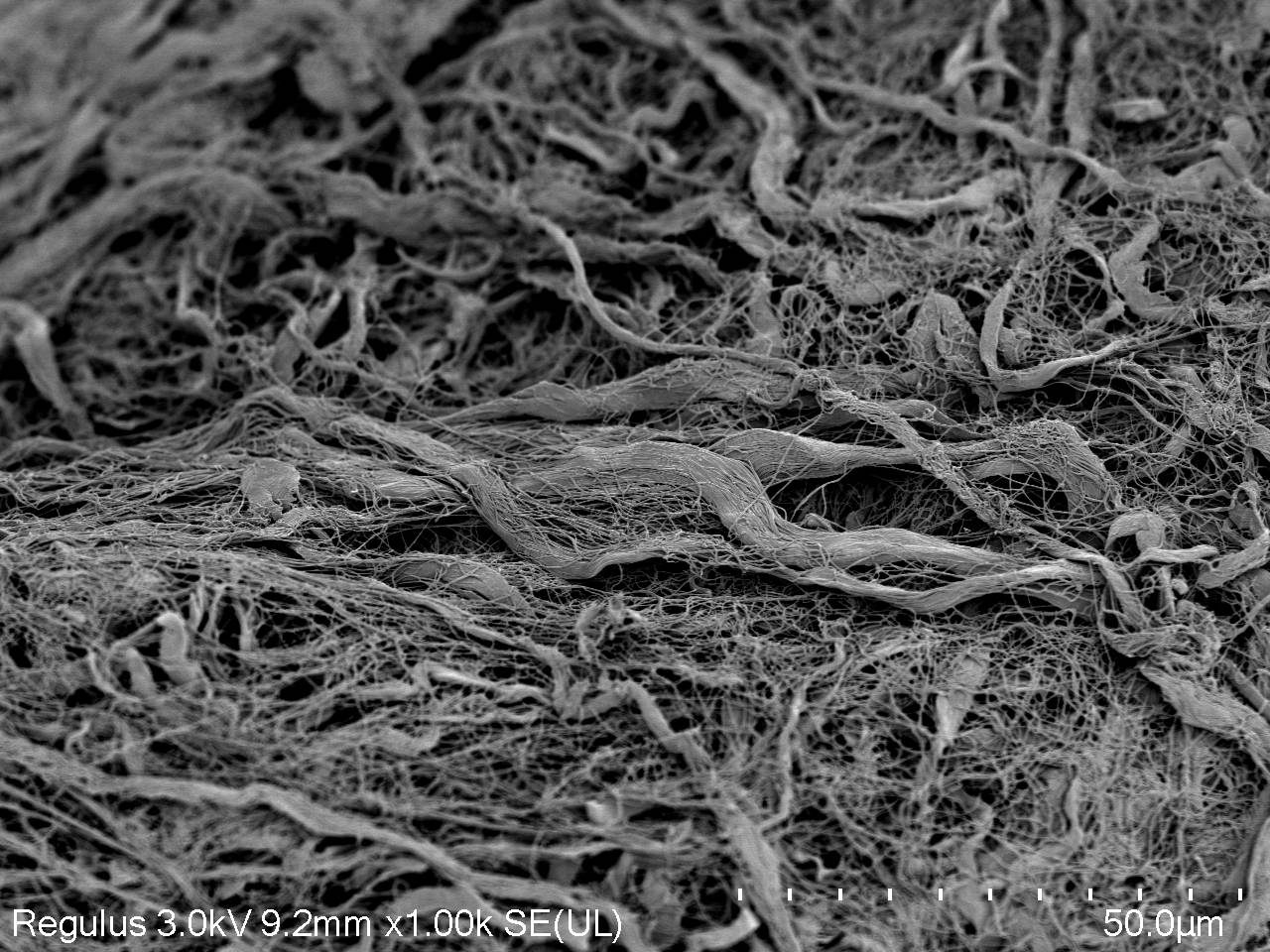

Supplement: Supplementary file 1 [file Data_Sheet_1.zip › Morphological figure/0min 01.1(1).jpg]

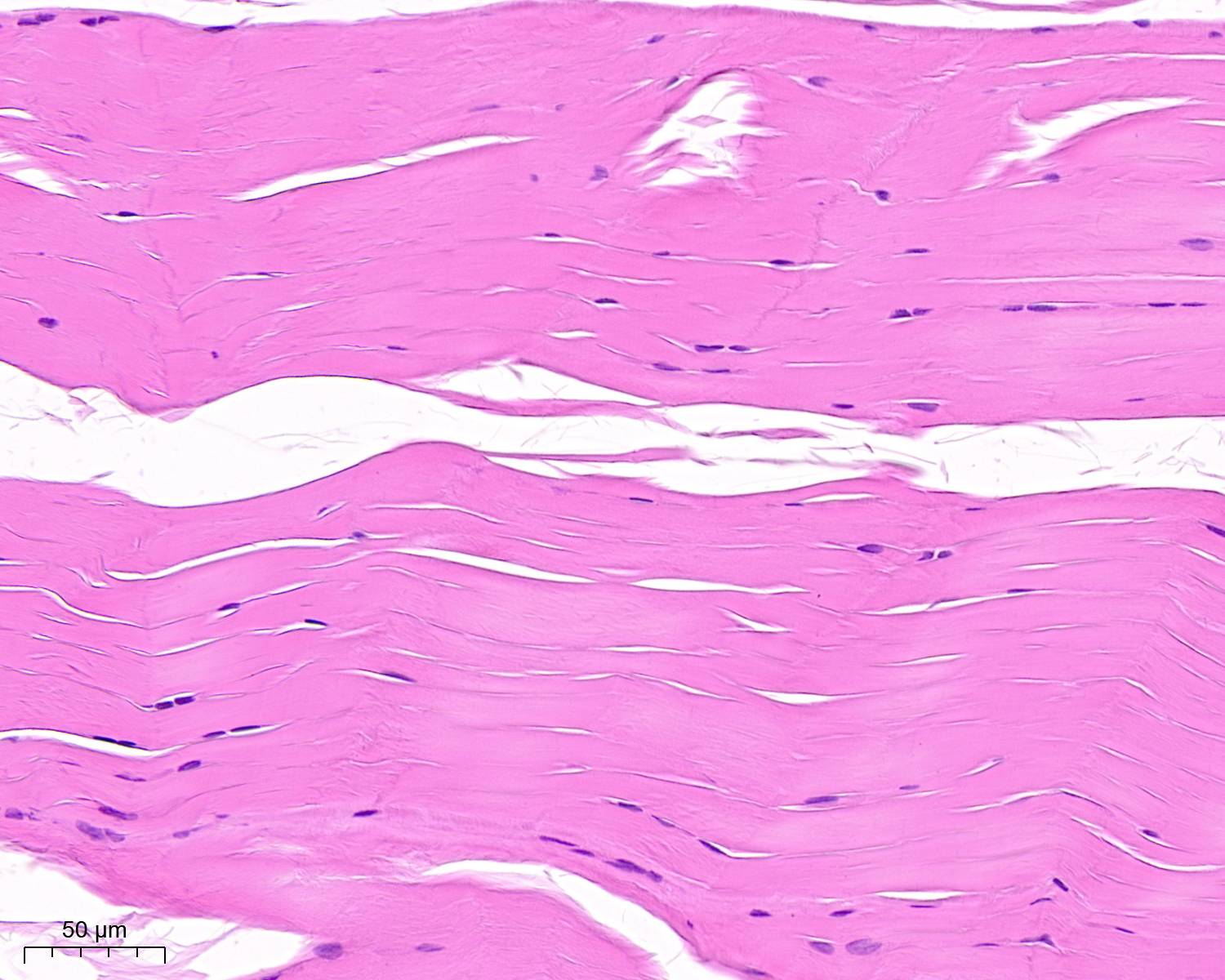

Supplement: Supplementary file 1 [file Data_Sheet_1.zip › Morphological figure/0min 02(1).jpg]

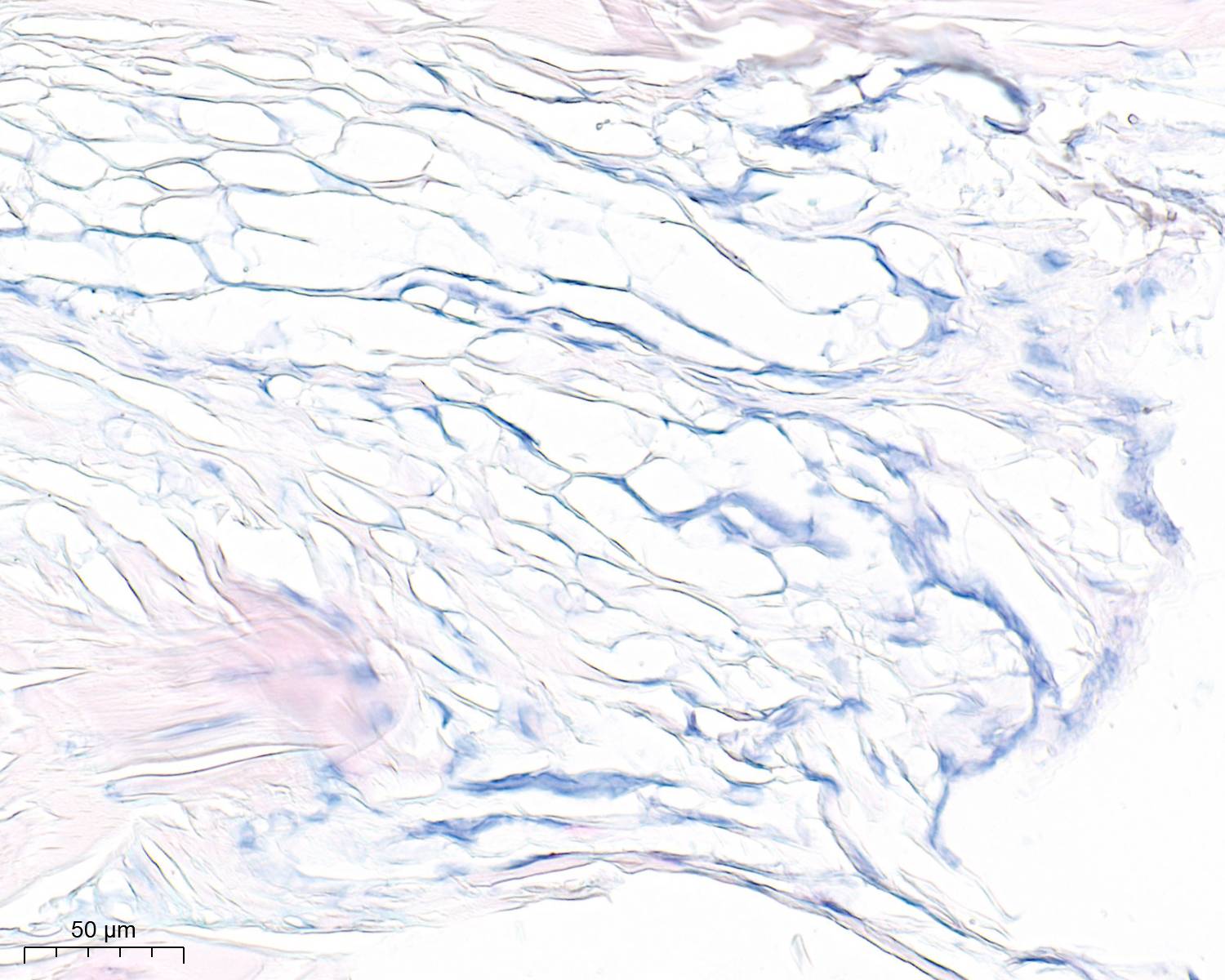

Supplement: Supplementary file 1 [file Data_Sheet_1.zip › Morphological figure/0min 02(1)2.jpg]

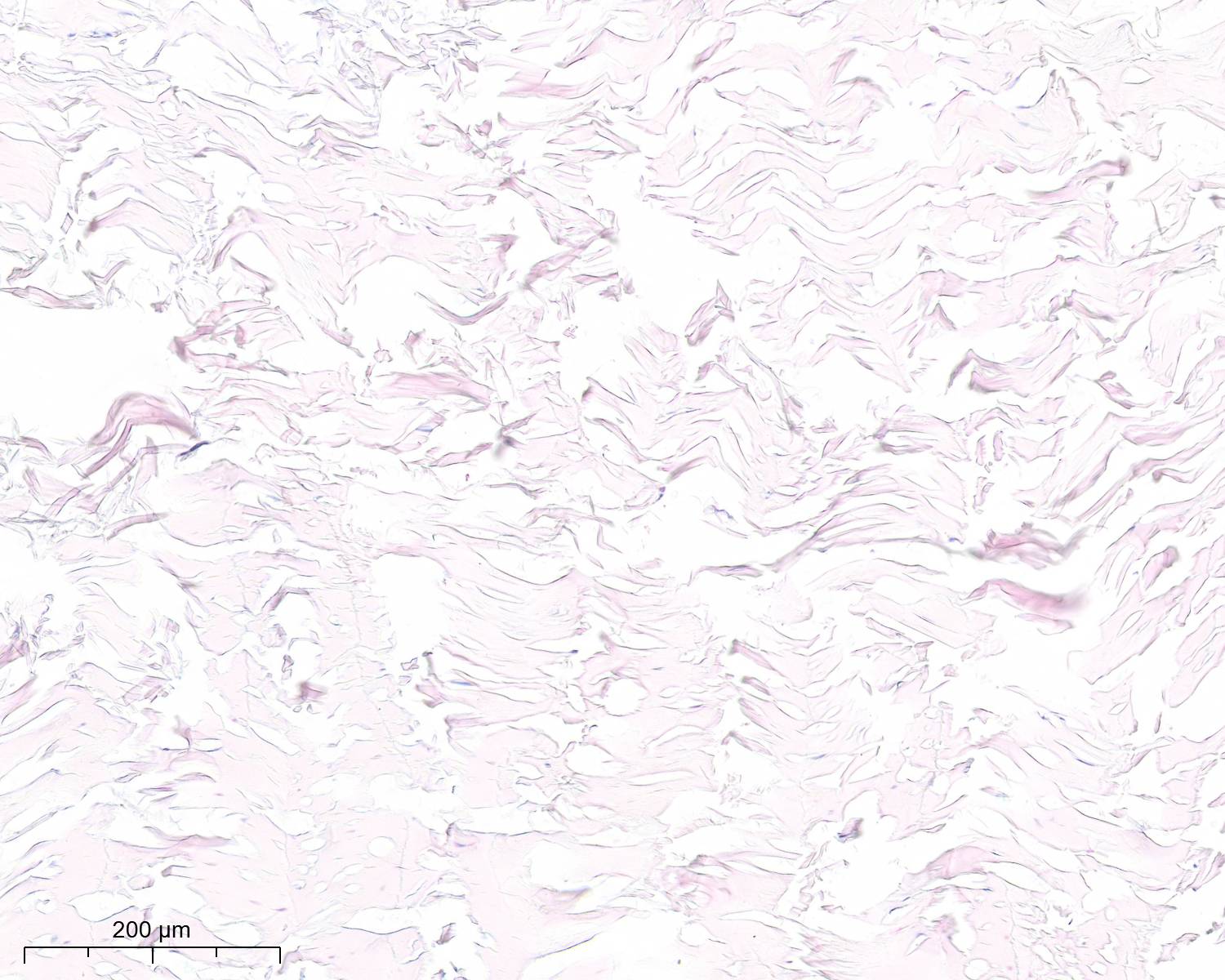

Supplement: Supplementary file 1 [file Data_Sheet_1.zip › Morphological figure/11min 01(1)2.jpg]

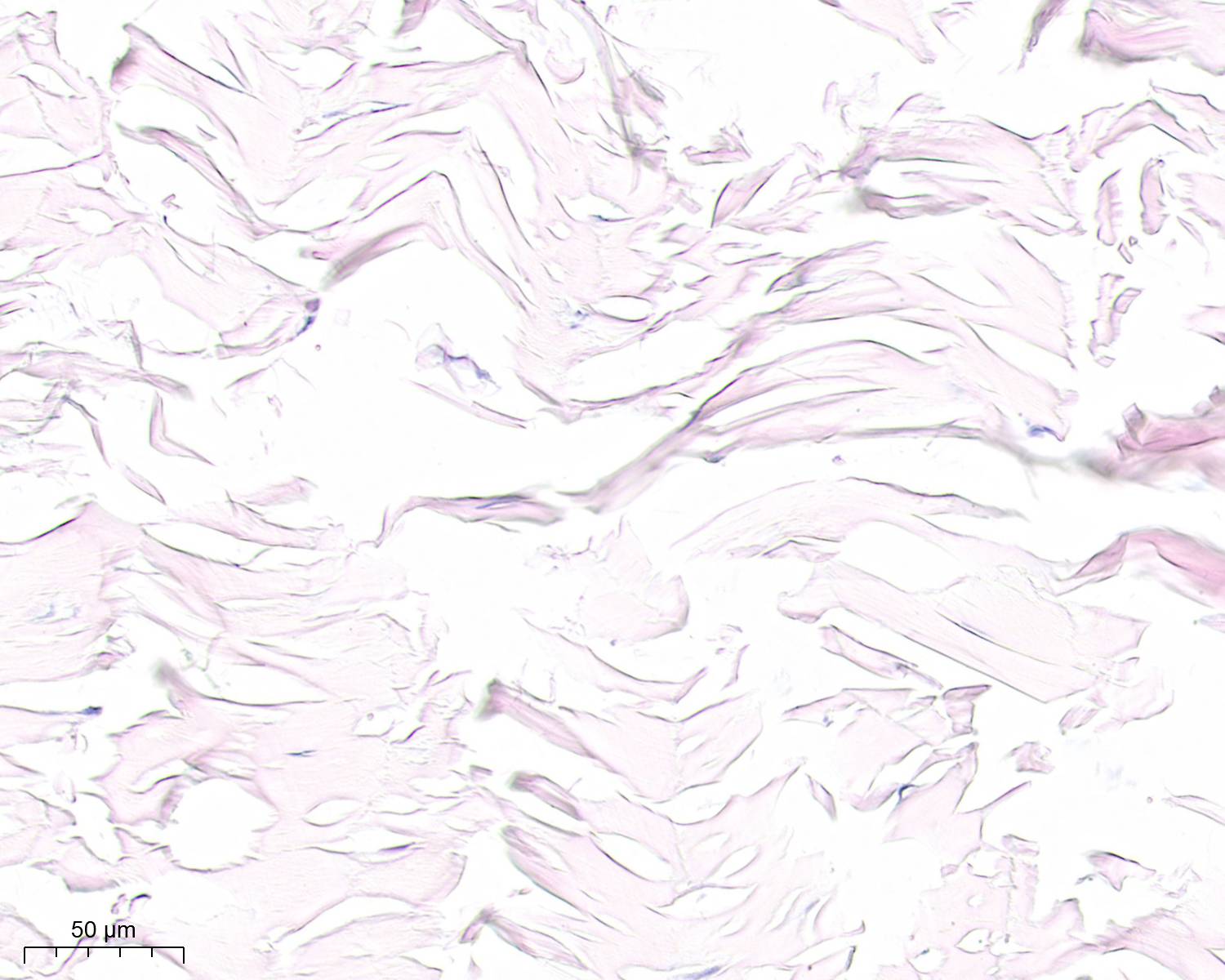

Supplement: Supplementary file 1 [file Data_Sheet_1.zip › Morphological figure/11min 02(1).jpg]

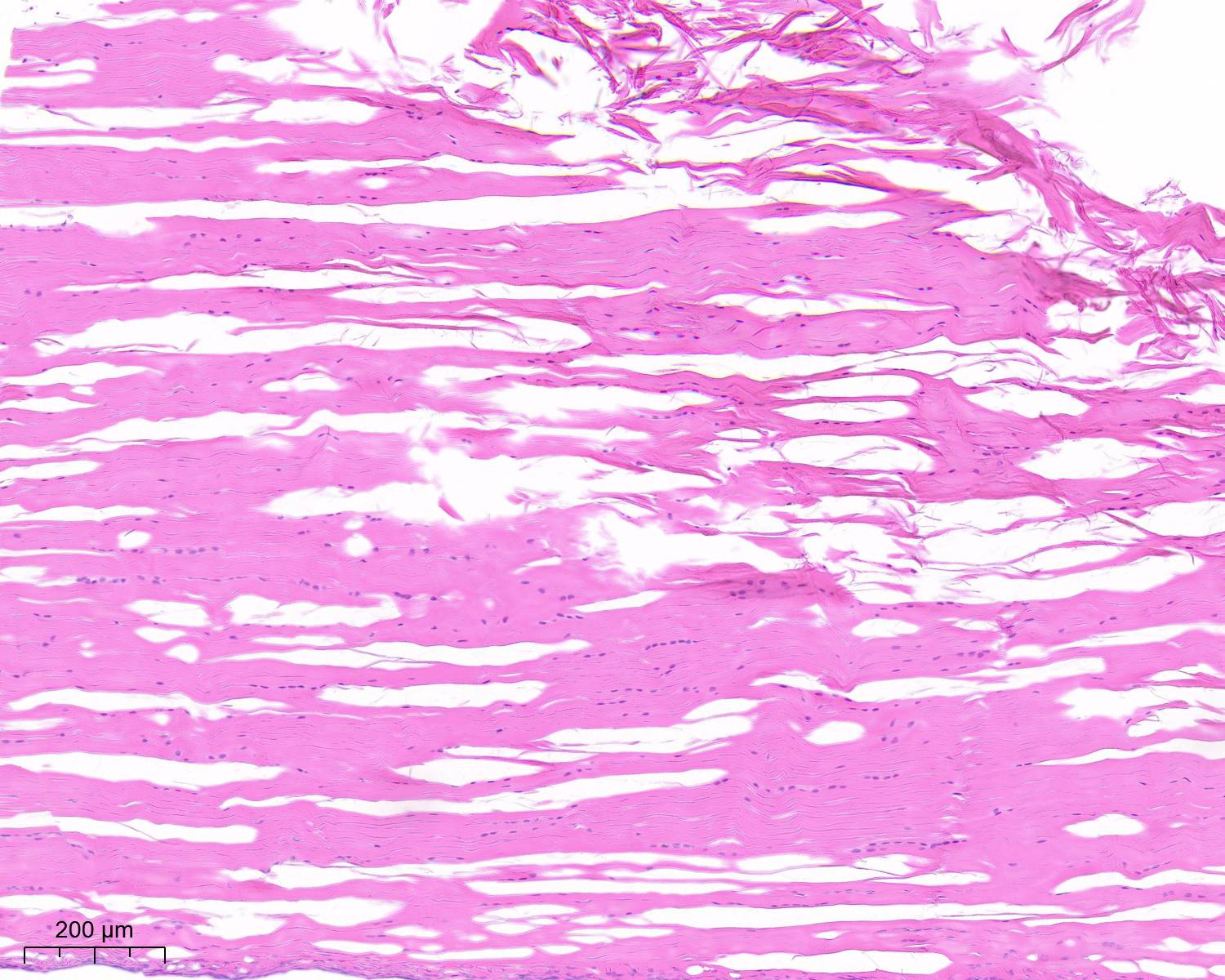

Supplement: Supplementary file 1 [file Data_Sheet_1.zip › Morphological figure/18 min(1).jpg]

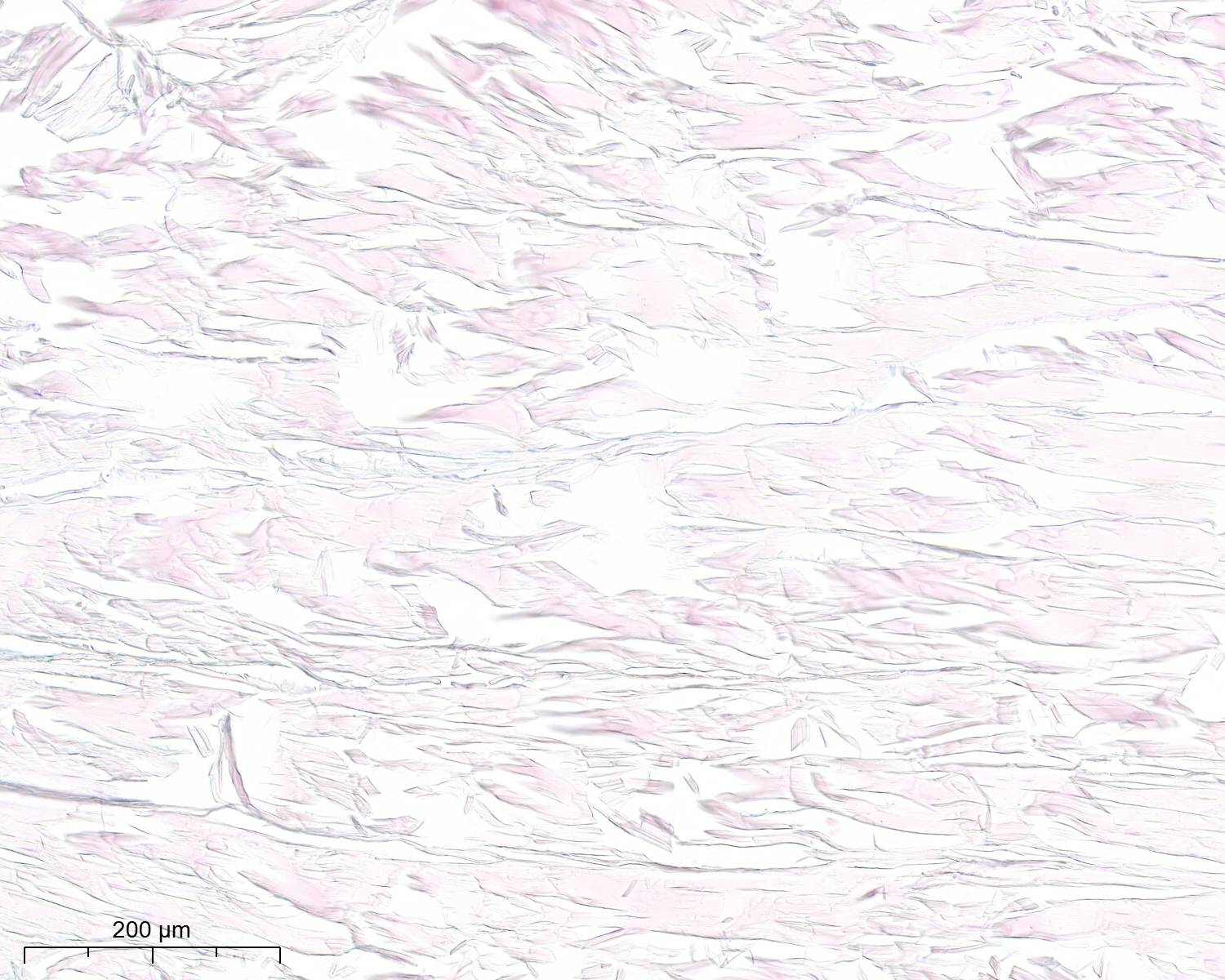

Supplement: Supplementary file 1 [file Data_Sheet_1.zip › Morphological figure/18min 01(1)2.jpg]

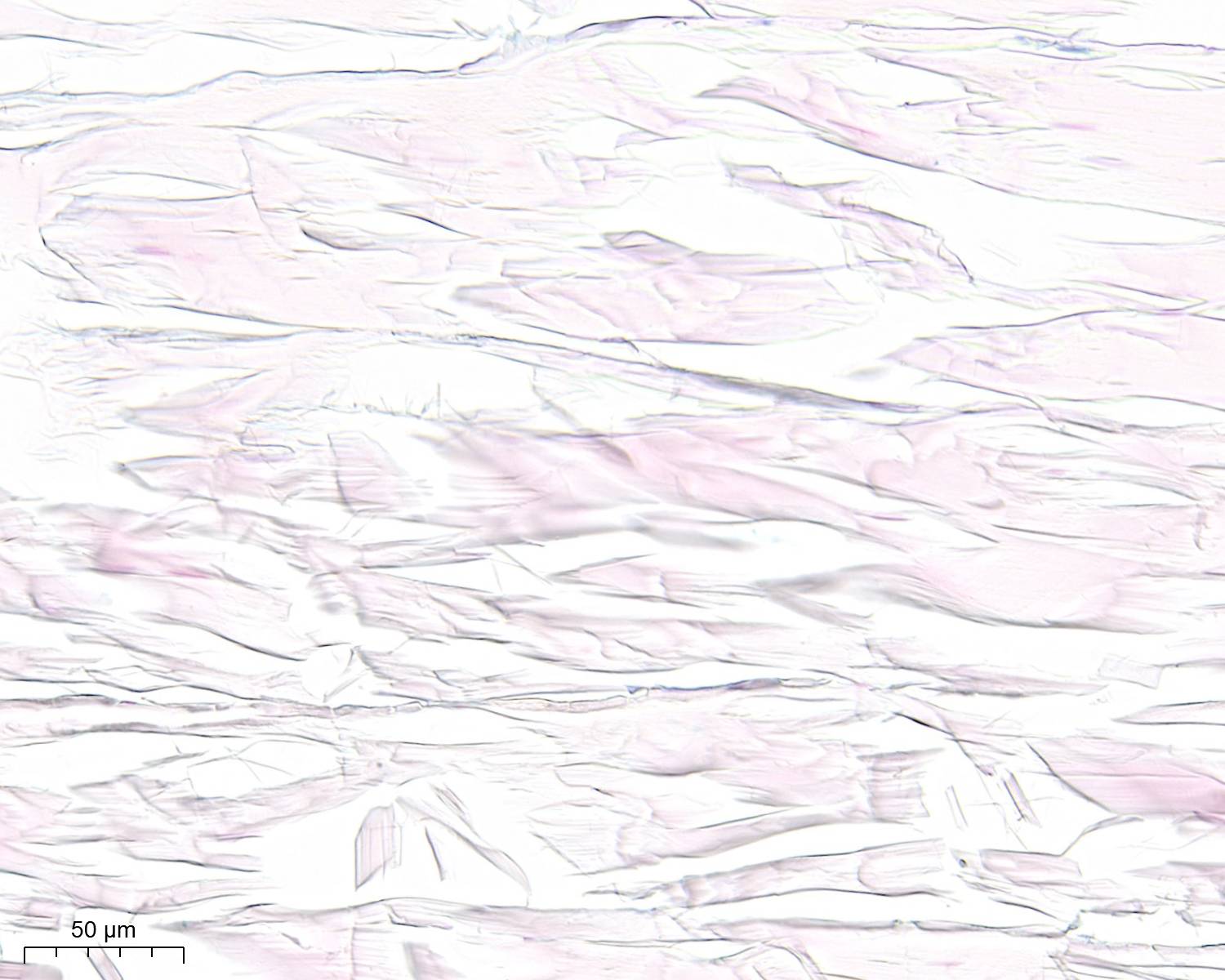

Supplement: Supplementary file 1 [file Data_Sheet_1.zip › Morphological figure/18min 02(1).jpg]

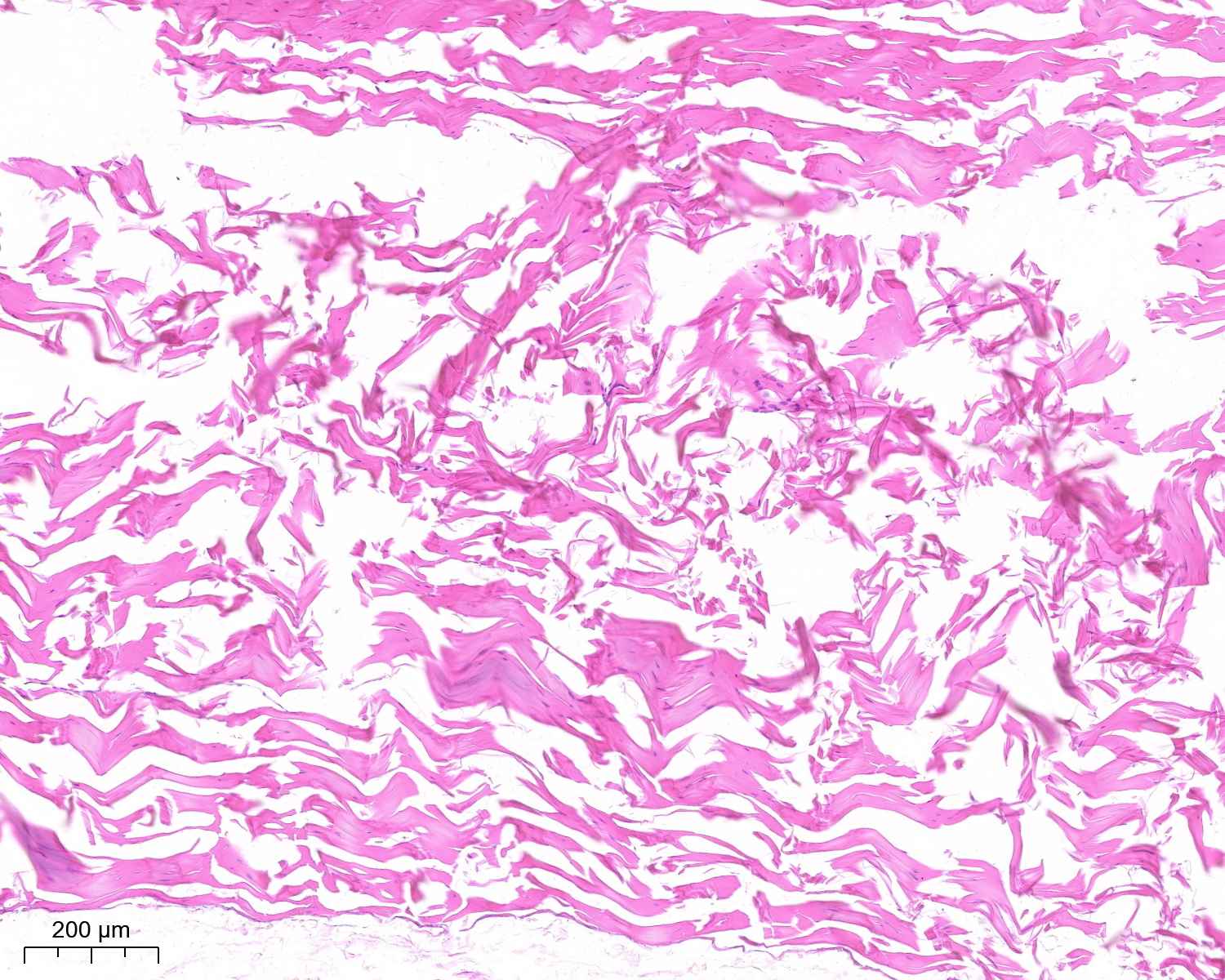

Supplement: Supplementary file 1 [file Data_Sheet_1.zip › Morphological figure/30min 01(1).jpg]

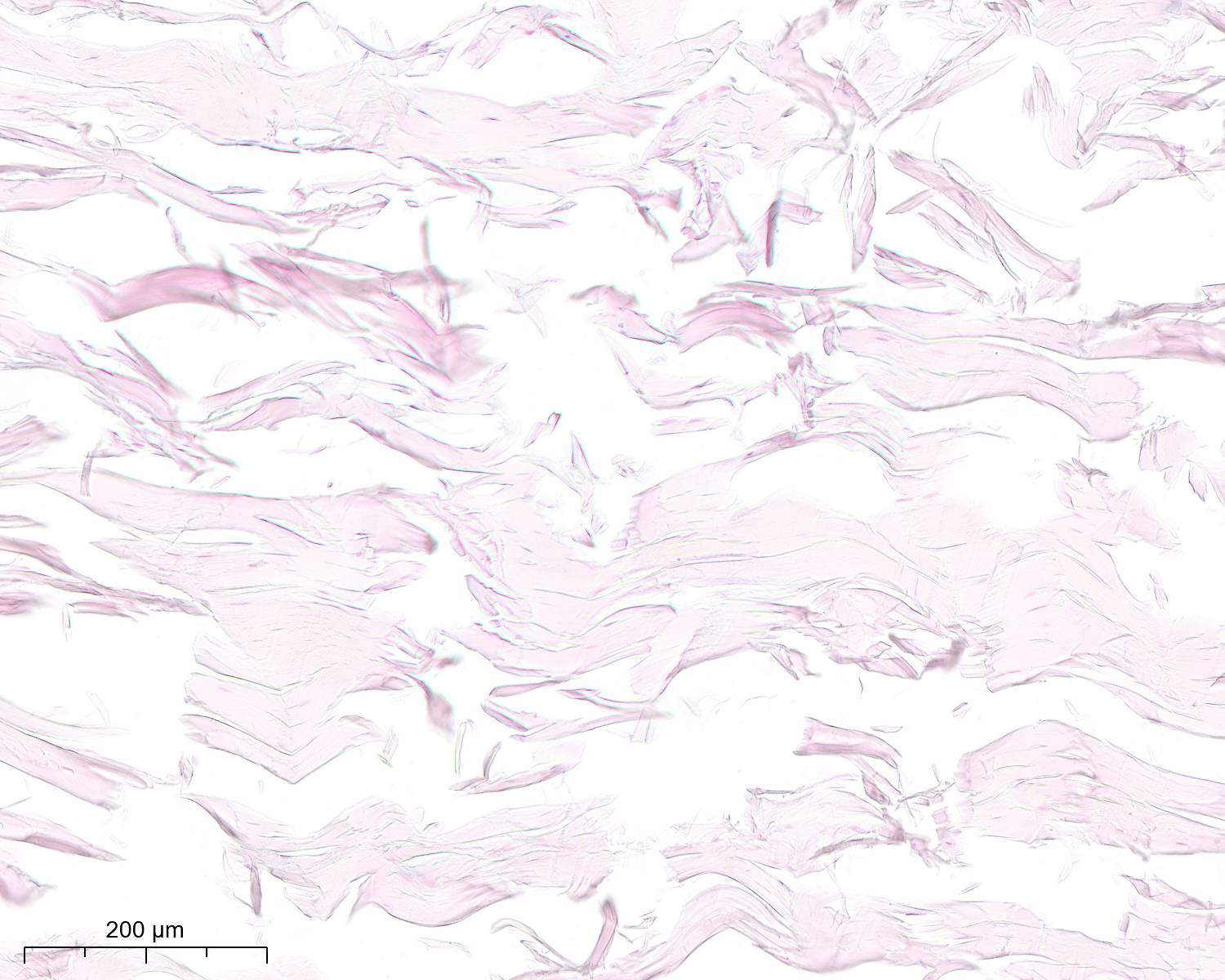

Supplement: Supplementary file 1 [file Data_Sheet_1.zip › Morphological figure/30min 01(1)2.jpg]

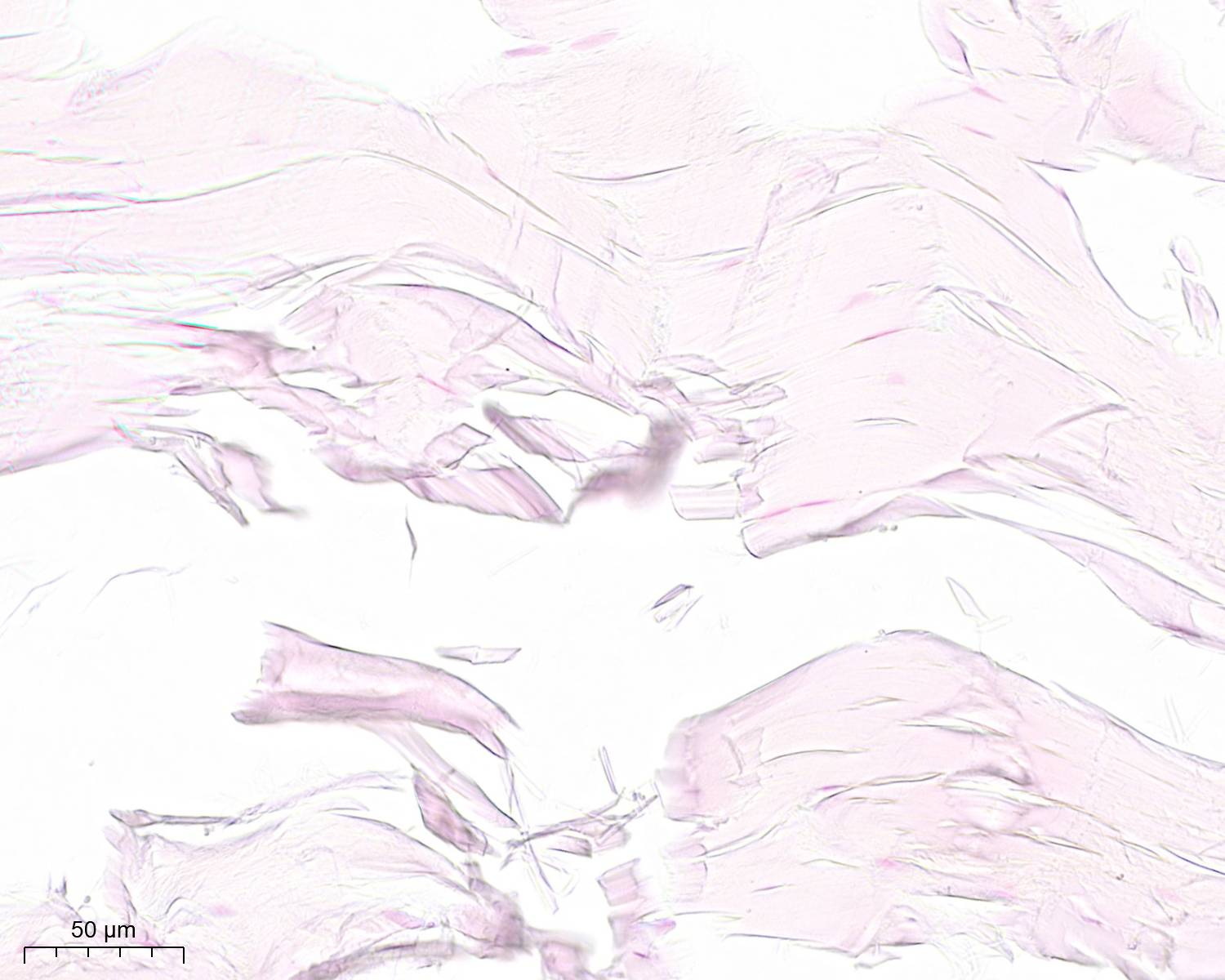

Supplement: Supplementary file 1 [file Data_Sheet_1.zip › Morphological figure/30min 02(1).jpg]

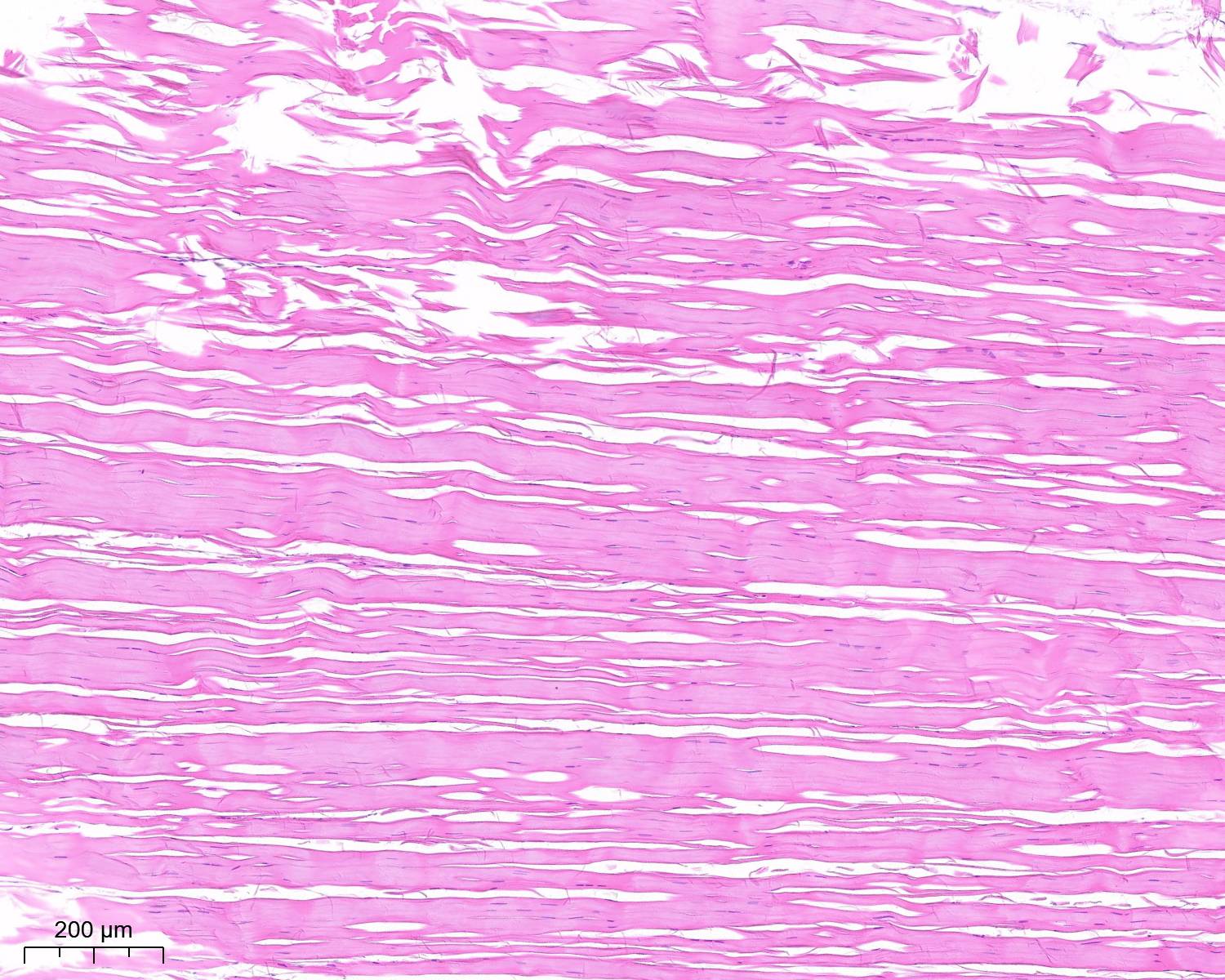

Supplement: Supplementary file 1 [file Data_Sheet_1.zip › Morphological figure/7min 01(1).jpg]

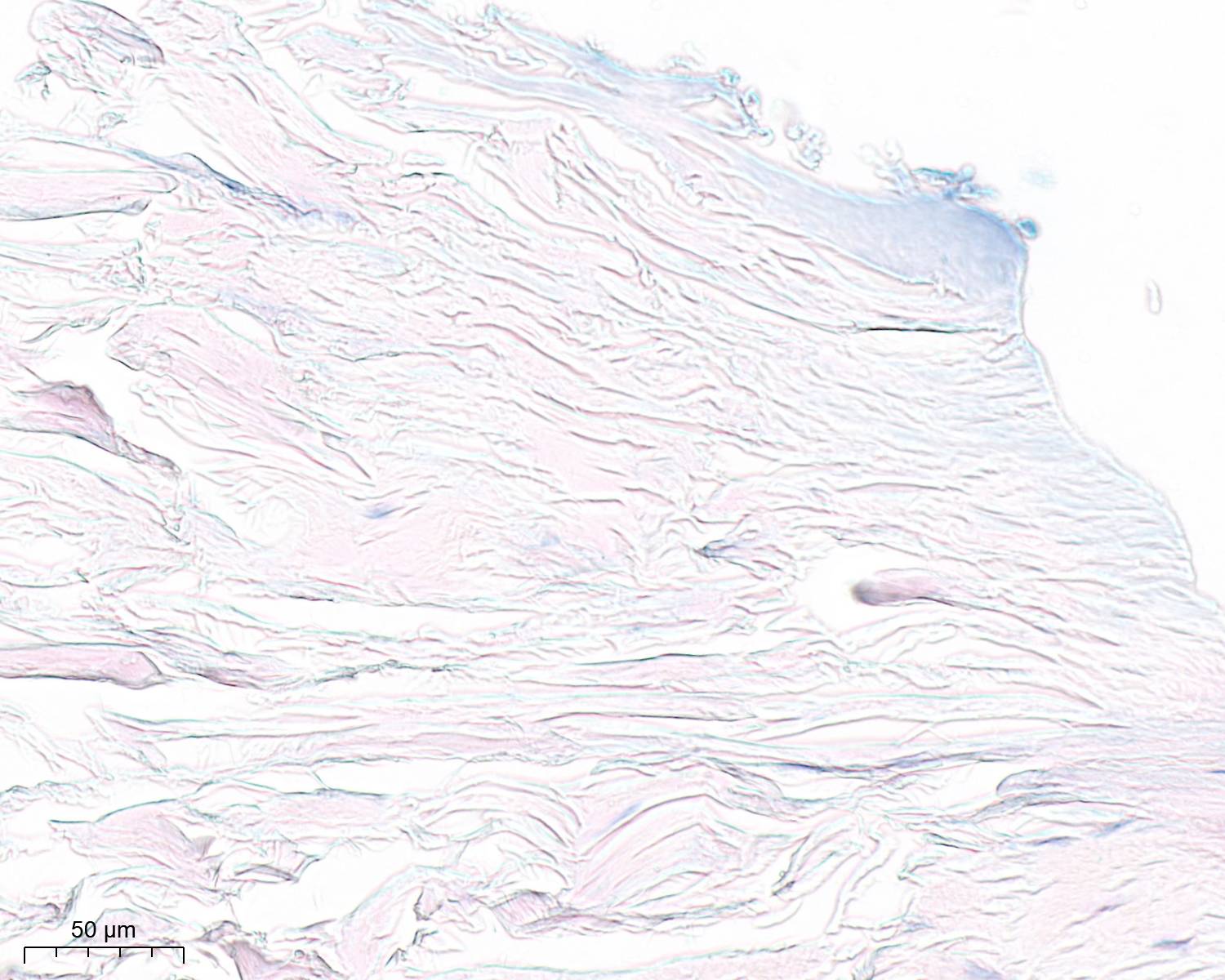

Supplement: Supplementary file 1 [file Data_Sheet_1.zip › Morphological figure/7min 02(1).jpg]

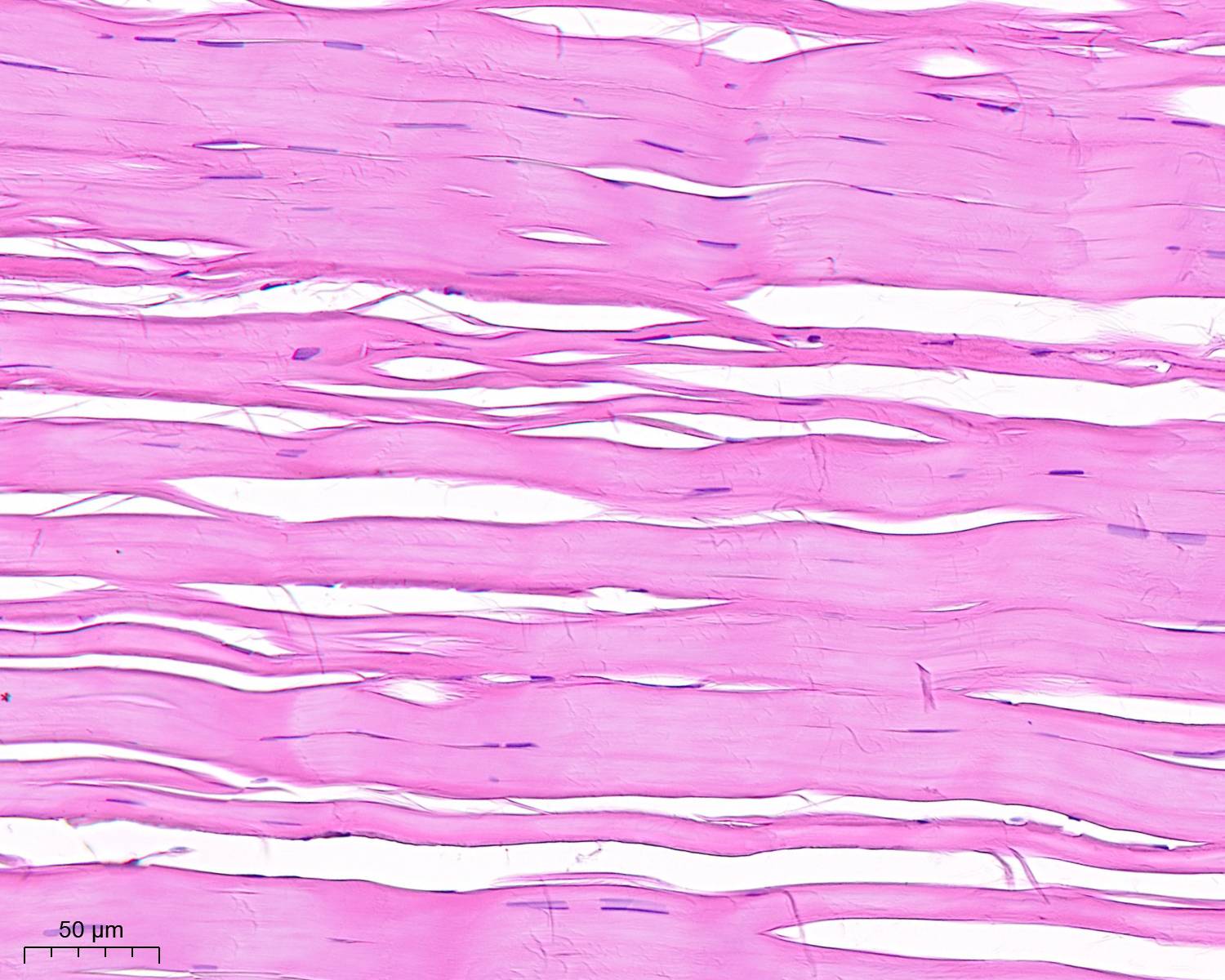

Supplement: Supplementary file 1 [file Data_Sheet_1.zip › Morphological figure/he 11min(1).jpg]

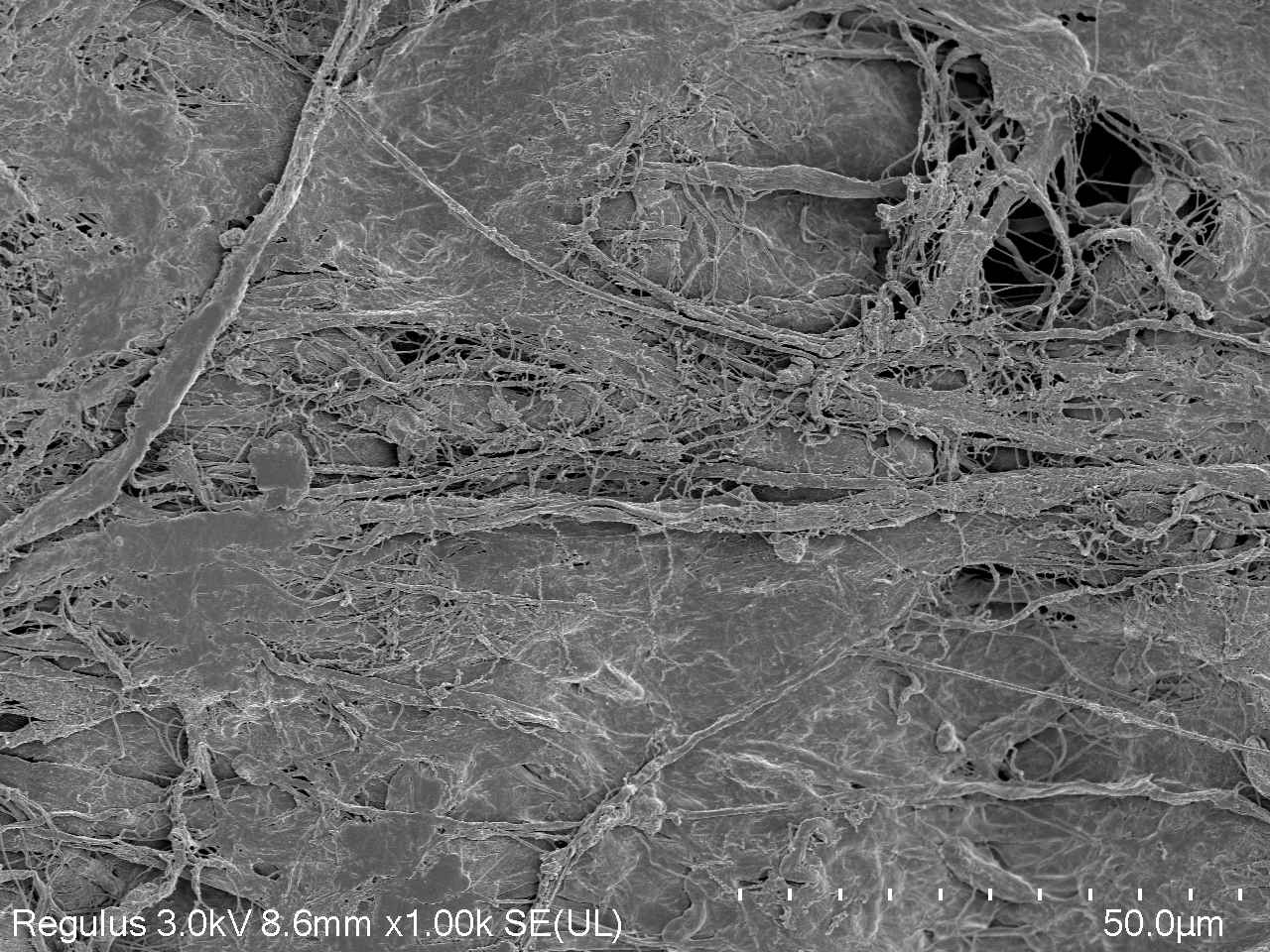

Supplement: Supplementary file 1 [file Data_Sheet_1.zip › Morphological figure/sem 11min(1).jpg]

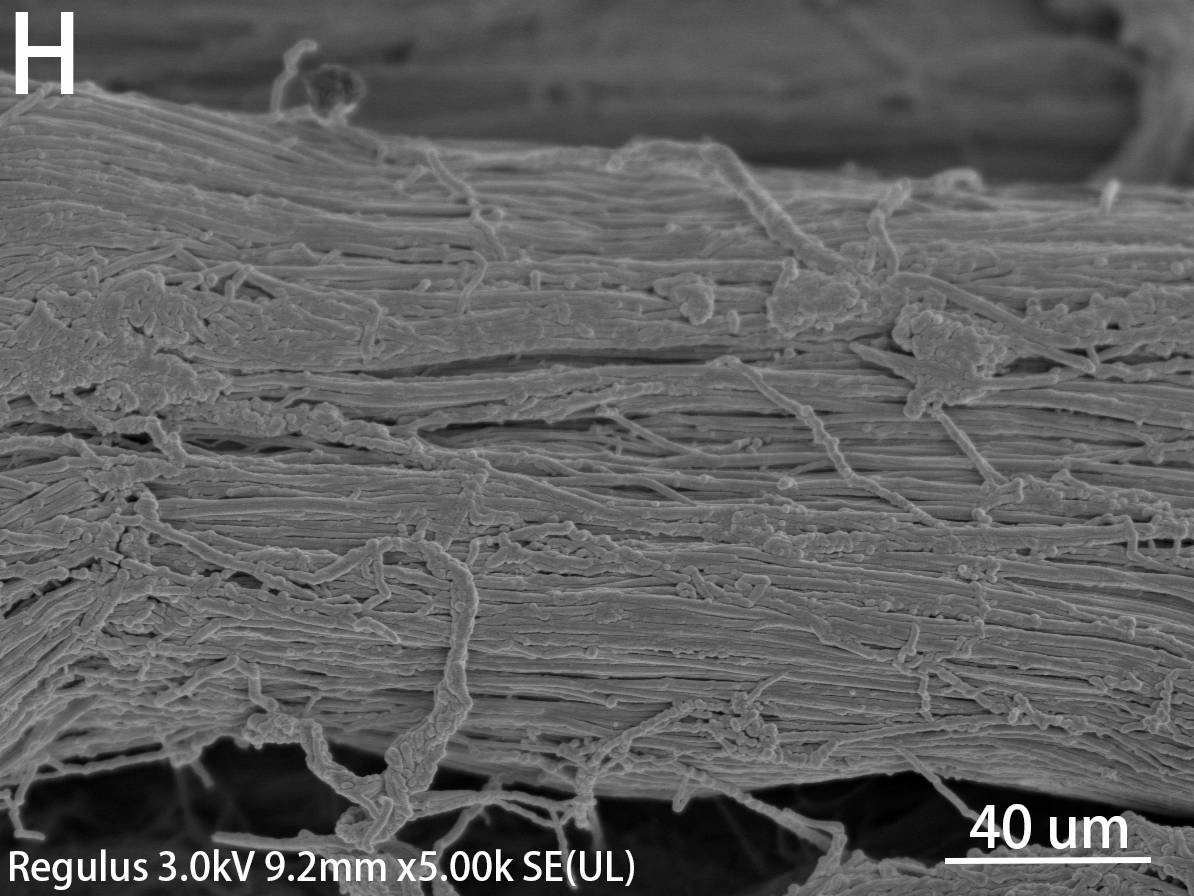

Supplement: Supplementary file 1 [file Data_Sheet_1.zip › Morphological figure/sem 18 min(1).jpg]

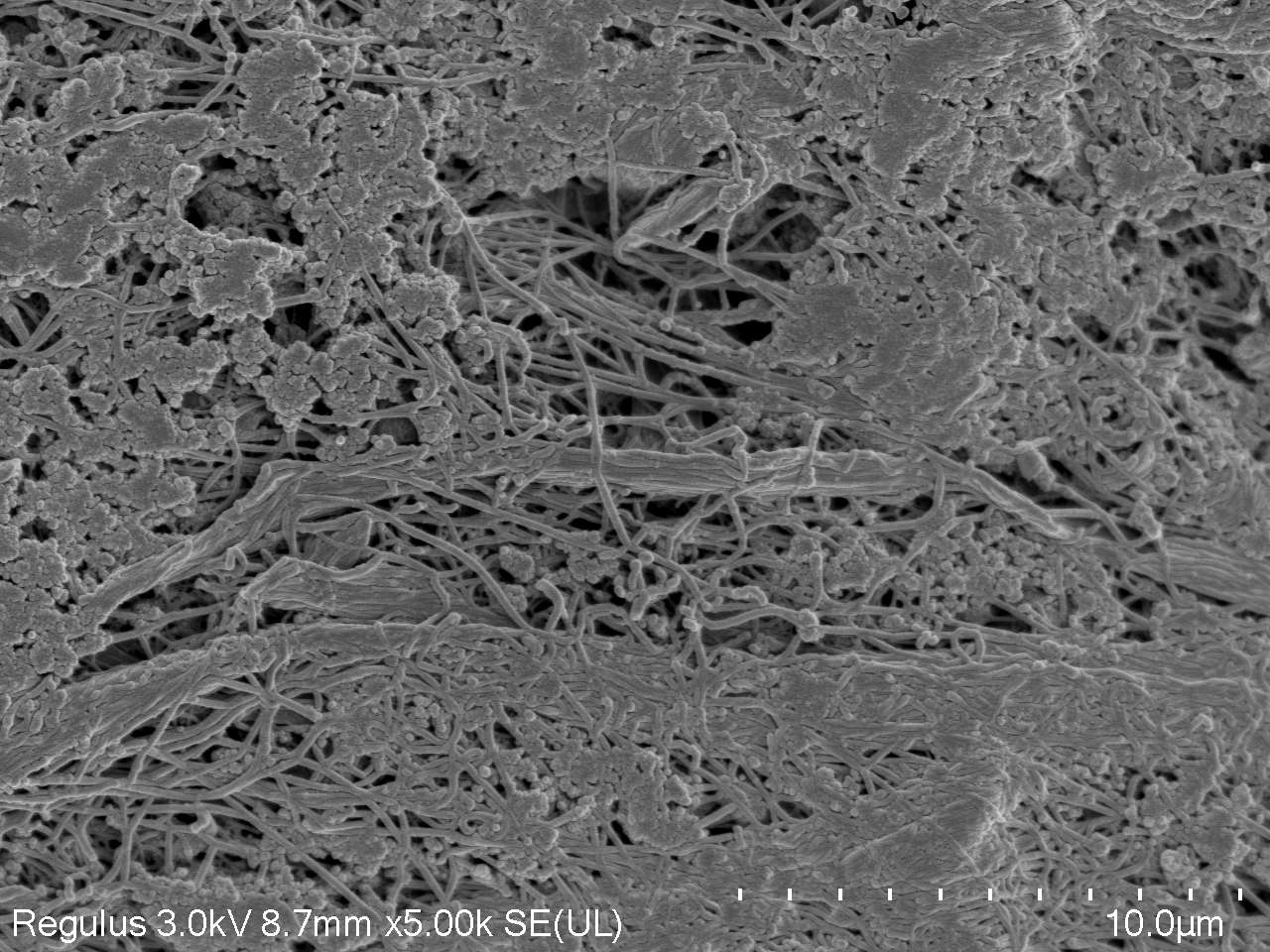

Supplement: Supplementary file 1 [file Data_Sheet_1.zip › Morphological figure/sem 30min (1).jpg]

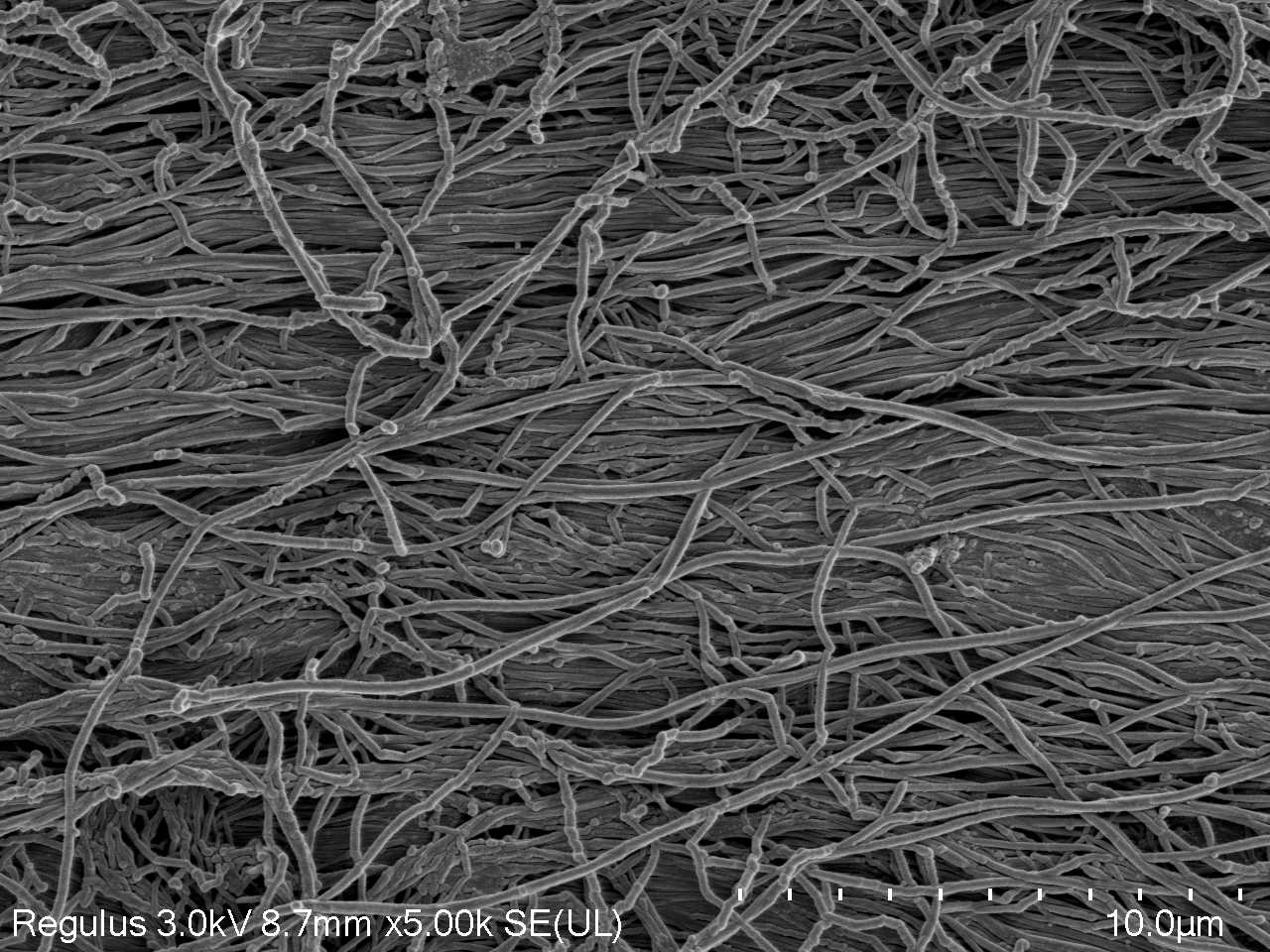

Supplement: Supplementary file 1 [file Data_Sheet_1.zip › Morphological figure/sem7 min 02(1).jpg]
